# Unveiling causal immune cell–gene associations in multiple myeloma: insights from systematic reviews and Mendelian randomization analyses

**DOI:** 10.3389/fmed.2025.1456732

**Published:** 2025-01-22

**Authors:** Hui Zhang, Ling Zhang, Jing-Xuan Lian, Zhi-Fu Kou, Yu Zhu, Li-Tian Ma, Jin Zheng, Can-Jun Zhao

**Affiliations:** ^1^Department of Traditional Chinese Medicine, Tangdu Hospital, Air Force Medical University (Fourth Military Medical University), Xi’an, China; ^2^Key Laboratory of Integrated Traditional Chinese and Western Medicine Tumor Diagnosis and Treatment in Shaanxi Province, Xi’an, China; ^3^Department of Otorhinolaryngology Head and Neck Surgery, Tangdu Hospital, Air Force Medical University (Fourth Military Medical University), Xi’an, China; ^4^Department of Hematology, Xijing Hospital, Air Force Medical University (Fourth Military Medical University), Xi’an, China; ^5^Department of Gastroenterology, Tangdu Hospital, Air Force Medical University (Fourth Military Medical University), Xi’an, China; ^6^College of Health, Dongguan Polytechnic, Dongguan, China; ^7^The Second Affiliated Hospital of Guangzhou University of Chinese Medicine, Guangzhou, China; ^8^Department of Thoracic Surgery, Tangdu Hospital Air Force Medical University (Fourth Military Medical University), Xi’an, China

**Keywords:** immune cells, Mendelian randomization, multiple myeloma, summary data-based Mendelian randomization, meta-analysis

## Abstract

**Background:**

The efficacy of novel chimeric antigen receptor T-cell (CAR-T) therapy is inconsistent, likely due to an incomplete understanding of the tumor microenvironment (TME). This study utilized meta-analysis to evaluate CAR-T-cell therapy efficacy and safety and employed two-sample Mendelian randomization (MR) analysis to investigate the causal links between immune cells and Multiple Myeloma (MM).

**Method:**

Our literature review, conducted from January 1, 2019, to August 30, 2024, across Medline/PubMed, Scopus, and Web of Science, identified 2,709 articles, 34 of which met our inclusion criteria. We utilized MR analysis of GWAS data to identify immune cells causally related to multiple myeloma, followed by SMR analysis to highlight associated pathogenic genes and colocalisation analysis for validation.

**Results:**

The meta-analysis revealed an 82.2% overall response rate to CAR-T-cell therapy, characterized by a safe profile with a grade 3 or higher CRS of 6.3% and neurotoxicity of 0.9%. BCMA, CD38, and GPRC5D CAR-T-cell therapies had superior response rates, whereas BCMA and CD3 CAR-T-cell therapy rates lagged at 61.8%. Post-adjustment for multiple testing, the levels of seven types of immune cells (two types of Treg, two types of TNBK, two types of B cells, and one type of Myeloid cell) were found to be elevated in association with an increased risk of multiple myeloma (MM), while the levels of another eight types of immune cells (one types of Treg, three types of TNBK, one type of MT cells, and two types of Myeloid cell and one type of cDC cells) were demonstrated to be associated with a decreased risk of MM. As supported by sensitivity analysis. SMR analysis pinpointed the risk genes VDR, VHL, POMC, and FANCD2, with VHL and POMC correlating at the methylation level. VDR was not significantly correlated with MM after correction for multiple tests. NCAM1 also exhibited a significant methylation-level association with disease.

**Conclusion:**

Our study supports the efficacy and safety of CAR-T-cell therapy in rrMM patients, with an 82.2% ORR and low rates of severe CRS (6.3%) and neurotoxicity (0.9%). This finding also suggests that BCMA/CD19 bispecific CAR-T cells have a superior ORR, pending clinical confirmation. MR analysis reveals links between immune cells, genes such as VDR and VHL, and MM, enhancing our understanding of its pathophysiology.

## 1 Introduction

Multiple myeloma (MM), which ranks second among hematologic malignancies, is characterized by clinical features such as anemia, renal dysfunction, and pathological fractures (1). The therapeutic evolution for MM over three decades has included autologous stem cell transplantation, proteasome inhibitors, immunomodulatory drugs, and monoclonal antibodies, leading to substantial improvements in patient quality of life and survival (2). Despite the increasing survival rates, a definitive cure for MM has yet to be realized, with the majority of patients ultimately succumbing to disease relapse (3). In relapsed/refractory multiple myeloma (rrMM), strategies targeting immune cells within the tumor microenvironment (TME) are promising therapeutic options (4). The TME consists of various effector and suppressor cells, including T cells, NKT cells, γδ T cells, and NK cells (5). Gene-engineered T cells, particularly CAR-T cells, have shown potential in treating rrMM by reprogramming T cells to target cancer-specific antigens. Since the 1980s, CAR-T-cell therapy has undergone significant development, with a surge in clinical trials and real-world data in recent years aimed at assessing its therapeutic benefits and risks (6). However, the efficacy of immune cell targeting in MM is limited by our incomplete understanding of TME complexity, indicating a need for further research to enhance the effectiveness of these targeted therapies.

Mendelian randomization (MR) employs genetic variants as instrumental variables to investigate causality between exposures and outcomes, effectively addressing confounding and bias in observational research (7). Genome-wide association studies (GWASs) identify trait-associated single-nucleotide polymorphisms (SNPs) and, when combined with gene expression and methylation data, reveal expressed or methylated quantitative trait loci (eQTLs or mQTLs), enhancing our understanding of genetic influences on phenotypic traits (8, 9). Summary-based Mendelian randomization (SMR) enhances the identification of pathogenic genes by integrating GWAS summary statistics with QTL data. Coupled with HEIDI’s heterogeneity-independent instrumental variable tests, this approach allows for the extraction of causal signals from genomic linkage disequilibrium.

In this study, we initially conducted a systematic review and meta-analysis based on the latest evidence to comprehensively assess the efficacy and safety of innovative CAR-T-cell therapy in patients with rrMM, thereby better guiding clinical decision-making and strengthening clinical recommendations. We subsequently employed an MR approach to identify immune cells causally associated with MM. By integrating multiomics data using SMR and colocalisation analysis, we systematically explored the potential causal relationships between different immune cells and MM. Through MR analysis and multiomics data integration, we aim to provide new perspectives for etiological research in MM and offer scientific evidence for developing novel diagnostic and therapeutic strategies.

## 2 Methods

### 2.1 Section of systematic review and meta-analysis

The meta-analysis component of this study was conducted in accordance with the Preferred Reporting Items for Systematic Reviews and Meta-Analyses (PRISMA) guidelines.

#### 2.1.1 Eligibility criteria

In this review, studies published from January 1, 2019, to August 31, 2024, were considered for inclusion based on the PICO framework (10). The eligibility criteria were as follows: (1) study types: clinical trials and cohort studies, both prospective and retrospective; (2) population: patients aged 18 years or older with rrMM; (3) intervention: CAR-T-cell therapy, regardless of the specific antigen targeted; (4) outcomes: at least one efficacy assessment and one safety assessment. Efficacy outcomes included the overall response rate (ORR), complete response rate (CRR), very good partial response (vgPR), partial response (PR), and progressive disease (PD). The CRR encompasses stringent complete response (sCR) and complete response (CR). The ORR, defined by the International Myeloma Working Group (IMWG) response criteria (11), represents the proportion of patients who achieve a partial response (PR) or better. Safety outcomes included cytokine release syndrome (CRS), neurotoxicity, hematology-related adverse events (neutropenia, leukopenia, anemia, thrombocytopenia, and lymphopenia), infections, and all-cause mortality. Except for all-cause mortality, all remaining safety outcomes were assessed for any grade and for those with a grade ≥ 3. The most commonly utilized criteria for grading the severity of adverse events in safety outcomes are the National Cancer Institute Common Terminology Criteria for Adverse Events (NCI CTCAE) versions 4.03 and 5.0 (12).

Studies were excluded from the analysis if they met the following criteria: (1) they were review articles, abstracts, conference reports, case–control studies, case reports, letters to the editor, or editorials; (2) publications were not in English; and (3) studies were conducted with nonhuman subjects. In cases of similar and duplicate clinical trials, only the study with the longest follow-up was included in the analysis. This review was limited to full-text articles; for those without full texts, contact was made with the authors.

#### 2.1.2 Information sources and search strategy

Articles were retrieved from Medline/PubMed, Scopus, and Web of Science up to August 31, 2024, for the period from January 1, 2019, to August 31, 2023, without language restrictions. The specific search strategy is detailed in the Supplementary material.

#### 2.1.3 Data extraction and processing

Data were manually collected by two independent reviewers using a predefined form. Once more, a consensus methodology was employed to resolve any disagreements. Details on data processing, assessment of risk of bias, and integration and statistical analysis of the data are provided in the Supplementary material.

### 2.2 Section of MR

#### 2.2.1 Study design

**Figure 1:**
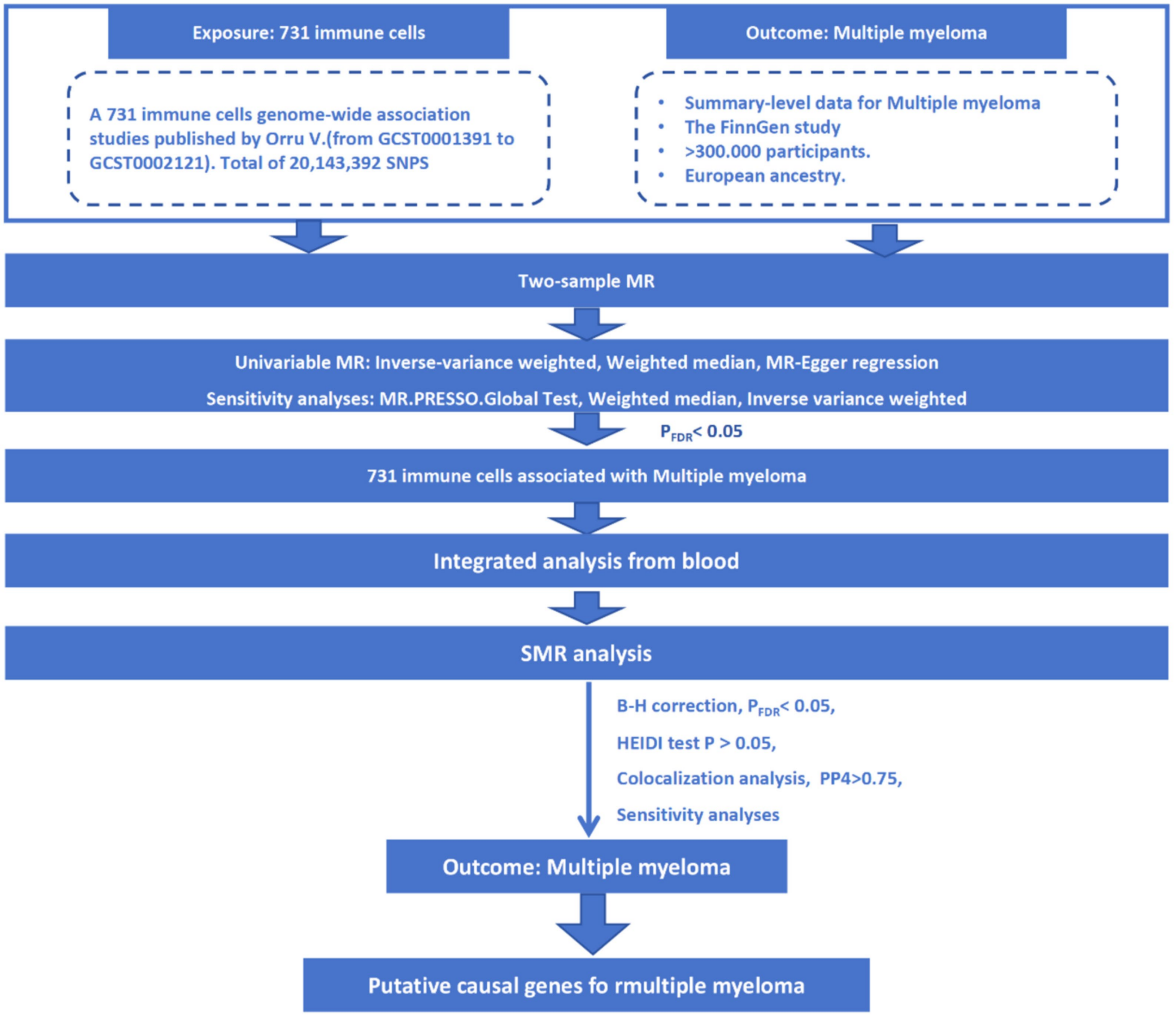
Flowchart of the analyses performed.

The study design is presented in Figure 1, which provides an overview of the research approach. Publicly available genome-wide association studies (GWASs) were utilized as the basis for this investigation. An initial two-sample Mendelian analysis identified immune cells with causal associations with MM. To explore the putative immune cell genes and their regulatory elements associated with MM risk, we employed the SMR approach by integrating cis-eQTL/cis-mQTL data (SMR, PFDR <0.05; HEIDI test *p* > 0.05).

#### 2.2.2 Immunity-wide GWAS data sources

The GWAS catalog provides aggregated statistics for 731 immune traits, from GCST0001391 to GCST0002121. Our study analyzed 731 immunophenotypes, including B cells, cDCs, mature T cells, monocytes, myeloid cells, TBNKs (T cells, B cells, and NK cells), and Treg cells. It utilized data from 3,757 Europeans, examining 20 million SNPs and indels by genotyping arrays or Sardinian reference panels, adjusting for covariates such as sex, age, and age squared. GWASs on MM were derived from publicly accessible databases, primarily from the FinnGen project. This extensive initiative involves collecting and analyzing genetic data from over 500,000 participants within the Finnish Biobank. Details regarding all QTL and GWAS datasets utilized in this study are presented in Supplementary Table S1.

#### 2.2.3 Selection of instrumental variables

Adopting standards from current research, our MR study curated IVs for immune traits at a 1.00E−5 significance level, utilizing PLINK v1.90 to filter out those in strong LD (*r*^2^ > 0.1) within 500 kb, based on the 1,000 Genomes Project. We enforced a stringent threshold of 5.00E−8 and *r*^2^ ≤ 0.01 for MM. IV strength was validated through F-statistics, and we controlled for multiple testing using the Benjamini–Hochberg procedure, considering *p* < 0.01. Detailed methods can be found in the Supplementary material.

#### 2.2.4 Statistical analysis

The primary analysis consisted of three phases: a two-sample Mendelian analysis, a primary SMR analysis, and a colocalisation analysis. Data were standardized by excluding ambiguous and palindromic SNPs. The primary MR analysis used the IVW method, assuming valid instruments, with fixed-effects meta-analyses to combine estimates. Heterogeneity was assessed using I^2^ statistics and Cochran’s Q tests, with sensitivity analyses including weighted median, MR–Egger, and MR-PRESSO to ensure consistency and detect pleiotropy (13). Detailed methods can be found in the Supplementary material.

#### 2.2.5 Summary-data-based MR

SMR was applied to evaluate the pleiotropic effects of genetic variants on disease phenotypes. Using SMR software version 1.0.3 with default settings, the analysis prioritized genes using GWAS data and cis-eQTLs as instrumental variables. LD estimates referenced the European Ancestry Genome Consortium (14). The HEIDI test, with a significance threshold of *p* < 0.05, was used to identify associations likely due to linkage disequilibrium rather than pleiotropy, which were subsequently excluded from the analysis (Supplementary material) (15).

#### 2.2.6 Colocalization analysis

We utilized the colocation method to assess shared causal variants between traits within genomic regions. A Bayesian analysis with the ‘coloc’ R package (version 5.1.0) was used to estimate the posterior probability of shared genetic effects, enhancing the resolution of our findings. To access the ‘coloc’ package, please visit https://chr1swallace.github.io/coloc/ (16). A PP.H4 value greater than 0.75 was considered a robust threshold, indicating strong evidence supporting colocalisation between GWAS and QTL associations (Supplementary material).

## 3 Results

### 3.1 Results for meta-analysis

#### 3.1.1 Study selection

**Figure 2:**
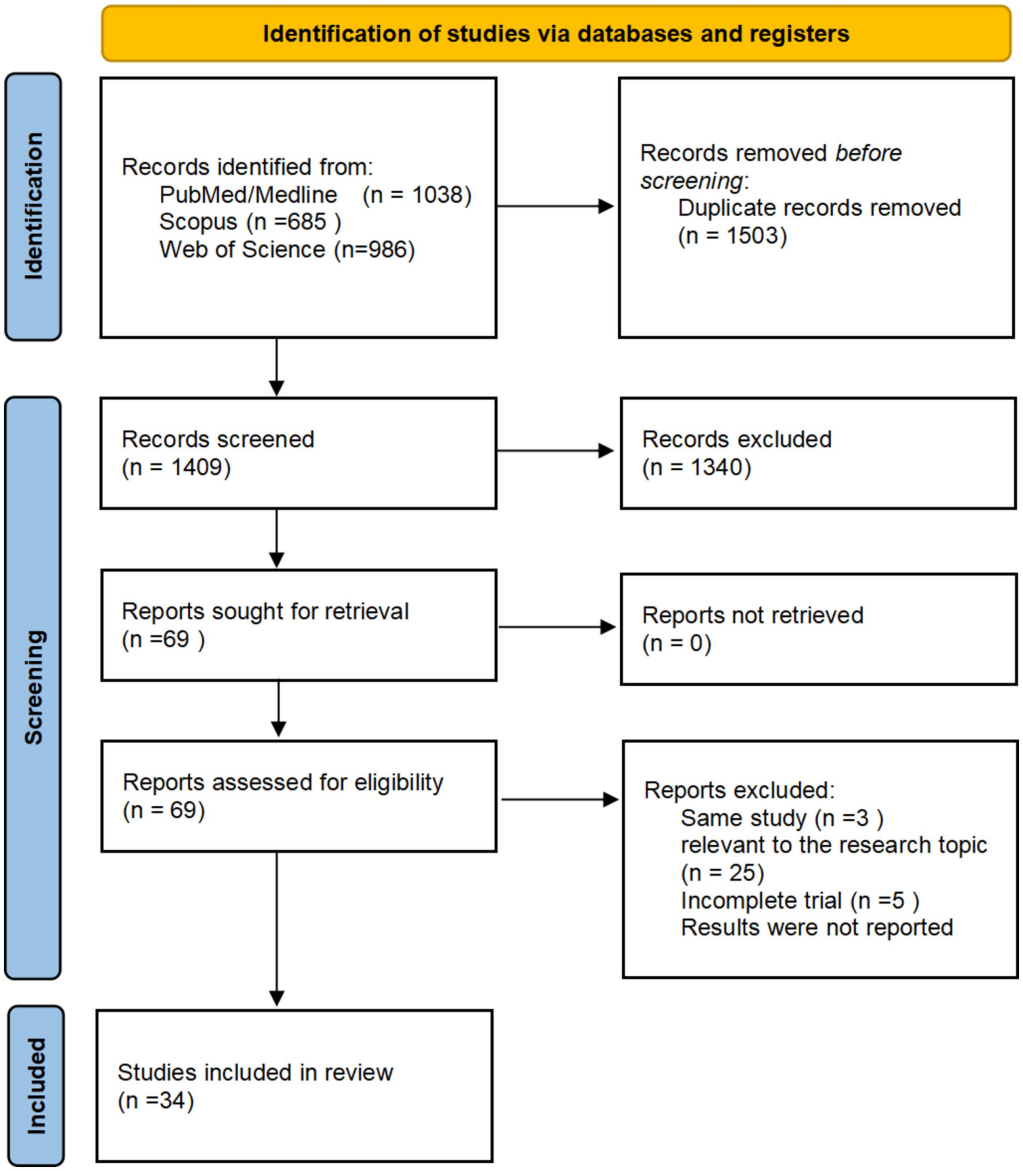
PRISMA Flow chart showing the process for inclusion of studies about MM.

A search across the PubMed, Scopus, and Web of Science databases identified 2,912 potentially relevant articles. Following the exclusion of 1,503 duplicates, 1,409 articles were screened by title and abstract, leading to 52 full-text assessments. Ultimately, 34 articles fulfilled the criteria for inclusion in this review (17–50). Figure 2 illustrates the study selection process in a PRISMA flowchart.

These 34 articles, published between 2019 and 2024, comprised 32 prospective and 2 retrospective studies. Within this cohort, 2 studies evaluated real-world data: 17 were phase I clinical trials, 5 were phase I/II trials, and 10 were phase II trials. Geographically, 10 studies were conducted solely in the United States, the majority in China, with an additional 5 conducted across multiple countries. The collective data encompass 1,388 MM patients treated with CAR-T-cell therapy, primarily targeting BCMA in 21 studies, with the remainder focusing on dual targets and GPRC5D. The number of CAR-T cells used varied across studies, and the follow-up periods ranged from 136 days to 48 months. (Supplementary Table S2).

#### 3.1.2 Efficacy outcomes

**Figure 3:**
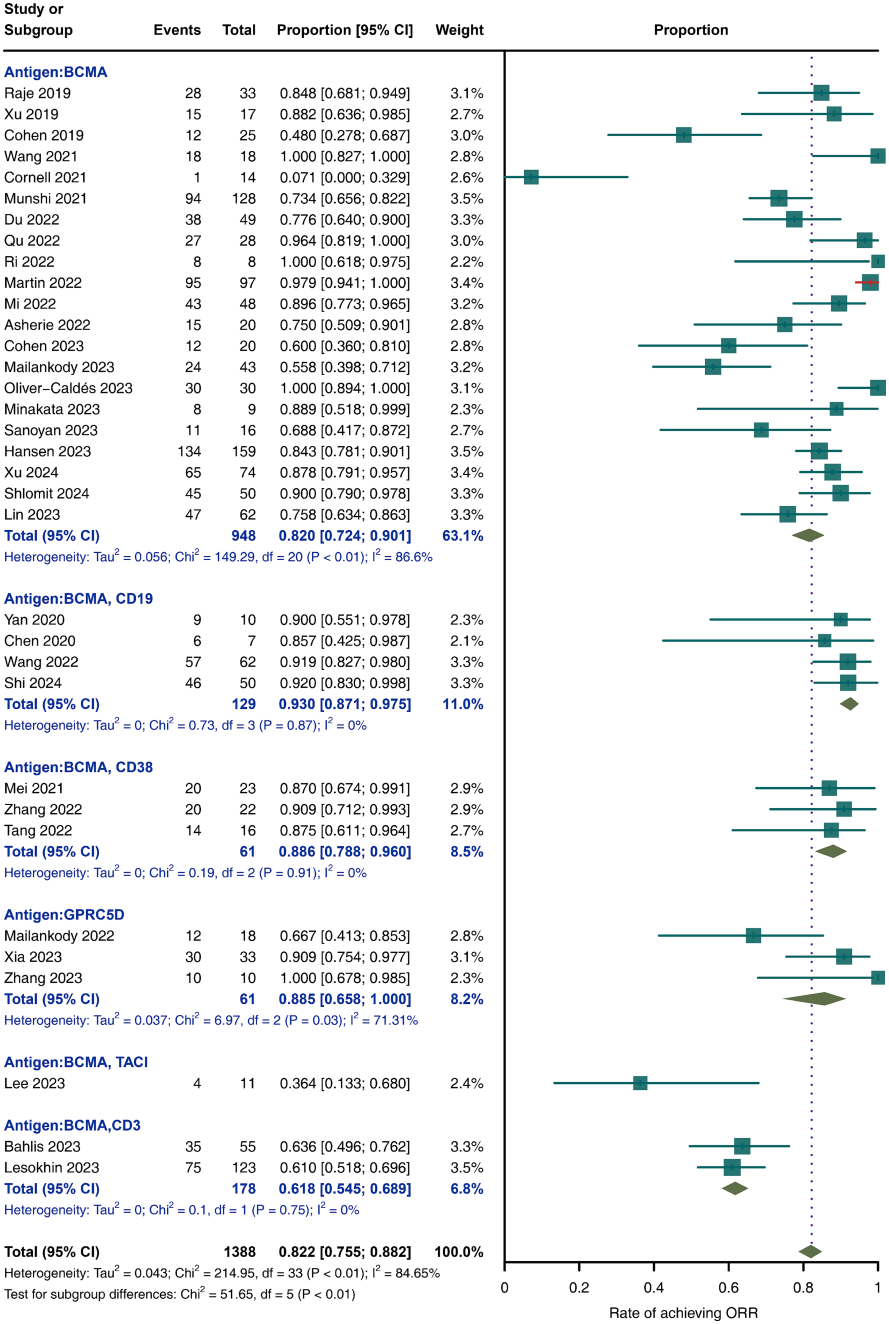
Meta-analysis of ORR in MM patients treated with CAR-T-cell therapy.

As shown in Figure 3, across all included studies involving 1,388 patients, the reported overall response rate (ORR) was 82.2% (95% CI, 75.5–88.2; *I*^2^ = 84.65%). Subgroup analysis indicated that BCMA/CD19 bispecific CAR-T-cell therapy achieved the highest ORR of 93% (95% CI, 87.1–97.5; *I*^2^ = 0%), whereas BCMA/CD38-specific CAR-T-cell therapy had the lowest ORR of 61.8% (95% CI, 54.5–68.9; *I*^2^ = 0%). Other targets, such as BCMA/CD38 and GPRC5D, presented intermediate ORRs of 88.6% (95% CI, 78.8–96.0; *I*^2^ = 0%) and 88.5% (95% CI, 65.8–100; *I*^2^ = 71.31%), respectively.

**Figure 4:**
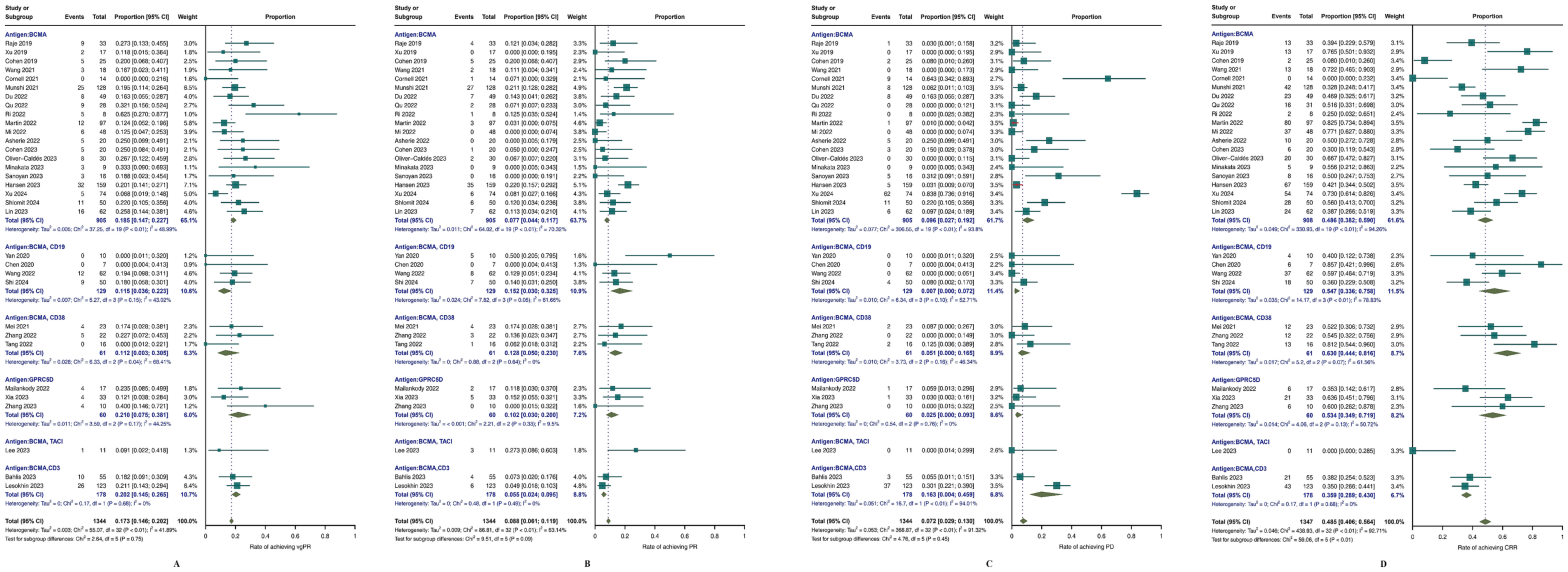
Meta-analysis of efficacy outcomes in MM patients treated with CAR T-cell therapy. **(A)** complete response rate CRR; **(B)** progressive disease (PD); **(C)** partial response (PR); **(D)** very good partial response (vgPR).

For other outcomes, as shown in Figure 4, the meta-analysis results were 45.8% (95% CI, 40.6–56.4; *I*^2^ = 92.71%) for CRR, 17.3% (95% CI, 14.6–20.2; *I*^2^ = 40.89%) for vgPR, 8.8% (95% CI, 6.1–11.9; *I*^2^ = 63.14%) for PR, and 7.2% (95% CI, 2.9–13.0; *I*^2^ = 91.32%) for PD. Subgroup analysis revealed that bispecific BCMA/CD3 CAR-T-cell therapy had superior results for vgPR (20.2, 95% CI, 14.5–26.5; *I*^2^ = 0%) compared to the overall meta-analysis effect but inferior outcomes for CRR (35.9, 95% CI, 28.9–43.0; *I*^2^ = 0%), PR (5.5, 95% CI, 2.4–9.5; *I*^2^ = 63.14%), and PD (16.3, 95% CI, 0.04–45.9; *I*^2^ = 94.01%).

#### 3.1.3 Safety outcomes

**Figure 5:**
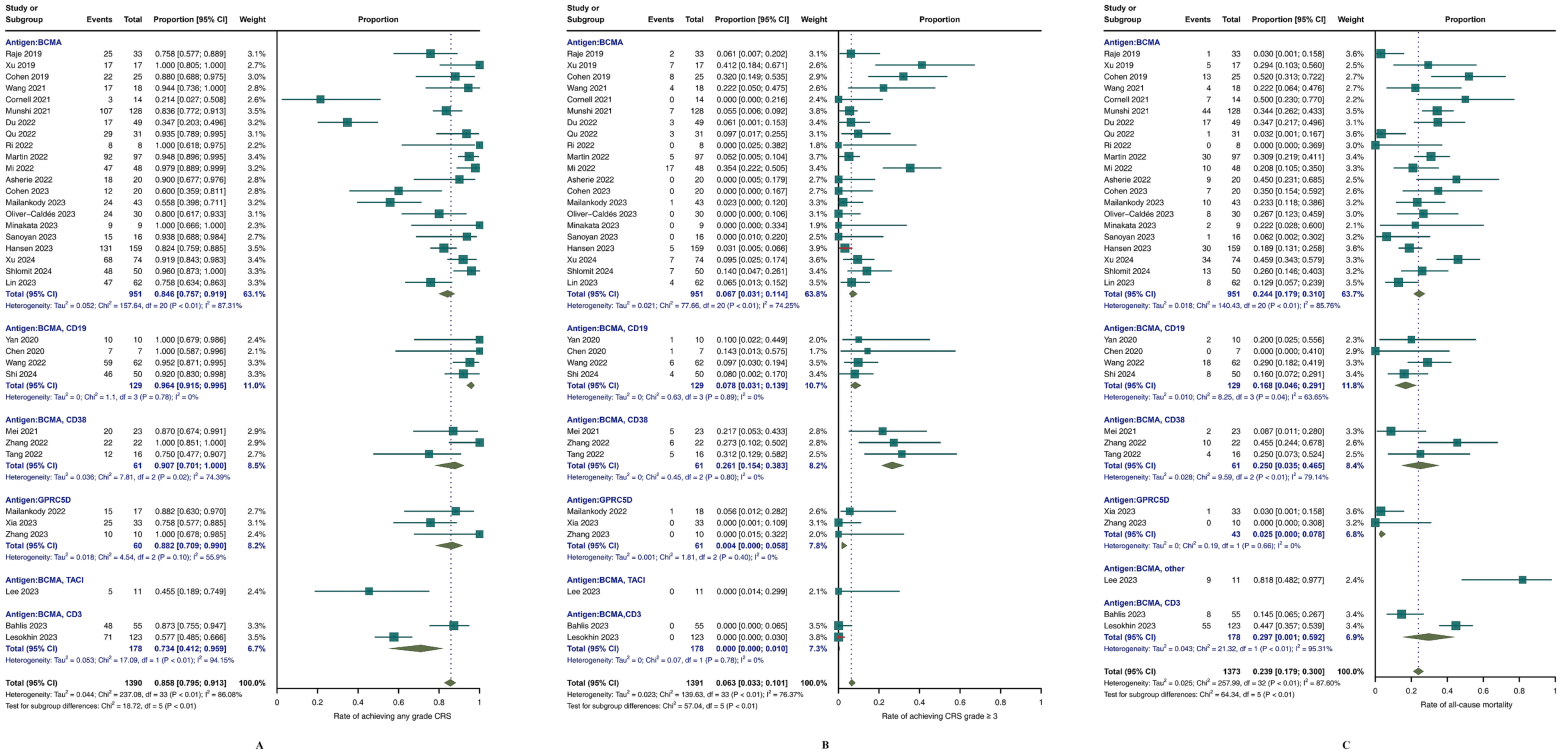
Meta-analysis of safety outcomes in MM patients treated with CAR-T-cell therapy. **(A)** Meta-analysis of CRS of any grade; **(B)** meta-analysis of CRS ≥3; **(C)** meta-analysis of all-cause mortality.

In terms of safety, as depicted in Figure 5, all studies reported rates of any-grade and grade ≥ 3 CRS. Specifically, the proportion of any-grade CRS (Figure 5A) was 85.8% (95% CI, 79.5–91.3; *I*^2^ = 86.8%), and for grade ≥ 3 CRS (Figure 5B), the percentage was 6.3% (95% CI, 3.3–10.1; *I*^2^ = 76.37%). The incidence of any-grade neurotoxicity was 9.8% (95% CI, 5.9–14.3; *I*^2^ = 78.95%), and that of grade ≥ 3 neurotoxicity was 0.9% (95% CI, 0.1–2.2; *I*^2^ = 39.2%). Hematological adverse events were also assessed, including any-grade neutropenia, leukopenia, anemia, thrombocytopenia, lymphopenia, and grade ≥ 3 events. The incidence of infection events was also analysed, with specific results presented in Supplementary Figures S1, S2. Among the 33 studies reporting all-cause mortality (Figure 5C), the aggregate rate was 23.34% (95% CI, 16.57–30.79; *I*^2^ = 78%). Subgroup analysis revealed that the lowest percentage of patients receiving GPRC5D CAR-T-cell therapy was 2.5% (95% CI, 0–7.8%; *I*^2^ = 0%), whereas BCMA/CD19 CAR-T-cell therapy was 16.8% (95% CI, 4.6–29.1%; *I*^2^ = 63.65%).

#### 3.1.4 Risk of bias in the included studies

The MINORS score was utilized to evaluate study quality, with all studies scoring greater than 10 points, reflecting high methodological standards. Interrater reliability between two reviewers, assessed by Cohen’s kappa coefficient, was substantial, with all values exceeding 0.6 and an average of 0.85, indicating consistent review outcomes (Supplementary Table S3).

**Figure 6:**
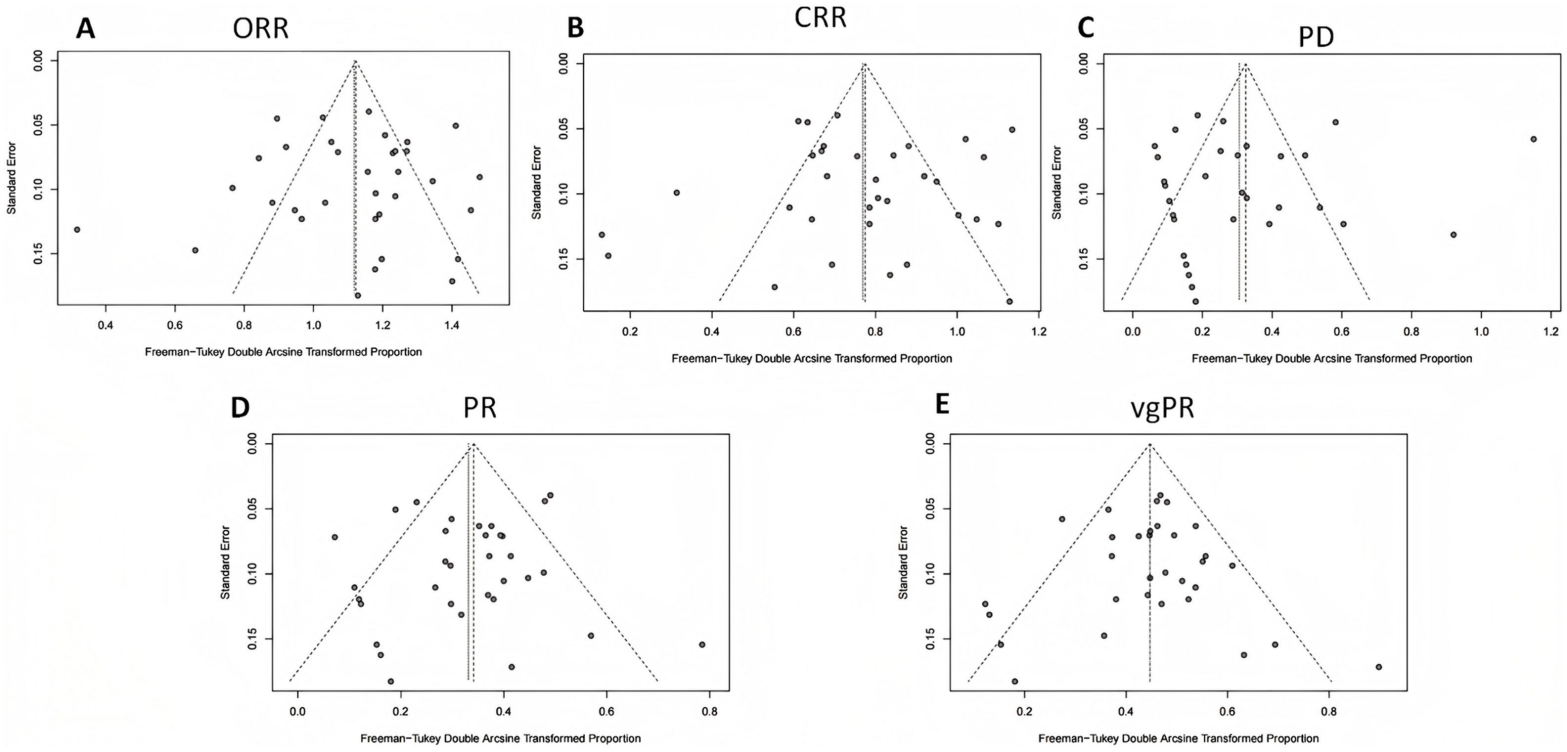
Funnel plot analysis for publication bias in efficacy outcomes. **(A)** Overall response rate (ORR); **(B)** complete response rate (CRR); **(C)** progressive disease (PD); **(D)** partial response (PR); **(E)** very good partial response (vgPR).

Publication bias was evaluated by visually examining funnel plots for ORR, CRR, PD, PR, and vgPR, with Figure 6 illustrating an asymmetrical distribution that hints at potential bias. Subsequent Egger’s and Begg’s tests identified a significant publication bias specifically for PD; other endpoints did not exhibit substantial evidence of bias.

### 3.2 Results for MR

**Figure 7:**
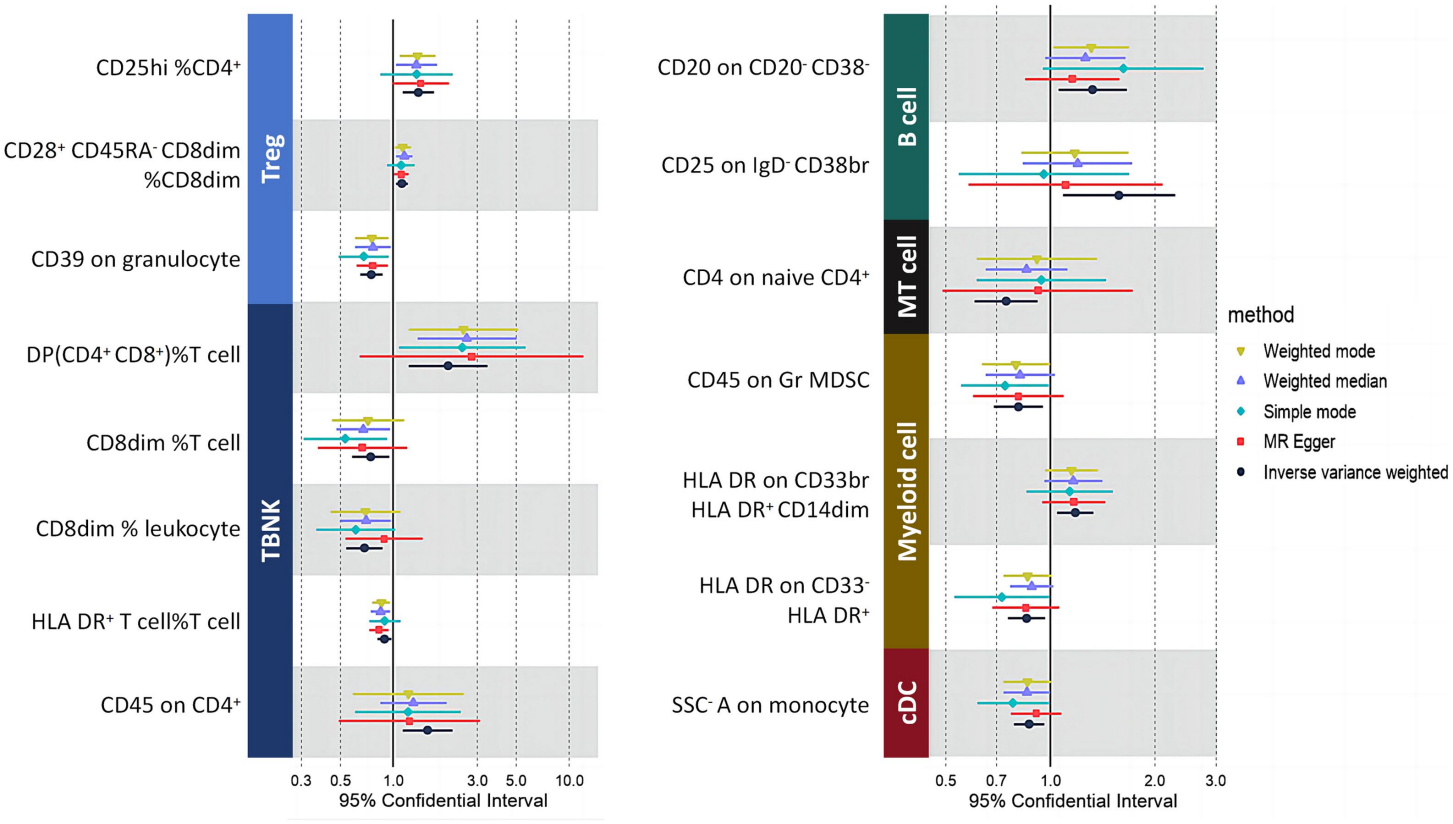
The results indicate that only immune cells exhibit a significant association with MM after BH correction (P_FDR_ < 0.05). Each color represents a distinct immune cell type. MT cells, maturation stages of T cells.

The findings of the two-sample MR results, as summarized in Table 1, identified 15 immune cells as causal factors for MM after Bonferroni–Holm (BH) correction. Specifically, three Treg cell subtypes were identified, with two linked to an increased risk and one showing a protective effect. Five TBNK cell subtypes were also identified, with two posing a risk and one offering protection. Both B-cell types were associated with a greater risk for the disease. Interestingly, T-cell maturation stages were found to be protective. Among myeloid cells, one type was identified as a risk factor, whereas the other had a protective effect. A single cDC type was observed, and no association was found with monocytes. The results from multiple MR analysis methods, as illustrated in Figure 7, consistently revealed a unidirectional effect, reinforcing the reliability of our conclusions. Detailed results are available in Supplementary Table S4. Sensitivity analyses ruled out horizontal pleiotropy (P MR–Egger intercept >0.05; Supplementary Table S5), and a global test confirmed its absence. Heterogeneity analyses revealed mostly mild heterogeneity (*I*^2^ < 25%), with one immune cell result showing moderate heterogeneity (25% < *I*^2^ < 75%). No results exhibited high heterogeneity, further validating the robustness of our results.

**Table 1:** Causal relationship between immune cells and MM through IVW analysis.

Exposure	Panel	NSNP	Beta	SE	PVAL	OR	95% CI (low)	95% CI (up)	P_FDR_
CD25hi %CD4+	Treg	16	0.327	0.105	1.85E-03	1.387	1.129	1.703	0.097
CD28+ CD45RA- CD8dim %CD8dim	Treg	20	0.111	0.04	1.41E-03	1.118	1.034	1.209	0.079
CD39 on granulocyte	Treg	17	−0.289	0.075	5.03E-05	0.749	0.647	0.866	0.037
DP (CD4 + CD8+) %T-cell	TBNK	4	0.718	0.266	4.08E-04	2.05	1.217	3.452	0.05
CD8dim %T-cell	TBNK	15	−0.297	0.125	1.18E-03	0.743	0.581	0.95	0.072
CD8dim %leukocyte	TBNK	13	−0.379	0.121	1.14E-04	0.685	0.54	0.868	0.042
HLA DR+ T cell %T cell	TBNK	25	−0.117	0.047	9.39E-04	0.89	0.811	0.977	0.062
CD45 on CD4+	TBNK	8	0.448	0.166	2.82E-04	1.566	1.131	2.167	0.052
CD20 on CD20- CD38-	B cell	8	0.279	0.116	7.30E-04	1.322	1.054	1.659	0.067
CD25 on IgD- CD38br	B cell	12	0.454	0.19	1.88E-03	1.574	1.085	2.283	0.092
CD4 on naive CD4+	MT cell	18	−0.293	0.107	2.55E-04	0.746	0.605	0.92	0.062
CD45 on Gr MDSC	Myeloid cell	8	−0.212	0.083	7.41E-04	0.809	0.687	0.953	0.06
HLA DR on CD33br HLA DR+ CD14dim	Myeloid cell	17	0.165	0.062	3.13E-04	1.18	1.044	1.333	0.046
HLA DR on CD33- HLA DR+	Myeloid cell	20	−0.158	0.063	8.30E-04	0.854	0.755	0.967	0.061
SSC-A on monocyte	cDC	32	−0.141	0.052	5.30E-04	0.869	0.785	0.961	0.055

The MR analysis method employed in the table was inverse variance weighted, and random or fixed effect models were selected based on the magnitude of heterogeneity. Only results with adjusted *p* < 0.10 were presented. In cases where certain outcomes did not meet the criteria (adjusted *p* < 0.10), only the result with the smallest adjusted *p* value was displayed. OR, odd ratio; CI, confidence interval.

#### 3.2.1 MR results from eQTL of immune cells causally associated with MM

**Figure 8:**
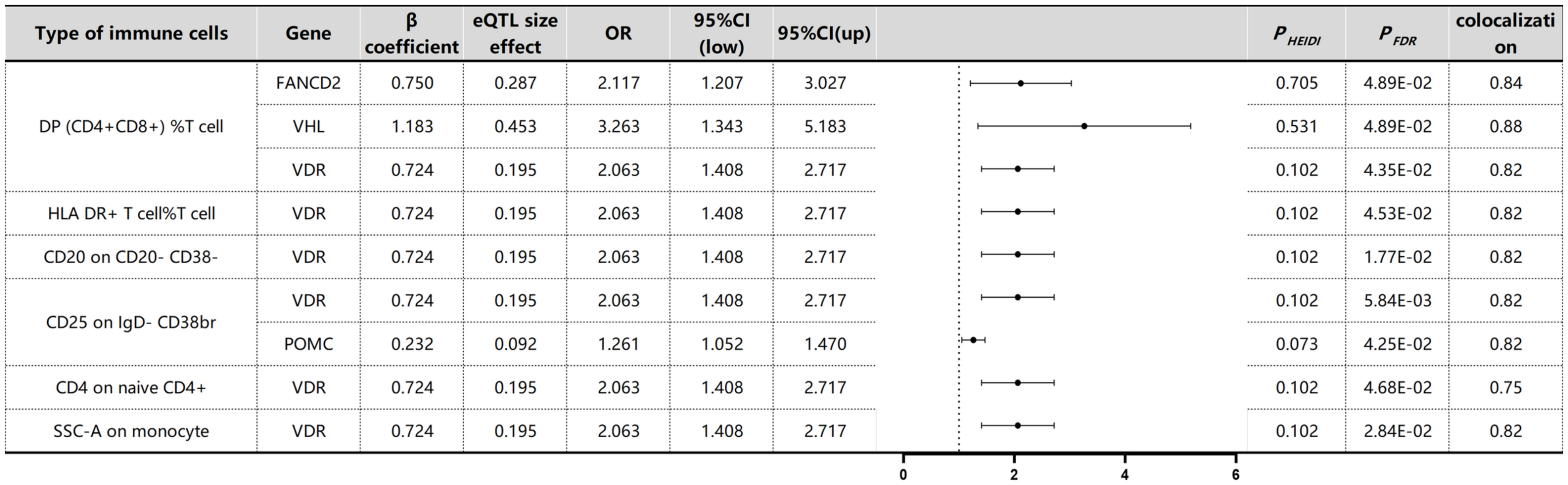
SMR and Colocalisation Results for eQTLs of immune cells with causal relationships to MM. *β* > 0 indicates a positive correlation, and β < 0 indicates a negative correlation. Ratios are calculated according to the expected value of the causal estimate (β coefficient). Colocalisation was determined by PP.H4 between eQTLs and MM, with a PP.H4 threshold of >0.75 considered strong evidence for colocalisation. The displayed results are limited to those with PP.H4 values of 0.70 or higher.

The associations between immune cells in the blood and MM were determined through SMR testing (Figure 8). To address potential genome-wide type I errors, we applied multiple-test adjustments to identify statistically significant associations (P_FDR_ < 0.05 Benjamini–Hochberg correction). To further explore this association, a HEIDI test (P_HEIDI_ > 0.05) was conducted using SMR software resulting from shared causal variation rather than pleiotropy (Supplementary Table S6). Through this approach, we successfully identified four genes associated with MM across six distinct immune cell types. The potential confounding effects of linkage disequilibrium (LD) were addressed through additional colocation analyses. A posterior probability (PP.H4) greater than 0.75 indicated robust evidence supporting the colocation between cancer genome-wide association studies (GWASs) and expressed quantitative trait loci (eQTLs). FANCD2 (OR 2.12, 95% CI, 1.21–3.03, P_FDR_ = 4.89 × 10^−2^) and VHL (OR 3.26, 95% CI, 1.34–5.18, P_FDR_ = 4.89 × 10^−2^) were identified as risk factors for MM in DP (CD4^+^CD8^+^) %T cells, whereas POMC (OR 1.26, 95% CI, 1.05–1.47, P_FDR_ = 4.25 × 10^−2^) was also found to be a risk factor for MM in CD25 on IgD- CD38br immune cells. Surprisingly, VDR was identified as a common risk factor for MM across all six immune cell types, suggesting the presence of a shared SNP in the VDR gene (OR 2.06, 95% CI, 1.41–2.72) associated with MM.

#### 3.2.2 MR results from mQTL of immune cells causally associated with MM

**Figure 9:**
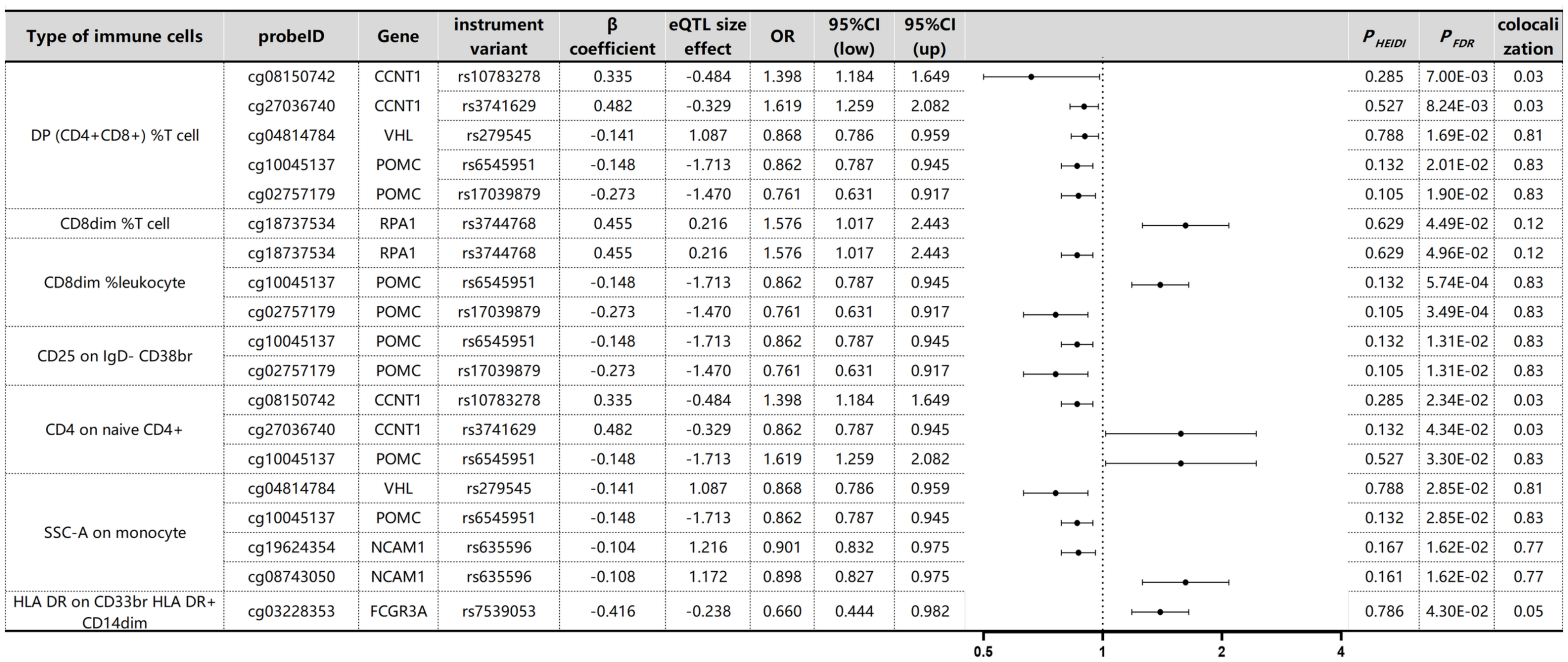
SMR and Colocalization Results for mQTLs of immune cells with causal relationships to MM. β > 0 indicates a positive correlation, and β < 0 indicates a negative correlation. Ratios are calculated according to the expected value of the causal estimate (β coefficient). Colocalisation was determined by PP.H4 between mQTLs and MM, with a PP.H4 threshold of >0.75 considered strong evidence for colocalisation.

The causal associations between immune cell DNA methylation and MM were assessed via Benjamini–Hochberg adjustment (P_FDR_ < 0.05) and HEIDI tests (Supplementary Table S7). We identified nine associated signals corresponding to eight gene loci specific to myeloma across seven immune cell types (Figure 9). Colocalisation analysis revealed that different gene variants regulating POMC exhibited varying effects on methylation levels. Yet all these effects exerted a consistent direction of influence, thereby impacting susceptibility to MM. For example, rs6545951 was associated with a 1 standard deviation decrease in POMC methylation levels, leading to a significant 24% reduction in DP (CD4^+^CD8^+^) %T-cell count (OR: 0.86, 95% CI: 0.79–0.94; P_FDR_: 2.01 × 10^−2^). Similarly, rs17039879 also decreased POMC methylation and caused a substantial decline of 34% in immune cell levels (OR: 0.76, 95% CI: 0.63–0.92; P_FDR_: 1.90 × 10^−2^). Furthermore, POMC exhibited analogous effects on other immune cells. Additionally, the VHL gene demonstrated comparable effects on DP (CD4^+^CD8^+^) %T cells and SSC-A cells on monocyte immune cells. The methylation level of NCAM1 was significantly associated with MM in HLA DR on CD33br HLA DR^+^ CD14dim immune cells. However, despite the significant association of CCNT1 with different immune cells, the colocalisation results revealed no colocalisation relationship between CCNT1 and MM.

## 4 Discussion

In this research, we conducted a systematic review and meta-analysis to evaluate the efficacy and safety of CAR-T-cell therapies across different structural domains for patients with MM/rrMM, aiming to inform clinical decision-making. We then utilized two-sample MR and SMR analysis to elucidate the causal links between distinct immune cells and MM, pinpointing key genes related to these immune cells. Our results highlight the associations between genetic factors of various immune cells and the risk of MM, offering robust evidence for uncovering the mechanisms involving genetic loci, gene expression, and methylation in the pathogenesis of this disease.

Our findings indicate that CAR-T-cell therapy is notably efficacious in this challenging patient cohort, achieving an impressive ORR of 82.2%. This includes a 45.8% CRR, a 17.3% VGPR, and an 8.8% PR. The incidence of PD was comparatively low at 7.2%. Notably, while subgroup analyses suggest that BCMA/CD19 bispecific CAR-T-cell therapy outperforms other CAR-T-cell therapies in terms of efficacy, the potential impact of the CAR-T-cell dosage and the diverse pretreatment regimens of patients should be considered.

In terms of safety, CRS is the predominant toxicity associated with CAR-T-cell therapy, resulting from systemic inflammation triggered by the immune response to CAR-T-cell proliferation. This syndrome involves a surge in cytokines, notably IL-6, IL-10, and IFN-*γ*, which can exceed the body’s regulatory capacity (51). CRS typically emerges within days and resolves within 2–3 weeks, with severity and duration influenced by patient-specific factors, CAR-T-cell characteristics, and therapeutic strategies (52). Clinical presentations vary from mild symptoms such as myalgia, rash, and fever to severe complications, including shock, coagulopathy, capillary leak syndrome, and organ dysfunction. In exceptional cases, CRS can mimic macrophage activation syndrome (MAS) (51, 53). Our analysis revealed that a substantial majority of patients (85.8%) experienced CRS of any grade, with only 6.3% exhibiting grade 3 or higher. These data highlight the critical need for vigilant CRS monitoring and management.

Our meta-analysis revealed significant infection rates among MM patients after CAR-T-cell therapy, with 49.1% experiencing any-grade infections and 18.2% facing severe (grade 3 or higher) infections. These infections, possibly due to therapy, prior treatments, or the disease itself, can emerge at varying times after treatment. Associated conditions such as hypogammaglobulinaemia, cytopenias, and T-cell exhaustion increase the risk of infection (54), potentially leading to extended hospital stays, reduced quality of life, and treatment interruption. This highlights the critical need for effective infection management to ensure the best treatment outcomes. Research indicates that fractionated CAR-T-cell therapy could mitigate toxicity without affecting treatment efficacy (55). Notably, an approach involving an initial infusion and a subsequent booster 100 days later has yielded minimal toxicity, including no ICANS, late-onset neurotoxicity, or grade 3+ CRS. This aligns with findings from the use of dose fractionation in ARI-0001 CAR-T-19 cell therapy for acute lymphoblastic leukaemia, which also showed reduced severe toxicity (56). These results advocate further exploration of dose fractionation to consolidate its advantages and enhance treatment strategies.

Current research suggests that CAR-T-cell therapy holds promise for sustained control and potentially a cure for MM. However, the high efficacy of CAR-T-cell therapy contrasts with the subdued response of MM patients to immune checkpoint inhibitors (57). Thus, a nuanced comprehension of the MM immune microenvironment is essential for deciphering the mechanisms behind the varying outcomes of immunotherapeutic approaches. In this context, we utilized two-sample MR and SMR analysis to clarify the causal relationships between specific immune cells and MM, pinpointing key genes related to these cells.

VDR, an intranuclear vitamin D receptor, binds to 1,25 (OH)_2_D3 to regulate growth (58). Various polymorphisms, including ApaI, BsmI, and FokI, have been identified in different introns and exons of the VDR gene (59). Previous studies have demonstrated their potential associations with the risk of several cancers, such as colorectal cancer (60), breast cancer (61), prostate cancer (62), and MM (63). Our study suggested that VDR is a susceptibility gene for MM associated with various immune cell types. Additionally, evidence indicates that vitamin D could have anticancer properties, potentially by directly influencing tumor cell differentiation, proliferation, and apoptosis and indirectly modulating immune cells within the tumor microenvironment (64). Therefore, we propose that VDR could serve as a promising therapeutic target for MM.

FANCM, together with other Fanconi anemia (FA) proteins, detects interstrand crosslink (ICL) damage and associates with chromatin, serving as a docking site for the core FA complex (65). FANCD2 is upregulated in patients diagnosed with MM and is associated with an unfavorable prognosis, particularly in those presenting high-risk diseases (66). Our research reinforces the notion that FANCD2 acts as a risk factor for MM, highlighting its potential as a therapeutic target. Our findings indicate that wagonin can suppress angiogenesis driven by the c-Myc/VHL/HIF-1α pathway in MM, suggesting that VHL may serve as a promising target for treatment (67). While the precise function of POMC in MM has yet to be elucidated and warrants further experimental research, our study revealed a notable correlation between the methylation of the VHL and POMC genes and the onset of MM. Although a significant link was initially detected between VDR gene methylation and disease through SMR analysis, this correlation did not hold after correction for multiple comparisons. Additionally, our results revealed a connection between NCAM1 gene methylation and MM.

The key strengths of this study are its systematic review and meta-analysis, which is informed by the latest evidence, and thoroughly evaluates the efficacy and safety of CAR-T-cell therapy in rrMM patients, aiding clinical decision-making. Additionally, our MR analysis elucidates the causal links between immune cells and MM. Leveraging a large sample size and GWAS data, we ensured robust statistical power for establishing these causal links and definitively estimating MM-related outcomes. We applied a variety of analytical methods, including traditional two-sample MR, SMR, and sensitivity analyses with four additional MR techniques and collocation analysis, to bolster the findings’ reliability. By focusing on individuals of European ancestry, we reduced biases associated with genetic diversity.

The interpretation of our systematic review and meta-analysis outcomes has several limitations. Notably, the heterogeneity among the included studies is a key consideration, with significant variance persisting even after subgroup analysis. Furthermore, conclusions regarding specific bispecific CAR-T-cell therapies are premised on limited clinical trials and necessitate confirmation through extensive, long-term studies. In the MR component of our study, we faced limitations, including the lack of associations within the eQTL and mQTL datasets for gene expression or mutations on the X or Y chromosomes. While univariate MR provides an overall effect estimate, we did not leverage multivariate MR to dissect the individual causal impacts of various immune cells on MM despite its potential to offer a more nuanced understanding by assessing multiple exposures concurrently. Finally, we opted for the Bonferroni–Holm correction over the stricter Bonferroni method to balance type I error control without overly penalizing true positives.

## 5 Conclusion

This study provides the first evidence supporting the efficacy of CAR-T-cell therapy in rrMM patients, with a systematic review and meta-analysis showing an ORR of 82.2%. The therapy also demonstrated a favorable safety profile, with only 6.3% experiencing ≥3 grade CRS and 0.9% experiencing ≥3 grade neurotoxicity. Subgroup analysis suggested that BCMA/CD19 bispecific CAR-T-cell therapy outperforms other approaches in terms of the ORR, although this requires confirmation through extensive clinical trials. Additionally, MR analysis revealed potential causal links between specific immune cells and MM, identifying immune cells significantly associated with the disease and genes such as VDR and VHL significantly linked to these cells. Overall, this study uses meta-analysis to inform clinical decisions and MR to enhance understanding of the MM immune microenvironment, providing valuable insights into its pathophysiology.

## Data Availability

The original contributions presented in the study are included in the article/Supplementary material, further inquiries can be directed to the corresponding author/s.
