# Supplementary material for: Unveiling causal immune cell–gene associations in multiple myeloma: insights from systematic reviews and Mendelian randomization analyses
Source: Front Med (Lausanne). 2025 Jan 22;12:1456732. doi: 10.3389/fmed.2025.1456732 (PMC11794323; doi:10.3389/fmed.2025.1456732)
Supplement: Supplementary file 1 [file Data_Sheet_1.docx]

**Supplementary material**

**Supplementary methods**

1. **Meta analysis**

**1.1 Data Extraction**

Data were collected on the following aspects: (1) Study characteristics: first author, publication year, study design, production name, registration number, study phase, country, sample size, bridging therapy, lymphodepletion regimen, CAR-T cell dosage, median follow-up duration, target antigen, source of T cells, gene transfer method, costimulatory domain, species of scFv (single-chain variable fragment), and CAR-T cell manufacturing process; (2) Patient demographics: age, gender, ethnicity, median time since diagnosis, ECOG PS score, ISS staging, extramedullary disease, and high-risk cytogenetic features; (3) Outcome assessments.

**1.2 Risk of Bias**

Two independent reviewers utilized the Methodological Index for Non-Randomized Studies (MINORS) to assess the quality of non-randomized studies. The assessment criteria encompass eight elements: clearly stated aims, inclusion of consecutive patients, prospective data collection, endpoints appropriate to the study's objectives, unbiased assessment of the study endpoints, follow-up period appropriate to the study's objectives, less than 5% loss to follow-up, and prospective calculation of the study size. Each study could achieve a maximum of 16 points. Each criterion was scored as 0 (not reported), 1 (reported but inadequate), or 2 (reported and adequate) to evaluate the study's quality. Furthermore, the two independent reviewers measured agreement using the weighted Cohen's kappa coefficient (k). A kappa value of 0.0 to 0.20 indicates poor agreement, 0.21 to 0.40 suggests fair agreement, 0.41 to 0.60 denotes moderate agreement, 0.61 to 0.80 reflects strong agreement, and 0.81 to 1.0 indicates very strong agreement.

**1.3 Data Synthesis and Statistical Analysis**

Statistical analyses were conducted using R studio software (Meta package) to assess studies evaluating the impact of CAR-T cell therapy on patients with relapsed/refractory multiple myeloma (rrMM).

The following outcomes were selected for inclusion in the meta-analysis: Overall Response Rate (ORR), Complete Response Rate (CRR), Very Good Partial Response (vgPR), Partial Response (PR), Progressive Disease (PD), Cytokine Release Syndrome (CRS), neurotoxicity, hematological adverse events (neutropenia, leukopenia, anemia, thrombocytopenia, lymphopenia), infections, and all-cause mortality.

Effects were measured using proportions, with results expressed as 95% confidence intervals (95% CIs). Heterogeneity among studies was assessed using Cochran's Q test and the I2 statistic, with significant heterogeneity considered present when p < 0.10 and/or I2 > 40%. In such cases, a random-effects model was employed. Additionally, subgroup analyses were conducted for different structural domains of CAR-T cell therapies to mitigate potential heterogeneity. A p-value of < 0.05 was considered statistically significant. Forest plots were used to illustrate the results of the analyses, and funnel plots were examined to detect the presence of publication bias. Furthermore, Egger's test and Begg's test were utilized to quantitatively assess publication bias, with p-values greater than 0.05 indicating no significant publication bias.

1. **Mendelian randomization**
   1. **GWAS data sources**

To elucidate the causal relationship between immune markers and multiple myeloma, we selected the most extensive GWAS to date for the immunophenotyping of peripheral blood. This dataset encompassed 118 absolute cell (AC) counts, 389 median fluorescence intensities (MFIs) indicative of surface antigen levels, 32 morphological parameters [MP, including forward scatter (FSC) and side scatter (SSC), which correlate with cell volume and intracellular complexity as well as surface texture, respectively], and 192 relative cell (RC) counts. The GWAS summary statistics for these 731 immune traits are publicly accessible in the GWAS Catalog, with accession numbers ranging from GCST0001391 to GCST0002121. This GWAS analysis was conducted utilizing 3,757 Sardinian samples, comprising 57% women, to interrogate approximately 22 million single nucleotide polymorphisms (SNPs). Genotyping was performed using high-density arrays, and the analysis was adjusted for sex, age, and the square of age to account for age-related variability. Subsequently, the SNPs were imputed utilizing a reference panel based on Sardinian sequences, ensuring greater accuracy in the genetic inference.

The multiple myeloma GWAS data utilized in this study were primarily derived from the FinnGen project, an extensive collaborative effort involving the collection and analysis of genetic data from over 500,000 participants within the Finnish Biobank. This robust dataset provides a comprehensive resource for genetic research, enabling the exploration of genotype-phenotype correlations within the Finnish founder population. The FinnGen project, with its substantial sample size and focus on the Finnish population, offers a unique opportunity to identify genetic variants associated with multiple myeloma and other diseases. For further details on accessing FinnGen data, including summary statistics and GWAS results, researchers can refer to the project's website and follow the provided guidelines.

**2.2 Statistical analysis**

Mendelian randomization requires meeting three core assumptions. Assumption 1: the genetic instruments are robustly associated with the exposure. Assumption 2: the genetic instruments are not associated with any major confounders. Assumption 3: the genetic instruments affect the outcome only through exposure.

Data were standardized by excluding SNPs with ambiguous alleles and palindromic SNPs. The primary MR analysis was conducted using the multiplicative random-effects inverse variance weighting (IVW) method, which yields accurate estimates assuming all SNPs are valid instruments. Fixed-effects meta-analyses were employed to combine estimates from different sources, while heterogeneity between associations was assessed using I^2^ statistics and Cochran's Q values for SNP estimates in each association. I^2^ statistics were calculated to evaluate heterogeneity between outcomes from different sources, with I^2^ values <25%, 25-75%, and >75% indicating low, moderate, and high heterogeneity respectively. Sensitivity analyses included weighted median, MR-Egger, and MR-pleiotropy residuals and outliers (MR-PRESSO) analyses to detect potential unbalanced pleiotropy (horizontal pleiotropy)

**2.3 Selection of instrumental variables**

Consistent with the data utilized in recent research, we employed a uniform set of criteria to select multiple instrumental variables (IVs) for each immune trait in our Mendelian Randomization (MR) analysis. The threshold for identifying significant IVs was liberally set at 1.00E−5. Subsequently, we employed PLINK software (version 1.90) to refine this set of IVs by eliminating those in high linkage disequilibrium (LD), as determined by the 1000 Genomes Projects (28). The LD threshold was conservatively set, with an r2 threshold of 0.1 and a physical distance criterion of within 500 kilobases. Furthermore, for the assessment of multiple myeloma (MM) traits, we implemented a more stringent threshold, defining significance at the 5.00E−8 level and an r2 threshold of 0.01.

F statistics were computed to assess the potency of genetic instrumental variables (IVs). Only single nucleotide polymorphisms (SNPs) with an F statistic exceeding 10 were regarded as non-weak genetic instruments. To examine and rectify the potential horizontal pleiotropy of the selected instrumental variables (IVs), the Mendelian randomization-Egger (MR-Egger) regression and the Mendelian randomization pleiotropy residual sum and outlier (MR-PRESSO) method were employed. The MR-Egger intercept test served as an indicator for detecting directional pleiotropy. Moreover, when the p-value was less than 0.05, suggesting the existence of directional pleiotropy, the MR-PRESSO method was capable of correcting the outliers within the IVs and furnishing an estimate comparable to that obtained from the inverse variance weighted (IVW) method following the removal of these outliers. To address multiple testing issues, the Benjamini-Hochberg correction for false-discovery rates was applied; only associations with a Benjamini-Hochberg corrected p-value <0.01 were considered statistically significant.

**2.4 sensitivity analysis**

To determine the causal influence of immune traits on the susceptibility to multiple myeloma, we employed a suite of robust Mendelian Randomization (MR) analytical approaches underpinned by diverse assumptions. These methodologies were utilized to infer causal relationships between variables and phenotypic outcomes and included the fixed-effect inverse variance weighting (IVW) as the principal analysis, complemented by sensitivity analyses using the weighted median-based MR-Egger, and the MR pleiotropy residual sum and outlier (MR-PRESSO) tests. The IVW method was executed as the primary analysis, with subsequent comprehensive sensitivity analyses conducted to discount potential breaches of MR presuppositions, specifically heterogeneity and pleiotropy. Cochran's Q statistic was applied to assess heterogeneity, where a p-value threshold of <0.05 was established to denote significant heterogeneity. In instances where the null hypothesis was refuted, indicating potential heterogeneity among the IVs, the random-effect IVW was favored over the fixed-effect IVW. The MR-Egger regression intercept and the MR-PRESSO global test were instrumental in identifying pleiotropic effects. Concluding this sequence, the MR-PRESSO outlier test was implemented to scrutinize the potential for bias in MR findings attributable to pleiotropy.

### 2.5 Summary-data-based Mendelian randomization analyses

### Summary-based Mendelian randomization (SMR) was utilized for in-depth analysis, serving as an extension of the traditional MR approach to quantify pleiotropic associations between genetically regulated traits—such as gene expression, DNA methylation, or protein levels—and complex disease phenotypes. Employing a two-step least-squares (2SLS) method, SMR estimates the causal effect of an exposure on an outcome using genetic variants robustly linked to the exposure as instrumental variables. The analysis was conducted using the SMR software for Windows, version 1.0.3, with default command-line parameters as per the provided resource (https://yanglab.westlake.edu.cn/software/smr/#Overview).

### Leveraging GWAS summary statistics and expression quantitative trait loci (eQTLs), particularly focusing on multiple variants within the cis-eQTL domain, SMR facilitated the identification of genes meriting further functional exploration. Furthermore, SMR incorporated the HEIDI test to assess instrumental-dependency heterogeneity, thereby discerning whether observed associations indicated vertical pleiotropy or were confounded by linkage disequilibrium (LD) and causal variation. LD estimates referenced data from the European Ancestry Genome Consortium. Associations significant at the P<0.05 level on the HEIDI test were considered likely due to LD, suggesting that the same genetic variant was influencing both the exposure and outcome independently. Consequently, such associations were prudently excluded from the analysis to maintain the integrity of the pleiotropy assessment.

### 2.6 Colocalization Analysis

### The colocation method is a statistical approach designed to assess whether two traits share causal genetic variation within a specific genomic region. The colocation analysis was predicated on four hypotheses: H0: no trait possesses causal genetic variation; H1: only Trait 1 is underpinned by causal genetic variation; H2: only Trait 2 is influenced by causal genetic variation; H3: both traits are influenced by causal genetic variation, but not the same; and H4: both traits share identical causal genetic variations.

### To bolster the reliability of our results, we performed an additional Bayesian analysis using the 'coloc' R package (version 5.1.0), available at <https://chr1swallace.github.io/coloc/>, to estimate the posterior probability of shared genetic effects. For each significant SNP in the GWAS dataset for multiple myeloma, we extracted all SNPs within a 100-kb flanking region for colocation analysis, focusing on the posterior probability of H4 (PP.H4). A PP.H4 value exceeding 0.75 was established as a stringent threshold, providing compelling evidence for the colocation of GWAS and QTL associations.

**
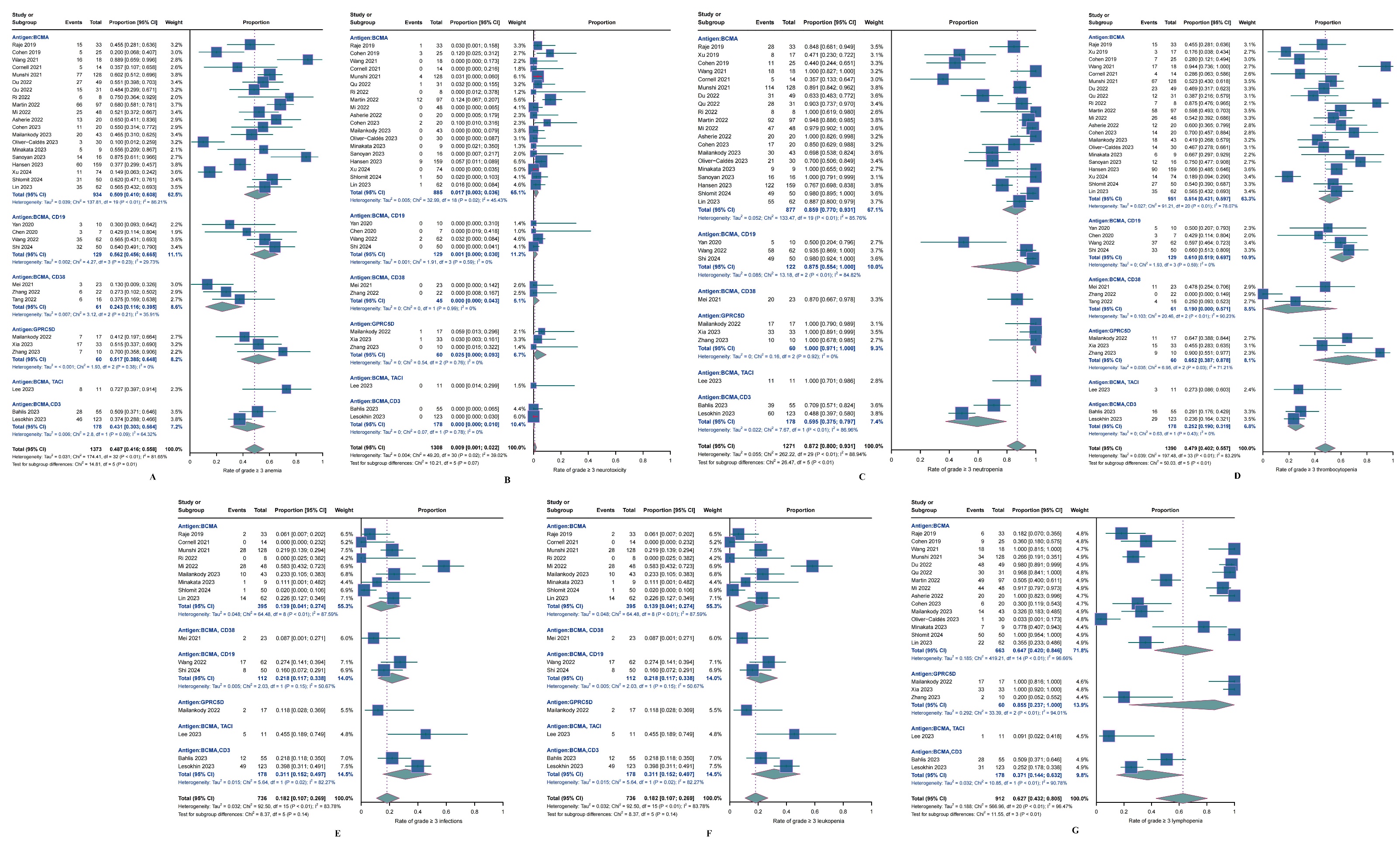
**

**Figure S1. Meta-Analysis of safety outcomes grade ≥ 3 in MM Patients Treated with CAR T-Cell Therapy. (A) grade ≥ 3 anemia; (B) grade ≥ 3 neurotoxicity; (C) grade ≥ 3 neutropenia; (D) grade ≥ 3 thrombocytopenia; (E) grade ≥ 3 infection; (F) grade ≥ 3 leukopenia; (G) grade ≥ 3 lymphopenia.**

**
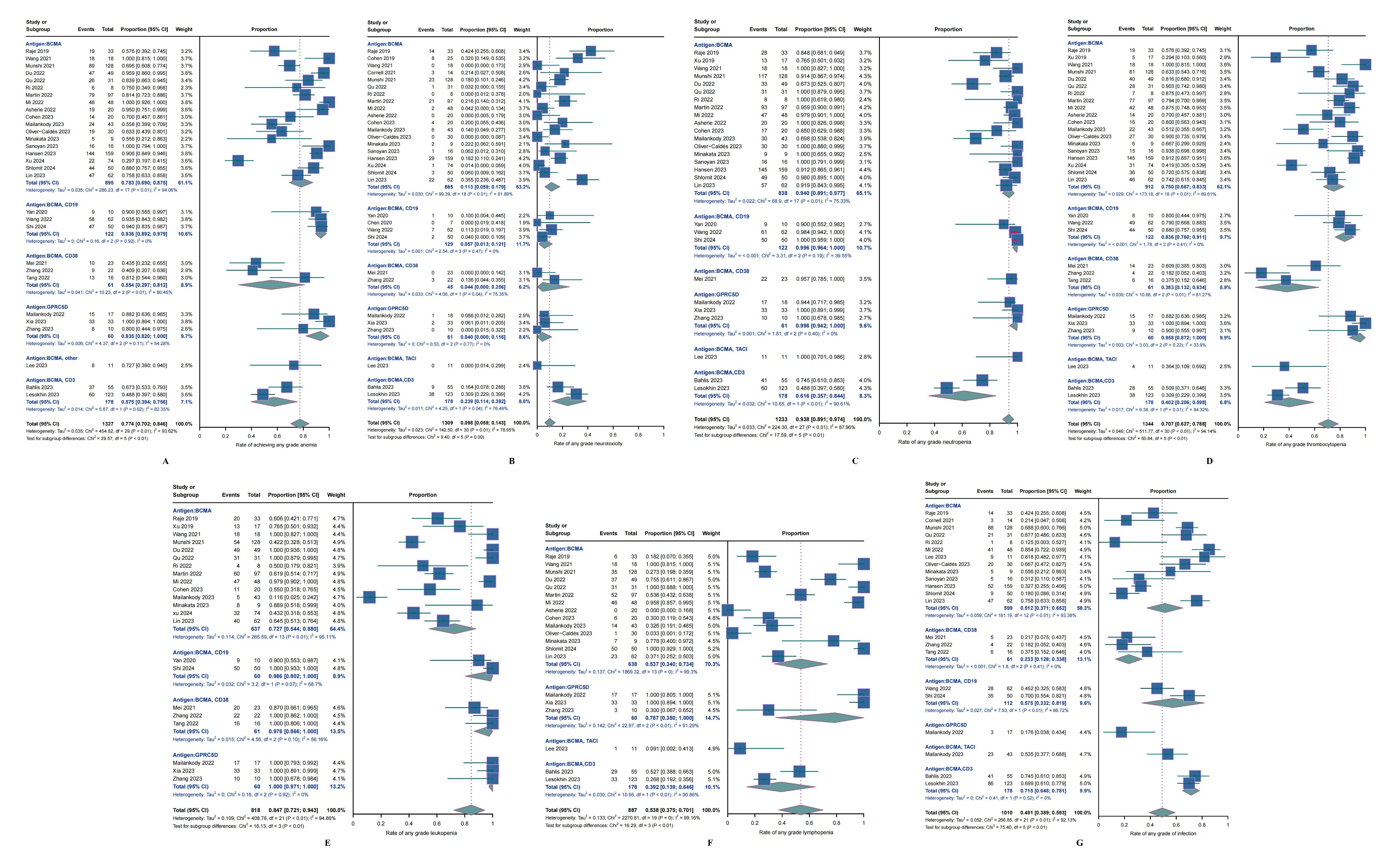
**

**Figure S2. Meta-Analysis of safety outcomes at at any grade in MM Patients Treated with CAR T-Cell Therapy. (A) any grade anemia; (B) any grade neurotoxicity; (C) any grade neutropenia; (D) any grade thrombocytopenia; (E) any grade leukopenia; (F) any grade lymphopenia; (G) any grade infection.**

| **Supplementary Table 1. Information of QTL and GWAS datasets.** | | | | | | |
| --- | --- | --- | --- | --- | --- | --- |
| **Type of dataset** | **Data subtype** | **Resource** | **Sample size** | **Population** | **Reference** | **Download Site** |
| eQTL | cis-eQTL | V8 release of the GTEx eQTL | 838 | European | GTEx Consortium. Science 2021. PMID:32913098. The GTEx Consortium atlas of genetic regulatory effects across human tissues. | https://yanglab.westlake.edu.cn/software/smr/#eQTLsummarydata |
| mQTL | cis-mQTL | McRae et al. mQTL summary data | 1980 | European | Wu Y, et al. Nat Commun. 2018. PMID: 29500431. Integrative analysis of omics summary data reveals putative mechanisms underlying complex traits. | https://yanglab.westlake.edu.cn/data/SMR/LBC_BSGS_meta_lite.tar.gz |
| GWAS summary | 731 immune cells | GCST0001391 to GCST0002121 | case: 32,957, control: 434,296 | European | Valeria O, et al. Nat Genet. 2020. PMID: [32929287](https://www.ncbi.nlm.nih.gov/pubmed/32929287). [Complex genetic signatures in immune cells underlie autoimmunity and inform therapy](https://www.pmop.cn/pubmed/32929287) | https://www.ebi.ac.uk/gwas/studies/GGCST0001391-CST0002121 |
| GWAS summary | multiple myeloma | FinnGen | case: 623. control: 345105 | European | Kurki,et al. Nature. 2023 PMID:36653562 FinnGen provides genetic insights from a well-phenotyped isolated population. | gs://finngen-public-data-r9/summary_stats/finngen_R10_C3_MULT_MYELOMA_EXALLC.gz |

| **Supplementary material Table 2. Characteristics of the included studies for meta analysis.** | | | | | | | | | | | | | | | | | | |
| --- | --- | --- | --- | --- | --- | --- | --- | --- | --- | --- | --- | --- | --- | --- | --- | --- | --- | --- |
| **first author and Publication year** | **Country** | **Study design** | **CAR-T Cell Dose(n)** | **Extramedullary Disease, Number(%)** | **High-Risk Cytogenetic Profile, Number (%)** | **Median Follow-Up (Range), (d,day; m, month)** | **Sample size** | **Production name** | **Registration number** | **Study phase** | **CAR-T cell generation** | **Target antigen** | **scFv species** | **Age, median (range), y** | **Male/**  **female** | **Median time since diagnos(range)(m, month; y, year)** | **ECOG PS score, number (%)** | **ISS staging, number (%)** |
| Raje 2019 | USA | Prospective study (clinical trial) | 50 × 106 cells(3), 150 × 106 cells(6),  450 × 106 cells(9), 800 × 106 cells(3)  Dose expansion—150 × 106 cells(2), 450 × 106 cells(10) | 9 (27) | 15 (46)  del(17p), t(4;14), t(14;16) | 11(6–23)(m) | 33 | Idecabtagene vicleucel/ide- cel/bb2121, (CRB-401) | NCT02658929 | Phase 1 | Second-generation | BCMA | Murine | 60 (37-75) | 12/21 | 5(1-36)(y) | 0 – 10 (30)  1 – 21 (64)  2 – 2 (6) | I – 7 (21)  II – 14 (42)  III – 8 (24)  Unknown – 4 (12) |
| Xu 2019 | China | Prospective study (clinical trial) | 0.21–1.52 × 106 CAR-T cells/Kg | NR | 6(35)  t(4;14), del(17p) | 417(12–535)(d) | 17 | LCAR-B38M | NCT03090659 ChiCTR-ONH-17012285 | Phase 1 | Second-generation | BCMA | Llama | 55 (35-73) | 11/6 | NR | NR | NR |
| Cohen 2019 | USA | Prospective study (clinical trial) | Cohort 1—100–500 × 106 cells  Cohort 2—10–50 × 106 cells  Cohort 3—100–500 × 106 cells | 7 (28) | 24 (96)  del(17p), t(14;16), t(4;14) | NR | 25 | CART-BCMA | NCT02546167 | Phase 1 | Second-generation | BCMA | Human | 58 (44-75) | 17/18 | 4.6(1.8-14.5)(y) | NR | NR |
| Yan 2020 | China | Prospective study (clinical trial) | Anti-BCMA—2.5–6.8 × 107 cells/Kg  Anti-CD19—1 × 107 cells/Kg | NR | 5 (50)  t(4;14), 1q21 amp | 20(m) | 10 | CD19 & BCMA CAR-T | NCT03196414 | Phase 1 | Third-generation | BCMA, CD19 | Humanized | 56 (43-69) | 7/3 | NR | NR | NR |
| Chen 2020 | China | Prospective study (clinical trial) | Anti-BCMA—1–2 × 106 cells/Kg  Anti-CD19—1 × 106 cells/Kg | 1 (14) | 2 (28)  del(17p), t(4;14) or t(14;16) | 433d (230–742) | 7 | CD19 & BCMA CAR-T cells | ChiCTROIC-17011272 | Phase 2 | Third-generation | BCMA, CD19 | Anti-CD19 - Humanized Anti-BCMA - Murine | 49 (41-55) | 7/0 | 23.5(7.9-34.2)(m) | NR | I – 2 (29)  II – 2 (29)  III – 3 (43) |
| Wang 2021 | China | Prospective study (clinical trial) | 1 × 106 cells/Kg (9), 3 × 106 cells/Kg (6), 6 × 106 cells(3) | 5 (27.8) | 7 (38.9) | 394(d) | 18 | CT103A | ChiCTR1800018137 | Phase 1 | Second-generation | BCMA | Human | 53.5 (38-66) | 10/8 | 31.9(8.80-94.3)(m) | 0 – 3 (17)  1 – 15 (83) | I – 10 (56) II – 8 (44) III – 0 |
| Cornell 2021 | USA | Prospective study (clinical trial) | 3–100 × 107 cells | NR | 2 (12) | 12(8.7–14)(m) | 14 | KITE-585 CAR | NCT03318861 | Phase 1 | Second-generation | BCMA | Human | 56 (47-71) | 10/7 | 61(27-134)(m) | 1 – 7 (41) | I/II – 7 (41)  III – 4 (24)  Unknown – 6 (35) |
| Mei 2021 | China | Prospective study (clinical trial) | 0.5—4.0 × 106 cells/Kg | 9 (39) | 17 (74)  del(17p), del(13q), t(4;14), t(11;14) and t(14;16) | 9(0.5–18.5)(m) | 23 | BM38 CAR | ChiCTR1800018143 | Phase 1 | Second-generation | BCMA, CD38 | Anti-BCMA - Human Anti-CD38 - Humanized | 59 (49-72) | 11/12 | 2.9(0.4-13.4)(y) | 0-2 – 19 (83) 3-4 – 4 (17) | I – 6 (26)  II – 6 (26)  III – 11 (48) |
| Munshi 2021 | USA, Canada,  Belgium, France,  Germany, Italy,  Japan, Spain | Prospective study (clinical trial) | 150 × 106 cells(n = 4), 300 × 106 cells(n = 70), 450 × 106 cells(n = 54) | 50 (39) | 45 (35)  del(17p), t(14;16), t(4;14) | 13.3(0.20–21.2)(m) | 128 | idecabtagene vicleucel/ ide- cel/ bb2121 (KarMMa) | NCT03361748 | Phase 2 | Second-generation | BCMA | Murine | 61 (33-78) | 76/52 | 6(1-18)(y) | 0 – 57 (45)  1 – 68 (53)  2 – 3 (2) | I – 14 (11)  II – 90 (70)  III – 21 (16)  Unknown – 3 (2) |
| Zhang 2022 | China | Prospective study (clinical trial) | Anti-BCMA—2.0 × 106 cells/Kg  Anti-CD38—2.0 × 106 cells/Kg | 3 (13.6) | 19 (86.4)  del(17p), t(14;16), t(4;14 | 24(0.5–33)(m) | 22 | BCMA & CD38 CAR-T cells | ChiCTR1800017051 | Phase 2 | Second-generation | BCMA, CD38 | Anti-BCMA - Human Anti-CD38 - Humanized | 56 (47-68) | 9/13 | NR | NR | I – 0 (0)  II – 17 (77)  III – 5 (23) |
| Du 2022 | China | Prospective study (clinical trial) | 9 × 106 cells/Kg | 11 (22.45) | 21 (42.86)  del(17p), t(14;16), t(4;14) | 14(1–42.5)(m) | 49 | BCMA CAR/ HDS269B | NCT03093168 | Phase 1/2 | Second-generation | BCMA | Murine | 57 (37-75) | 26/23 | 2.7(0.3-12.6)(y) | 0-1 – 17 (35)  2 – 12 (23)  3 – 14 (29)  4 – 6 (13) | I – 6 (12)  II – 20 (41)  III – 13 (27)  Unknown – 10 (20) |
| Wang 2022 | China | Prospective study (clinical trial) | 1 × 106 cells/Kg | 15 (24) | 18 (29)  del(17p), t(14;16), t(4;14) | 21.3(m) | 62 | CD19 & BCMA CAR-T cells | ChiCTROIC-17011272 | Phase 2 | Third-generation | BCMA, CD19 | Anti-CD19 - Humanized Anti-BCMA - Murine | 58 (30-69) | 34/28 | 30 (8-167)(m) | NR | I – 8 (13)  II – 24 (39)  III – 30 (48) |
| Mailankody 2022 | USA | Prospective study (clinical trial) | 25 × 106 cells(3), 50 × 106 cells(3), 150 × 106 cells(6), 450 × 106 cells(5) | 8 (47) | 13 (76)  del(17p), t(14;16), t(4;14) and 1q gain | 10.1(m) | 17 | GPRC5D CAR-T cells/ MCARH109 | NCT04555551 | Phase 1 | Second-generation | GPRC5D | Huma | 60 (38-76) | 13/4 | NR | NR | NR |
| Qu 2022 | China | Prospective study (clinical trial) | 1.0 × 106 cells(4), 3.0 × 106 cells(13), 4.5–6.0 × 106 cells(14) | 3 (9.7) | 15 (48)  del(17p), p53 mutation, t(14;16), t(4;14), t(14;20) and 1q gain | 9.4(1.9–24.2)(m) | 31 | C-CAR088 | NCT03815383  NCT03751293  NCT04295018  NCT04322292 | Phase 1 | Second-generation | BCMA | Huma | 61 (45-74) | 17/14 | NR | 0 – 19 (61) 1 – 12 (39) | I – 4 (13)  II – 21 (68)  III – 5 (16)  Unknown – 1 (3) |
| Tang 2022 | China | Prospective study (clinical trial) | 0.5–10.0 × 106 cells/Kg | 8 (50) | 11 (68.8)  Including 1q21, del17p | 11.5(6.0–26.0)(m) | 16 | BCMA & CD38 CAR-T cells | ChiCTR1900026286 | Phase 1 | Second-generation | BCMA, CD38 | NR | 58.5 (48-78) | NR | 28.5(4-72)(m) | NR | I – 2 (12.5)  II – 1 (6.25)  III – 13 (81.3) |
| Ri 2022 | Japan | Prospective study (clinical trial) | 0.41–0.72 × 106 cells/Kg | 3 (33.3) | 5 (55.6)  del(17p), t(14;16), t(4;14) | 8.5(m) | 9 | Ciltacabtagene autoleucel/cilta-cel/ JNJ- 68284528 (CARTITUDE-1) | NCT03548207 | Phase 2 | Second-generation | BCMA | Llama | 57 (45-71) | 5/4 | 5.41(3.8-11.3)(y) | 0 – 7 (78)  1 – 1 (11)  2 – 1 (11) | I – 5 (56)  II – 3 (33)  III – 1 (11) |
| Martin 2022 | USA | Prospective study (clinical trial) | 0.51–0.95 × 106 cells/Kg | 13 (13) | 23 (24)  del(17p), t(14;16), t(4;14) | 27.7(m) | 97 | Ciltacabtagene autoleucel/cilta-cel/ JNJ- 68284528 (CARTITUDE-1) | NCT03548207 | Phase 1b/2 | Second-generation | BCMA | Llama | 61 (56-68) | 57/40 | 5.9(4.4-8.4)(y) | 0 – 39 (40)  1 – 54 (56)  2 – 4 (4) | I – 61 (63)  II – 22 (23)  III – 14 (14) |
| Mi 2022 | China | Prospective study (clinical trial) | 0.42–0.84 × 106 cells/Kg | 5 (10.4) | 21 (43.8)  del(17p), t(14;16), t(4;14) | 18(0.20–28.0)(m) | 48 | Ciltacabtagene autoleucel/cilta-cel/ JNJ- 68284528 (CARTIFAN-1) | NCTO3758417 | Phase 2 | Second-generation | BCMA | Llama | 61 (30-72) | 32/16 | 3.7(1.4-10.2)(y) | 0 – 22 (46) 1 – 26 (54) | I – 21 (44) II – 18 (38) III – 9 (18) |
| Asherie 2022 | Israel | Prospective study (clinical trial) | 150 × 106 cells(6), 450 × 106 cells(7), 800 × 106 cells(7) | 6 (30) | 10 (50)  del(17p), t(14;16), t(4;14) | 136(d) | 20 | HBI0101 | NCT04720313 | Phase 1 | Second-generation | BCMA | Murine | 62 (44-75) | 8/12 | 55(8-241)(m) | 0 – 7 (35)  1 – 4 (20)  2 – 9 (45) | I – 1 (5)  II – 11 (55)  III – 2 (10) |
| Cohen 2023 | USA, Belgium,  France, Germany,  Israel, Netherlands,  Saudi Arabia, Spain | Prospective study (clinical trial) | 0.21–1.11 × 106 cells/Kg | 5 (25) | 3 (15), all del17p | 11.3 (0.60–16.0)(m) | 20 | Ciltacabtagene  autoleucel/cilta-cel/ JNJ-  68284528 (CARTITUDE-2,  cohort C) | NCT04133636 | Phase 2 | Second-generation | BCMA | Llama | 62.5 (44-81) | 12/8 | 6.3(2.5-16.3)(y) | 0 – 8 (40) 1– 12 (60) | I– 8 (40) II – 4 (20) III – 8 (40) |
| Xia 2023 | China | Prospective study (clinical trial) | 2 × 106 cells/Kg | 11 (33) | 13 (39)  del(17p), t(14;16), t(4;14) and amp(1q) | 10.2 (3.8-NE)(m) | 33 | GPRC5D CAR-T cells/ MCARH109 | ChiCTR2100048888 | Phase 2 | Second-generation | GPRC5D | Human | 58 (39-70) | 18/15 | 31.5(4.8-96.0)(m) | NR | I – 6 (18)  II – 15 (45)  III – 12 (36) |
| Mailankody 2023 | USA | Prospective study (clinical trial) | 40 × 106 cells(3), 160 × 106 cells(7), 320 × 106 cells(27), 480 × 106 cells(6) | 9 (20.9) | 16 (37.2)  del(17p), t(14;16), t(4;14) | 10.2 (3.8-NE)(m) | 43 | ALLO-715 (UNIVERSAL, cohort A) | NCT04093596 | Phase 1 | Second-generation | BCMA | Human | 64 (46-77) | 27/16 | 4.9(0.9-26.4)(y) | 0 – 21 (49)  1 – 22 (51) | I – 12 (28)  II – 22 (51)  III – 8 (19)  Unknown – 1 (2) |
| Lee 2023 | UK, Netherlands | Prospective study (clinical trial) | 15–900 × 106 cells | 3 (27.3) | 4 (36.4)  t(4;14), t(14;20) and t(14;16), del(17p), 1q gain, 1p loss | NR | 11 | APRIL CAR-T cells (AUTO2) | NCT03287804 | Phase 1 | Third-generation | BCMA, TACI | NR | 61 (45-69) | 8/3 | 6(1-11)(y) | NR | I – 6 (55)  II – 1 (9)  III – 3 (27)  Unknown – 1 (9) |
| Oliver-Caldés 2023 | Spain | Prospective study (clinical trial) | 0.3–3 × 106 cells/Kg | 6 (20) | 10 (33)  TP53 alterations, t(14;16), t(4;14) | 18(15–20)(m) | 30 | ARI0002h (CARTBCMA- HBC-01) | NCT04309981 | Phase 1/2 | Second-generation | BCMA | Humanized | 61 (53-65) | 18/12 | 4.7(3.7-9.1)(y) | 0 – 18 (62)  1 – 9 (31)  2 – 2 (7) | I – 5 (20)  II – 8 (32)  III – 12(48) |
| Zhang 2023 | China | Prospective study (clinical trial) | 1 × 106 cells(3), 3 × 106 cells(4), 6 × 106 cells(3) | 4 (40) | 6 (60)  del(17p), t(14;16), t(4;14) | 238 (182–307)(d) | 10 | GPRC5D CAR-T cells/ OriCAR-017 (POLARIS) | NCT05016778 | Phase 1 | Second-generation | GPRC5D | Llama | 64 (58-68) | 5/5 | 39(25-78)(m) | 0 – 1 (10)  1 – 3 (30)  2 – 6 (60) | I – 2 (20)  II – 5 (50)  III – 3 (30) |
| Minakata 2023 | Japan | Prospective study (clinical trial) | 450 × 106 cells | 5 (56) | 2 (22)  del(17p), t(4;14) | 12.9 (3.30–17.8)(m) | 9 | idecabtagene vicleucel/ ide- cel/ bb2121, (KarMMa) | NCT03361748 | Phase 2 | Second-generation | BCMA | Murine | 54 (38-73) | 7/2 | 3.6(1.0-7.9)(y) | 0 – 6 (67)  1 – 3 (33)  2 – 0 | I – 2 (22)  II – 4 (44)  III – 2 (22)  Unknown – 1 (11) |
| Sanoyan 2023 | Switzerland | Retrospective study (real- world data) | 450 × 106 cells | 5 (31) | 6 (38)  del(17p), t(14;16), t(4;14) | 5.7 (0.6–9.0)(m) | 16 | idecabtagene/ide-cel/bb2121 | NA | NA | Second-generation | BCMA | Murine | 69 (57-83) | 11/5 | 7.7(2.1-16.7)(y) | 0 – 11 (69)  1 – 4 (25)  2 – 1 (6) | I – 6 (38)  II – 7 (44)  III – 3 (19) |
| Hansen 2023 | USA | Retrospective study (real- world data) | NR | 76 (48) | 49 (35)  del(17p), t(14;16), t(4;14) | 6.1m (0.0–13.1) | 159 | idecabtagene/ide-cel/ bb2121 | NA | NA | Second-generation | BCMA | Murine | 64 (36-83) | 91/68 | NR | 0-1 – 127 (81)  2-4 – 29 (19)  Unknown – 31 | I – 22 (17)  II – 71 (55)  III – 35 (27)  Unknown – 31 |
| xu 2024 | China | Prospective study (clinical trial) | 0.07–2.10 × 106 cells/kg | 22 (29.7) | 15 (35.7)  del(17p), t(14;16), t(4;14) | 47.8 m (0.4–60.7) | 74 | LCAR-B38 M | NCT03090659  ChiCTRONH-17012285 | Phase 1 | Second-generation | BCMA | Llama | 54.5 (27∼74) | 45/29 | 4.0(1∼9)(y) | 0-30 (41)  1-32 (43)  2-12 (16) | I-33 (45)  II-14 (19)  III-21 (28)  Unknown-6 (8) |
| Shi 2024 | China | Prospective study (clinical trial) | 1× 106 cells/kg | NR | 34 (68)  amp 1q21, del 17p, del13q,  t(4;14), t(11; 14), and t(14; 16) | 11 m (2.3–24) | 50 | BC19 | ChiCTR 2000033567 | phase I/II | Second-generation | BCMA, CD19 | Humanized | 57 (31–70) | 22/28 | 29.5(4–162)(m) | 0- 22 (44)  1- 23 (46)  2- 5 (10) | I-4 (8)  II-18 (36)  III-28 (56) |
| Shlomit 2024 | Israel | Prospective study (clinical trial) | 150 ×106 cells( 6), 450 ×106 cells(7), 800 ×106 cells(7） | 16 (32) | 34 (68)  del(17p), t(14;16), t(4;14) and 1q gain | 375 days (18-579) | 50 | HBI0101 | NCT 04720313 | Phase 1b/2 | Second-generation | BCMA | Murine | 65 (40-84) | 27/23 | 5.3(0.3-24.2)(y) | 0-13 (26)  1-22 (44)  2-15 (30) | I-13 (29)  II-26 (58)  III-6 (13) |
| Lin 2023 | USA | Prospective study (clinical trial) | 50 × 106 cells(3), 150 × 106 cells(18), 450 × 106 cells(38), 800 × 106 cells(3) | 23 (37) | 17 (27)  del(17p), t(4;14), t(14;16). | 18.1 (1.5–44.5) m | 62 | Idecabtagene vicleucel/ide- cel/bb2121, (CRB-401) | NCT02658929 | Phase 1 | Second-generation | BCMA | Murine | 61 (37–75) | 39/23 | 5.5  (0.8–35.7)(y) | 0-16 (26)  1-44 (71) | NR |
| Bahlis 2023 | USA, Canada | Prospective study (clinical trial) | NR | 17 (31) | 16 (29)  t(4;14), t(14;16) and del(17p) | 12.0 months (range, 0.3‒32.3) | 55 | MagnetisMM-1 | NCT 03269136 | Phase 1 | Second-generation | BCMA,CD3 | humanized | 64.0 (42–80) | 29/26 | NR | 0–1 50(91)  ≥2 – 5 (9) | I-14 (26)  II-20 (36)  III-11 (20)  Unknown-10 (18) |
| Lesokhin 2023 | USA, Canada,  Belgium, France,  Germany,Japan, Spain, UK, Australia | Prospective study (clinical trial) | NR | 39 (32) | 31 (25)  del(17p), t(14;16), t(4;14) | 4.7 months (range: 0.2–25.1 months) | 123 | MagnetisMM-1 | NCT04649359 | Phase 2 | Second-generation | BCMA,CD3 | humanized | 68.0 (36–89) | 68/55 | NR | 0-45 (37）  1-71 (58）  2-7 (5） | I-28 (23)  II-68 (55)  III-19 (15)  Unknown-8 (7) |

| **Supplementary material Table 3. MINORS - Quality Assessment and Kappa coefficient.** | | | | | | | | | | | | | | | | |
| --- | --- | --- | --- | --- | --- | --- | --- | --- | --- | --- | --- | --- | --- | --- | --- | --- |
| **Study** | **MINORS EVALUATION** | | | | | | | | | | | | | | | |
|  | **Clearly stated aim** | | **Inclusion of consecutive patients** | | **Prospective collection of data** | | **Endpoints appropriate to the study** | | **Unbiased assessment of the study endpoint** | | **Follow-up period to to the study** | | **Loss to follow-up less than 5%** | | **Prospective calculation of the study size** | |
|  | **reviewer 1** | **reviewer 2** | **reviewer 1** | **reviewer 2** | **reviewer 1** | **reviewer 2** | **reviewer 1** | **reviewer 2** | **reviewer 1** | **reviewer 2** | **reviewer 1** | **reviewer 2** | **reviewer 1** | **reviewer 2** | **reviewer 1** | **reviewer 2** |
| **Raje 2019** | 2 | 2 | 2 | 2 | 2 | 2 | 2 | 2 | 2 | 2 | 2 | 2 | 2 | 2 | 0 | 0 |
| **Xu 2019** | 2 | 2 | 2 | 2 | 2 | 2 | 1 | 1 | 1 | 1 | 2 | 2 | 2 | 2 | 0 | 0 |
| **Cohen 2019** | 2 | 2 | 2 | 2 | 2 | 2 | 2 | 2 | 2 | 2 | 0 | 0 | 2 | 2 | 0 | 0 |
| **Yan 2020** | 2 | 2 | 2 | 2 | 2 | 2 | 2 | 2 | 1 | 2 | 2 | 2 | 2 | 2 | 0 | 0 |
| **Chen 2020** | 2 | 2 | 2 | 2 | 2 | 2 | 1 | 1 | 1 | 2 | 2 | 1 | 2 | 2 | 0 | 0 |
| **Wang 2021** | 2 | 2 | 2 | 2 | 2 | 2 | 2 | 2 | 1 | 1 | 0 | 0 | 2 | 2 | 0 | 0 |
| **Cornell 2021** | 2 | 2 | 2 | 2 | 2 | 2 | 2 | 2 | 1 | 1 | 2 | 2 | 2 | 2 | 0 | 0 |
| **Mei 2021** | 2 | 2 | 2 | 2 | 2 | 2 | 2 | 2 | 2 | 2 | 2 | 2 | 2 | 2 | 0 | 0 |
| **Munshi 2021** | 2 | 2 | 2 | 2 | 2 | 2 | 2 | 2 | 2 | 2 | 2 | 2 | 2 | 2 | 2 | 1 |
| **Zhang 2022** | 2 | 2 | 2 | 2 | 2 | 2 | 2 | 2 | 2 | 1 | 2 | 2 | 2 | 2 | 0 | 0 |
| **Du 2022** | 2 | 2 | 2 | 2 | 2 | 2 | 2 | 2 | 1 | 1 | 1 | 1 | 2 | 2 | 0 | 0 |
| **Wang 2022** | 2 | 2 | 2 | 2 | 2 | 2 | 1 | 1 | 2 | 2 | 2 | 2 | 2 | 2 | 0 | 0 |
| **Mailankody 2022** | 2 | 2 | 2 | 2 | 2 | 2 | 2 | 2 | 1 | 1 | 2 | 2 | 2 | 2 | 0 | 0 |
| **Qu 2022** | 2 | 2 | 2 | 2 | 2 | 2 | 2 | 2 | 2 | 2 | 2 | 2 | 2 | 2 | 0 | 0 |
| **Tang 2022** | 2 | 2 | 2 | 2 | 2 | 2 | 2 | 2 | 2 | 2 | 1 | 1 | 2 | 2 | 0 | 0 |
| **Ri 2022** | 2 | 2 | 2 | 2 | 2 | 2 | 2 | 2 | 1 | 1 | 2 | 1 | 2 | 2 | 1 | 2 |
| **Martin 2022** | 2 | 2 | 2 | 2 | 2 | 2 | 2 | 1 | 2 | 2 | 1 | 1 | 2 | 2 | 1 | 1 |
| **Mi 2022** | 2 | 2 | 2 | 2 | 2 | 2 | 2 | 2 | 2 | 2 | 2 | 2 | 2 | 2 | 2 | 2 |
| **Asherie 2022** | 2 | 2 | 2 | 2 | 2 | 1 | 2 | 2 | 1 | 1 | 2 | 2 | 2 | 2 | 2 | 2 |
| **Cohen 2023** | 2 | 2 | 2 | 2 | 2 | 2 | 1 | 1 | 2 | 2 | 1 | 1 | 2 | 2 | 0 | 0 |
| **Xia 2023** | 2 | 2 | 2 | 2 | 2 | 2 | 2 | 2 | 1 | 2 | 1 | 1 | 2 | 2 | 0 | 0 |
| **Mailankody 2023** | 2 | 2 | 2 | 2 | 2 | 2 | 1 | 2 | 1 | 1 | 2 | 2 | 2 | 2 | 0 | 0 |
| **Lee 2023** | 2 | 2 | 2 | 2 | 1 | 1 | 2 | 2 | 2 | 2 | 0 | 0 | 2 | 2 | 2 | 1 |
| **Oliver-Caldés 2023** | 2 | 2 | 2 | 2 | 2 | 2 | 2 | 2 | 1 | 1 | 1 | 1 | 2 | 2 | 0 | 0 |
| **Zhang 2023** | 2 | 2 | 2 | 2 | 2 | 2 | 2 | 2 | 1 | 1 | 2 | 2 | 2 | 2 | 0 | 0 |
| **Minakata 2023** | 2 | 2 | 2 | 2 | 2 | 2 | 2 | 2 | 2 | 2 | 2 | 2 | 2 | 2 | 2 | 2 |
| **Sanoyan 2023** | 2 | 2 | 2 | 2 | 2 | 2 | 2 | 2 | 1 | 1 | 1 | 1 | 2 | 2 | 0 | 0 |
| **Hansen 2023** | 2 | 2 | 2 | 2 | 2 | 2 | 2 | 2 | 2 | 2 | 1 | 1 | 2 | 2 | 0 | 0 |
| **xu 2024** | 2 | 2 | 2 | 2 | 2 | 2 | 2 | 2 | 2 | 2 | 2 | 2 | 2 | 2 | 0 | 0 |
| **Shi 2024** | 2 | 2 | 2 | 2 | 2 | 2 | 2 | 2 | 2 | 2 | 2 | 2 | 2 | 2 | 0 | 0 |
| **Shlomit 2024** | 2 | 2 | 2 | 2 | 2 | 2 | 2 | 2 | 2 | 2 | 2 | 2 | 2 | 2 | 1 | 1 |
| **Lin 2023** | 2 | 2 | 2 | 2 | 2 | 2 | 2 | 2 | 2 | 2 | 1 | 1 | 2 | 2 | 0 | 0 |
| **Bahlis 2023** | 2 | 2 | 2 | 2 | 1 | 2 | 2 | 2 | 2 | 1 | 2 | 2 | 2 | 2 | 0 | 0 |
| **Lesokhin 2023** | 2 | 2 | 2 | 2 | 1 | 1 | 2 | 2 | 2 | 2 | 2 | 1 | 2 | 2 | 0 | 0 |
| **kappa coefficient (k)** | **1** | | **1** | | **0.634** | | **0.766** | | **0.693** | | **0.863** | | **1** | | **0.85** | |

| **Supplementary material Table 4. Five Mendelian randomization analyses demonstrate a causal association between immune cells and the development of multiple myeloma** | | | | | | | | | |
| --- | --- | --- | --- | --- | --- | --- | --- | --- | --- |
| **Exposure** | **Panel** | **Method** | **nsnp** | **beta** | **se** | **pval** | **OR** | 95%CI(low) | **95%CI (up)** |
| CD25hi %CD4+ | Treg | MR Egger | 16 | 0.358 | 0.189 | 7.85E-02 | 1.43 | 0.988 | 2.072 |
|  |  | Weighted median |  | 0.301 | 0.138 | 2.90E-02 | 1.351 | 1.031 | 1.771 |
|  |  | Inverse variance weighted |  | 0.327 | 0.105 | 1.85E-03 | 1.387 | 1.129 | 1.704 |
|  |  | Simple mode |  | 0.305 | 0.241 | 2.25E-01 | 1.357 | 0.846 | 2.176 |
|  |  | Weighted mode |  | 0.317 | 0.12 | 1.83E-02 | 1.373 | 1.085 | 1.737 |
| CD28+ CD45RA- CD8dim %CD8dim | Treg | MR Egger | 20 | 0.103 | 0.05 | 5.21E-02 | 1.108 | 1.005 | 1.223 |
|  |  | Weighted median |  | 0.144 | 0.056 | 1.01E-02 | 1.155 | 1.035 | 1.289 |
|  |  | Inverse variance weighted |  | 0.111 | 0.04 | 1.41E-03 | 1.117 | 1.033 | 1.209 |
|  |  | Simple mode |  | 0.102 | 0.092 | 2.81E-01 | 1.107 | 0.925 | 1.326 |
|  |  | Weighted mode |  | 0.123 | 0.053 | 3.31E-02 | 1.131 | 1.019 | 1.255 |
| CD39 on granulocyte | Treg | MR Egger | 17 | -0.273 | 0.105 | 1.97E-02 | 0.761 | 0.62 | 0.935 |
|  |  | Weighted median |  | -0.267 | 0.12 | 2.64E-02 | 0.766 | 0.605 | 0.969 |
|  |  | Inverse variance weighted |  | -0.289 | 0.075 | 5.03E-05 | 0.749 | 0.647 | 0.868 |
|  |  | Simple mode |  | -0.388 | 0.166 | 3.32E-02 | 0.678 | 0.49 | 0.939 |
|  |  | Weighted mode |  | -0.281 | 0.113 | 2.46E-02 | 0.755 | 0.605 | 0.942 |
| DP (CD4+CD8+) %T cell | TBNK | MR Egger | 4 | 1.026 | 0.748 | 3.04E-01 | 2.79 | 0.644 | 12.086 |
|  |  | Weighted median |  | 0.961 | 0.331 | 3.66E-03 | 2.614 | 1.366 | 5.002 |
|  |  | Inverse variance weighted |  | 0.718 | 0.266 | 4.08E-04 | 2.05 | 1.217 | 3.453 |
|  |  | Simple mode |  | 0.903 | 0.423 | 1.22E-01 | 2.467 | 1.077 | 5.652 |
|  |  | Weighted mode |  | 0.918 | 0.366 | 8.72E-02 | 2.504 | 1.222 | 5.131 |
| CD8dim %T cell | TBNK | MR Egger | 15 | -0.407 | 0.299 | 1.97E-01 | 0.666 | 0.37 | 1.196 |
|  |  | Weighted median |  | -0.394 | 0.179 | 2.77E-02 | 0.674 | 0.475 | 0.958 |
|  |  | Inverse variance weighted |  | -0.297 | 0.125 | 1.18E-03 | 0.743 | 0.582 | 0.949 |
|  |  | Simple mode |  | -0.63 | 0.28 | 4.08E-02 | 0.533 | 0.308 | 0.922 |
|  |  | Weighted mode |  | -0.333 | 0.242 | 1.91E-01 | 0.717 | 0.446 | 1.152 |
| CD8dim %leukocyte | TBNK | MR Egger | 13 | -0.122 | 0.259 | 6.46E-01 | 0.885 | 0.533 | 1.471 |
|  |  | Weighted median |  | -0.357 | 0.167 | 3.25E-02 | 0.7 | 0.504 | 0.971 |
|  |  | Inverse variance weighted |  | -0.379 | 0.121 | 1.14E-04 | 0.685 | 0.54 | 0.868 |
|  |  | Simple mode |  | -0.493 | 0.262 | 8.46E-02 | 0.611 | 0.365 | 1.021 |
|  |  | Weighted mode |  | -0.366 | 0.236 | 1.47E-01 | 0.694 | 0.437 | 1.101 |
| HLA DR+ T cell%T cell | TBNK | MR Egger | 25 | -0.188 | 0.065 | 8.31E-03 | 0.829 | 0.729 | 0.941 |
|  |  | Weighted median |  | -0.167 | 0.065 | 1.09E-02 | 0.846 | 0.745 | 0.961 |
|  |  | Inverse variance weighted |  | -0.117 | 0.047 | 9.39E-04 | 0.89 | 0.811 | 0.975 |
|  |  | Simple mode |  | -0.114 | 0.104 | 2.81E-01 | 0.892 | 0.728 | 1.094 |
|  |  | Weighted mode |  | -0.156 | 0.059 | 1.42E-02 | 0.856 | 0.762 | 0.96 |
| CD45 on CD4+ | TBNK | MR Egger | 8 | 0.211 | 0.471 | 6.70E-01 | 1.235 | 0.491 | 3.109 |
|  |  | Weighted median |  | 0.261 | 0.222 | 2.39E-01 | 1.298 | 0.84 | 2.006 |
|  |  | Inverse variance weighted |  | 0.448 | 0.166 | 2.82E-04 | 1.565 | 1.13 | 2.167 |
|  |  | Simple mode |  | 0.193 | 0.352 | 6.01E-01 | 1.213 | 0.608 | 2.418 |
|  |  | Weighted mode |  | 0.197 | 0.37 | 6.11E-01 | 1.218 | 0.59 | 2.515 |
| CD20 on CD20- CD38- | B cell | MR Egger | 8 | 0.144 | 0.16 | 4.03E-01 | 1.155 | 0.844 | 1.58 |
|  |  | Weighted median |  | 0.232 | 0.137 | 8.93E-02 | 1.261 | 0.964 | 1.65 |
|  |  | Inverse variance weighted |  | 0.279 | 0.116 | 7.30E-04 | 1.322 | 1.053 | 1.659 |
|  |  | Simple mode |  | 0.484 | 0.272 | 1.18E-01 | 1.623 | 0.952 | 2.765 |
|  |  | Weighted mode |  | 0.271 | 0.129 | 7.34E-02 | 1.311 | 1.018 | 1.688 |
| CD25 on IgD- CD38br | B cell | MR Egger | 12 | 0.101 | 0.328 | 7.63E-01 | 1.106 | 0.582 | 2.104 |
|  |  | Weighted median |  | 0.181 | 0.185 | 3.28E-03 | 1.198 | 0.834 | 1.722 |
|  |  | Inverse variance weighted |  | 0.454 | 0.19 | 1.88E-03 | 1.575 | 1.085 | 2.285 |
|  |  | Simple mode |  | -0.044 | 0.288 | 8.82E-02 | 0.957 | 0.544 | 1.683 |
|  |  | Weighted mode |  | 0.161 | 0.181 | 3.92E-01 | 1.175 | 0.824 | 1.675 |
| CD4 on naive CD4+ | Maturation stages of T cell | MR Egger | 18 | -0.083 | 0.322 | 8.01E-01 | 0.92 | 0.49 | 1.73 |
|  |  | Weighted median |  | -0.158 | 0.139 | 2.56E-01 | 0.854 | 0.65 | 1.121 |
|  |  | Inverse variance weighted |  | -0.293 | 0.107 | 2.55E-04 | 0.746 | 0.605 | 0.92 |
|  |  | Simple mode |  | -0.06 | 0.219 | 7.86E-01 | 0.942 | 0.613 | 1.447 |
|  |  | Weighted mode |  | -0.089 | 0.204 | 6.67E-01 | 0.915 | 0.613 | 1.365 |
| CD45 on Gr MDSC | Myeloid cell | MR Egger | 8 | -0.213 | 0.154 | 2.14E-01 | 0.808 | 0.598 | 1.093 |
|  |  | Weighted median |  | -0.201 | 0.117 | 8.60E-02 | 0.818 | 0.65 | 1.029 |
|  |  | Inverse variance weighted |  | -0.212 | 0.083 | 7.41E-04 | 0.809 | 0.688 | 0.952 |
|  |  | Simple mode |  | -0.3 | 0.149 | 8.32E-02 | 0.741 | 0.553 | 0.992 |
|  |  | Weighted mode |  | -0.229 | 0.116 | 8.91E-02 | 0.795 | 0.634 | 0.998 |
| HLA DR on CD33br HLA DR+ CD14dim | Myeloid cell | MR Egger | 17 | 0.156 | 0.108 | 1.67E-01 | 1.169 | 0.946 | 1.444 |
|  |  | Weighted median |  | 0.152 | 0.098 | 1.20E-01 | 1.164 | 0.961 | 1.411 |
|  |  | Inverse variance weighted |  | 0.165 | 0.062 | 3.13E-04 | 1.179 | 1.044 | 1.332 |
|  |  | Simple mode |  | 0.128 | 0.145 | 3.90E-01 | 1.137 | 0.855 | 1.51 |
|  |  | Weighted mode |  | 0.139 | 0.089 | 1.36E-01 | 1.149 | 0.965 | 1.368 |
| HLA DR on CD33- HLA DR+ | Myeloid cell | MR Egger | 20 | -0.163 | 0.113 | 1.65E-01 | 0.85 | 0.681 | 1.06 |
|  |  | Weighted median |  | -0.124 | 0.073 | 8.82E-02 | 0.883 | 0.766 | 1.019 |
|  |  | Inverse variance weighted |  | -0.158 | 0.063 | 8.30E-04 | 0.854 | 0.755 | 0.966 |
|  |  | Simple mode |  | -0.322 | 0.161 | 5.98E-02 | 0.725 | 0.529 | 0.994 |
|  |  | Weighted mode |  | -0.151 | 0.081 | 7.79E-02 | 0.86 | 0.734 | 1.008 |
| SSC-A on monocyte | cDC | MR Egger | 32 | -0.094 | 0.085 | 2.81E-01 | 0.91 | 0.771 | 1.075 |
|  |  | Weighted median |  | -0.156 | 0.08 | 5.03E-02 | 0.856 | 0.731 | 1.001 |
|  |  | Inverse variance weighted |  | -0.141 | 0.052 | 5.30E-04 | 0.868 | 0.784 | 0.962 |
|  |  | Simple mode |  | -0.249 | 0.12 | 4.73E-02 | 0.78 | 0.616 | 0.986 |
|  |  | Weighted mode |  | -0.152 | 0.081 | 6.86E-02 | 0.859 | 0.733 | 1.007 |
| Significant outcomes from five Mendelian analyses of immune cells with a corrected P-value <0.10 based on IVW results. OR, odd ratio; CI, condifence interval | | | | | | | | | |

| **Supplementary material Table 5. Sensitivity analysis results of immune cell populations associated with multiple myeloma.** | | | | | | | | | | | | |
| --- | --- | --- | --- | --- | --- | --- | --- | --- | --- | --- | --- | --- |
| **Exposure** | **nsnp** | **MR Egger** | | **Inverse variance weighted** | | **I^2** | **F statistic** | **MR.PRESSO** | | **Pleiotropy** | | |
|  |  | **Q** | **Pvalue** | **Q** | **Pvalue** |  |  | **Global.Test.** | **Pvalue** | **egger intercept** | **se** | **pval** |
| CD25hi %CD4+ | 16 | 25.71068221 | 0.028166278 | 27.5378657 | 0.02464753 | 23.75% | 12.88673 | 36.37466183 | 0.109 | -0.05780377 | 0.05795059 | 0.335467018 |
| CD28+ CD45RA- CD8dim %CD8dim | 20 | 20.48484124 | 0.30619487 | 20.6806809 | 0.354710051 | 0.00% | 26.7756739 | 26.87647103 | 0.798 | -0.013460585 | 0.032448428 | 0.683168573 |
| CD39 on granulocyte | 17 | 7.830207793 | 0.930409211 | 7.880637871 | 0.952340564 | 0.00% | 10.2775384 | 14.28365413 | 0.895 | -0.006473398 | 0.028826208 | 0.825346943 |
| DP (CD4+CD8+) %T cell | 4 | 2.968756742 | 0.226643185 | 3.273651161 | 0.351326781 | 0.00% | 8.77376193 | 11.66381335 | 0.675 | -0.059867755 | 0.132096267 | 0.694818265 |
| CD8dim %T cell | 15 | 14.3190886 | 0.351762186 | 14.50056612 | 0.41312256 | 0.00% | 19.8552834 | 9.48476293 | 0.584 | 0.032556663 | 0.080207407 | 0.691413622 |
| CD8dim %leukocyte | 13 | 8.469832444 | 0.670700667 | 9.722982722 | 0.64024865 | 0.00% | 10.8365823 | 17.7366235 | 0.691 | -0.076735314 | 0.068547828 | 0.286799971 |
| HLA DR+ T cell%T cell | 25 | 14.48000077 | 0.912297595 | 17.04009459 | 0.846946976 | 0.00% | 29.8772193 | 30.86618923 | 0.385 | 0.04078768 | 0.025491833 | 0.123239811 |
| CD45 on CD4+ | 8 | 4.982962204 | 0.5460002 | 5.273898224 | 0.626576916 | 0.00% | 9.2889971 | 7.383866913 | 0.748 | 0.040048257 | 0.07424802 | 0.609039006 |
| CD20 on CD20- CD38- | 8 | 6.718758446 | 0.347641052 | 8.286399873 | 0.308018237 | 0.00% | 7.8762333 | 8.886646236 | 0.568 | 0.062211963 | 0.05257986 | 0.281485512 |
| CD25 on IgD- CD38br | 12 | 24.08574145 | 0.007376031 | 25.35693965 | 0.008080367 | 70.78% | 18.7792863 | 29.7657236 | 0.093 | 0.048731764 | 0.067078835 | 0.484199409 |
| CD4 on naive CD4+ | 18 | 21.03151874 | 0.177302205 | 21.66684634 | 0.197873079 | 8.38% | 25.7992373 | 19.93763833 | 0.376 | -0.043778941 | 0.062971164 | 0.496889813 |
| CD45 on Gr MDSC | 8 | 3.980831669 | 0.679270548 | 3.980986454 | 0.781964782 | 0.00% | 10.7286643 | 15.99267364 | 0.267 | 0.000654576 | 0.052613406 | 0.990476951 |
| HLA DR on CD33br HLA DR+ CD14dim | 17 | 14.98538113 | 0.452470753 | 14.99644172 | 0.524899157 | 2.35% | 26.8899243 | 26.88355727 | 0.197 | 0.00416645 | 0.039616589 | 0.917634913 |
| HLA DR on CD33- HLA DR+ | 20 | 28.55927904 | 0.054038537 | 28.78959464 | 0.069367536 | 11.84% | 15.29768823 | 38.75599376 | 0.376 | 0.017382639 | 0.045623793 | 0.707663196 |
| SSC-A on monocyte | 32 | 24.93094117 | 0.728381021 | 25.4113516 | 0.749021955 | 0.00% | 38.8861793 | 40.28666512 | 0.618 | -0.021760156 | 0.03139466 | 0.493567946 |
| Cochran Q statistic implemented in MR Egger and IVW method, P>0.05 indicates no heterogeneity exists. The intercept of MR Egger can be used to indicate whether directional horizontal pleiotropy is driving the results of MR analysis, there are no directional pleiotropies if P>0.05.  MR-PRESSO can detect and adjust for any outliers reflecting horizontal pleiotropic biases, where p value for Global test > 0.05 indicates no horizontal pleiotropic outliers. The I2 statistic was calculated to assess the heterogeneity of each outcome from different data sources, and the I2values <25%，25-75%,and >75% were considered to indicate low moderate,and high heterogeneity, respectively. | | | | | | | | | | | | |

| **Supplementary material Table 6. SMR analysis results from eQTL of immune cells causally associated with multiple myeloma.** | | | | | | | | | | | | | | | | | | | | | | | | | | | | | |  |
| --- | --- | --- | --- | --- | --- | --- | --- | --- | --- | --- | --- | --- | --- | --- | --- | --- | --- | --- | --- | --- | --- | --- | --- | --- | --- | --- | --- | --- | --- | --- |
| **Type of immune cell** | **Probe** | **Gene Chr.** | **Gene** | **Probe base pair** | **topSNP** | **Gene Chr.** | **Probe base pair** | **Effect allele** | **Other allele** | **Effect allele frequence** | **GWAS association** | | | **eQTL association** | | | | **SMR association** | | | | | | **HEIDI Test** | | | | **BH correction** | |  |
|  |  |  |  |  |  |  |  |  |  |  | **β** | **SE** | **P** | **β** | **SE** | **P** | | **β** | | **SE** | | **P** | | **P** | | **nsnp** | | **P-FDR** | |  |
| CD25hi %CD4+ | ENSG00000183625 | 3 | CCR3 | 46205096 | rs2201150 | 3 | 46258902 | C | T | 0.642147 | -0.0578816 | 0.0402549 | 0.150469 | 0.42482 | 0.033333 | 3.33E-37 | | -0.13625 | | 0.0953587 | | 0.1530581 | | 0.4080526 | | 20 | | 0.619769443 | |  |
| DP (CD4+CD8+) %T cell | ENSG00000143799 | 1 | PARP1 | 226548392 | rs6661762 | 1 | 226543691 | A | C | 0.16998 | 0.121518 | 0.0535562 | 0.0232691 | 0.0945854 | 0.0170385 | 2.84E-08 | | 1.28474 | | 0.611692 | | 0.03570133 | | NA | | NA | | 0.68365314 | |  |
|  | ENSG00000116062 | 2 | MSH6 | 47922669 | rs6742037 | 2 | 48103505 | C | A | 0.373757 | 0.0996883 | 0.0412754 | 0.0157264 | -0.269311 | 0.0270485 | 2.36E-23 | | -0.370161 | | 0.157708 | | 0.01891858 | | 0.735255 | | 20 | | 0.583773326 | |  |
|  | ENSG00000144554 | 3 | FANCD2 | 10068098 | rs7641192 | 3 | 10121104 | T | G | 0.215706 | -0.146989 | 0.052422 | 0.00504801 | -0.195945 | 0.0268897 | 3.17E-13 | | 0.750154 | | 0.286657 | | 0.000887303 | | 0.7051949 | | 18 | | 0.048876203 | |  |
|  | ENSG00000134086 | 3 | VHL | 10182692 | rs412210 | 3 | 10199444 | G | C | 0.699801 | 0.136371 | 0.0460093 | 0.00303683 | 0.115304 | 0.0208619 | 3.26E-08 | | 1.18271 | | 0.452783 | | 0.000899912 | | 0.5314812 | | 12 | | 0.048876203 | |  |
|  | ENSG00000135605 | 4 | TEC | 48137800 | rs10028155 | 4 | 47906812 | A | C | 0.328032 | 0.0854495 | 0.0395586 | 0.0307666 | 0.229436 | 0.0307138 | 8.01E-14 | | 0.372433 | | 0.17948 | | 0.03798073 | | 0.3067394 | | 20 | | 0.68365314 | |  |
|  | ENSG00000170606 | 5 | HSPA4 | 132387654 | rs72801474 | 5 | 132444128 | A | G | 0.0685885 | -0.252321 | 0.0886916 | 0.00444222 | -0.355222 | 0.0411964 | 6.54E-18 | | 0.710319 | | 0.262918 | | 0.006899135 | | 0.5593649 | | 7 | | 0.388762027 | |  |
|  | ENSG00000113263 | 5 | ITK | 156569944 | rs13355489 | 5 | 156664822 | C | T | 0.138171 | 0.133571 | 0.057389 | 0.0199407 | 0.0728742 | 0.0133178 | 4.45E-08 | | 1.8329 | | 0.855785 | | 0.03221172 | | 0.8788798 | | 4 | | 0.68365314 | |  |
|  | ENSG00000204632 | 6 | HLA-G | 29794744 | rs3130643 | 6 | 30766634 | T | A | 0.854871 | -0.18337 | 0.0604248 | 0.00240791 | 0.489873 | 0.066645 | 1.97E-13 | | -0.374322 | | 0.133447 | | 0.005031257 | | 0.1374094 | | 20 | | 0.388762027 | |  |
|  | ENSG00000196126 | 6 | HLA-DRB1 | 32546546 | rs113568276 | 6 | 32513127 | A | G | 0.257455 | -0.120501 | 0.0475651 | 0.0112964 | -0.235707 | 0.0189896 | 2.24E-35 | | 0.511232 | | 0.205958 | | 0.01305676 | | 0.9991782 | | 20 | | 0.47004336 | |  |
|  | ENSG00000237541 | 6 | HLA-DQA2 | 32709119 | rs9272358 | 6 | 32604538 | A | G | 0.370775 | -0.0823102 | 0.0417888 | 0.0488754 | 1.04878 | 0.0420171 | 1.63E-137 | | -0.0784819 | | 0.039969 | | 0.04958029 | | 0.2012622 | | 20 | | 0.74895561 | |  |
|  | ENSG00000107796 | 10 | ACTA2 | 90694831 | rs6586165 | 10 | 90755056 | A | T | 0.468191 | -0.0823118 | 0.0391709 | 0.0356098 | -0.484126 | 0.0308371 | 1.52E-55 | | 0.170021 | | 0.0816321 | | 0.03727158 | | 0.8585164 | | 20 | | 0.68365314 | |  |
|  | ENSG00000111424 | 12 | VDR | 48235320 | rs12721375 | 12 | 48282900 | A | G | 0.144135 | -0.226032 | 0.054052 | 2.89E-05 | -0.312205 | 0.0384655 | 4.80E-16 | | 0.723986 | | 0.194757 | | 0.000201306 | | 0.1024755 | | 6 | | 0.043482053 | |  |
|  | ENSG00000129315 | 12 | CCNT1 | 49082247 | rs10875859 | 12 | 49074762 | A | C | 0.26839 | 0.0917974 | 0.042097 | 0.0292119 | -0.134609 | 0.0189558 | 1.24E-12 | | -0.681956 | | 0.327148 | | 0.03711042 | | 0.03285984 | | 12 | | 0.68365314 | |  |
| CD8dim %T cell | ENSG00000243772 | 19 | KIR2DL3 | 55249980 | rs202170060 | 19 | 55237677 | T | C | 0.0437376 | -0.0473195 | 0.0636772 | 0.457412 | 0.468916 | 0.0648308 | 4.73E-13 | | -0.100913 | | 0.136511 | | 0.459771 | | 0.1686071 | | 8 | | 0.9757794 | |  |
| CD8dim %leukocyte | ENSG00000206344 | 6 | HCG27 | 31165537 | rs9366770 | 6 | 31170228 | C | G | 0.481113 | -0.12488 | 0.0389236 | 0.00133503 | 0.206167 | 0.0178765 | 9.01E-31 | | -0.605723 | | 0.195966 | | 0.001995108 | | 0.6736373 | | 20 | | 0.113721156 | |  |
|  | ENSG00000204525 | 6 | HLA-C | 31236526 | rs2524105 | 6 | 31232910 | C | G | 0.409543 | -0.0706177 | 0.0393293 | 0.0725655 | -0.448997 | 0.0255675 | 4.88E-69 | | 0.157279 | | 0.0880503 | | 0.07406091 | | 0.3467801 | | 20 | | 0.71592931 | |  |
|  | ENSG00000171631 | 11 | P2RY6 | 72975550 | rs7942649 | 11 | 73000403 | C | T | 0.166998 | 0.13457 | 0.0544055 | 0.013381 | 0.275617 | 0.0478339 | 8.31E-09 | | 0.48825 | | 0.214814 | | 0.02303267 | | 0.04259384 | | 3 | | 0.656431095 | |  |
|  | ENSG00000089041 | 12 | P2RX7 | 121570622 | rs71454691 | 12 | 121786739 | T | C | 0.0119284 | -0.832354 | 0.415227 | 0.045008 | -0.670054 | 0.103142 | 8.23E-11 | | 1.24222 | | 0.648523 | | 0.05543388 | | NA | | NA | | 0.71592931 | |  |
| HLA DR+ T cell%T cell | ENSG00000143799 | 1 | PARP1 | 226548392 | rs6661762 | 1 | 226543691 | A | C | 0.16998 | 0.121518 | 0.0535562 | 0.0232691 | 0.0945854 | 0.0170385 | 2.84E-08 | | 1.28474 | | 0.611692 | | 0.03570133 | | NA | | NA | | 0.803812609 | |  |
|  | ENSG00000168918 | 2 | INPP5D | 233924677 | rs6715767 | 2 | 234074817 | A | G | 0.432406 | -0.0786346 | 0.0390899 | 0.0442588 | -0.171861 | 0.0212248 | 5.63E-16 | | 0.457548 | | 0.234365 | | 0.050904 | | 0.4857735 | | 20 | | 0.803812609 | |  |
|  | ENSG00000113263 | 5 | ITK | 156569944 | rs13355489 | 5 | 156664822 | C | T | 0.138171 | 0.133571 | 0.057389 | 0.0199407 | 0.0728742 | 0.0133178 | 4.45E-08 | | 1.8329 | | 0.855785 | | 0.03221172 | | 0.8788798 | | 4 | | 0.803812609 | |  |
|  | ENSG00000204632 | 6 | HLA-G | 29794744 | rs3130643 | 6 | 30766634 | T | A | 0.854871 | -0.18337 | 0.0604248 | 0.00240791 | 0.489873 | 0.066645 | 1.97E-13 | | -0.374322 | | 0.133447 | | 0.005031257 | | 0.1374094 | | 20 | | 0.439396445 | |  |
|  | ENSG00000206344 | 6 | HCG27 | 31165537 | rs9366770 | 6 | 31170228 | C | G | 0.481113 | -0.12488 | 0.0389236 | 0.00133503 | 0.206167 | 0.0178765 | 9.01E-31 | | -0.605723 | | 0.195966 | | 0.001995108 | | 0.6736373 | | 20 | | 0.261359148 | | |
|  | ENSG00000237541 | 6 | HLA-DQA2 | 32709119 | rs9272358 | 6 | 32604538 | A | G | 0.370775 | -0.0823102 | 0.0417888 | 0.0488754 | 1.04878 | 0.0420171 | | 1.63E-137 | | -0.0784819 | | 0.039969 | | 0.04958029 | | 0.2012622 | | 20 | | 0.803812609 | |
|  | ENSG00000106976 | 9 | DNM1 | 130965658 | rs10760548 | 9 | 130965235 | A | G | 0.988072 | -0.296469 | 0.143975 | 0.0394775 | -0.875681 | 0.0944891 | | 1.91E-20 | | 0.338558 | | 0.168425 | | 0.04441552 | | 0.6131758 | | 3 | | 0.803812609 | |
|  | ENSG00000065618 | 10 | COL17A1 | 105791044 | rs2181833 | 10 | 105793369 | A | G | 0.807157 | -0.112166 | 0.0461637 | 0.0151095 | -0.33031 | 0.0305137 | | 2.62E-27 | | 0.339578 | | 0.143236 | | 0.01775167 | | 0.3332134 | | 20 | | 0.803812609 | |
|  | ENSG00000167323 | 11 | STIM1 | 3875757 | rs10160415 | 11 | 3909766 | C | G | 0.328032 | 0.0912236 | 0.0402456 | 0.0234099 | 0.117371 | 0.020542 | | 1.11E-08 | | 0.777224 | | 0.368888 | | 0.03512312 | | 0.9870692 | | 4 | | 0.803812609 | |
|  | ENSG00000255717 | 11 | SNHG1 | 62619460 | rs11231252 | 11 | 62624771 | A | G | 0.0437376 | -0.165753 | 0.077421 | 0.0322797 | 0.15327 | 0.0233606 | | 5.34E-11 | | -1.08144 | | 0.53134 | | 0.04181932 | | NA | | NA | | 0.803812609 | |
|  | ENSG00000111424 | 12 | VDR | 48235320 | rs12721375 | 12 | 48282900 | A | G | 0.144135 | -0.226032 | 0.054052 | 2.89E-05 | -0.312205 | 0.0384655 | | 4.80E-16 | | 0.723986 | | 0.194757 | | 0.000201306 | | 0.1024755 | | 6 | | 0.045274212 | |
|  | ENSG00000129315 | 12 | CCNT1 | 49082247 | rs10875859 | 12 | 49074762 | A | C | 0.26839 | 0.0917974 | 0.042097 | 0.0292119 | -0.134609 | 0.0189558 | | 1.24E-12 | | -0.681956 | | 0.327148 | | 0.03711042 | | 0.03285984 | | 12 | | 0.803812609 | |
|  | ENSG00000213918 | 16 | DNASE1 | 3661729 | rs9989424 | 16 | 3672587 | C | G | 0.312127 | -0.102001 | 0.0421503 | 0.0155235 | -0.095459 | 0.0167327 | | 1.16E-08 | | 1.06853 | | 0.479636 | | 0.02589414 | | 0.1072935 | | 15 | | 0.803812609 | |
|  | ENSG00000215424 | 21 | MCM3AP-AS1 | 47649131 | rs16979009 | 21 | 47665790 | G | A | 0.0109344 | 0.674833 | 0.317425 | 0.0335066 | 0.834145 | 0.0835384 | | 1.77E-23 | | 0.809012 | | 0.389069 | | 0.03758494 | | 0.05947956 | | 5 | | 0.803812609 | |
|  | ENSG00000239713 | 22 | APOBEC3G | 39436924 | rs28583464 | 22 | 39486593 | C | T | 0.0815109 | 0.160571 | 0.0664663 | 0.0156993 | -0.396673 | 0.0393885 | | 7.44E-24 | | -0.404794 | | 0.172313 | | 0.01881482 | | 0.5116796 | | 15 | | 0.803812609 | |
| CD20 on CD20- CD38- | ENSG00000177000 | 1 | MTHFR | 11845780 | rs147124396 | 1 | 11880749 | A | G | 0.0974155 | 0.123104 | 0.0621221 | 0.0475193 | 0.737566 | 0.0508756 | | 1.26E-47 | | 0.166906 | | 0.085009 | | 0.04960102 | | 0.4216706 | | 20 | | 0.645791394 | |
|  | ENSG00000143799 | 1 | PARP1 | 226548392 | rs6661762 | 1 | 226543691 | A | C | 0.16998 | 0.121518 | 0.0535562 | 0.0232691 | 0.0945854 | 0.0170385 | | 2.84E-08 | | 1.28474 | | 0.611692 | | 0.03570133 | | NA | | NA | | 0.628343408 | |
|  | ENSG00000196126 | 6 | HLA-DRB1 | 32546546 | rs113568276 | 6 | 32513127 | A | G | 0.257455 | -0.120501 | 0.0475651 | 0.0112964 | -0.235707 | 0.0189896 | | 2.24E-35 | | 0.511232 | | 0.205958 | | 0.01305676 | | 0.9991782 | | 20 | | 0.57449744 | |
|  | ENSG00000167323 | 11 | STIM1 | 3875757 | rs10160415 | 11 | 3909766 | C | G | 0.328032 | 0.0912236 | 0.0402456 | 0.0234099 | 0.117371 | 0.020542 | | 1.11E-08 | | 0.777224 | | 0.368888 | | 0.03512312 | | 0.9870692 | | 4 | | 0.628343408 | |
|  | ENSG00000111424 | 12 | VDR | 48235320 | rs12721375 | 12 | 48282900 | A | G | 0.144135 | -0.226032 | 0.054052 | 2.89E-05 | -0.312205 | 0.0384655 | | 4.80E-16 | | 0.723986 | | 0.194757 | | 0.000201306 | | 0.1024755 | | 6 | | 0.01771491 | |
|  | ENSG00000159110 | 21 | IFNAR2 | 34602206 | rs1131964 | 21 | 34610487 | C | T | 0.543738 | 0.099566 | 0.038872 | 0.0104258 | -0.0940295 | 0.0171698 | | 4.34E-08 | | -1.05888 | | 0.456384 | | 0.02033254 | | 0.3155707 | | 3 | | 0.596421173 | |
|  | ENSG00000100197 | 22 | CYP2D6 | 42522501 | rs3021083 | 22 | 42538103 | T | C | 0.535785 | -0.0765283 | 0.0391131 | 0.0503953 | 0.769005 | 0.0361238 | | 1.46E-100 | | -0.099516 | | 0.0510763 | | 0.05136977 | | 0.6652092 | | 20 | | 0.645791394 | |
| CD25 on IgD- CD38br | ENSG00000111424 | 12 | VDR | 48235320 | rs12721375 | 12 | 48282900 | A | G | 0.144135 | -0.226032 | 0.054052 | 2.89E-05 | -0.312205 | 0.0384655 | | 4.80E-16 | | 0.723986 | | 0.194757 | | 0.000201306 | | 0.1024755 | | 6 | | 0.005837868 | |
|  | ENSG00000115138 | 2 | POMC | 25383722 | rs3754861 | 2 | 25393722 | C | A | 0.0785288 | 0.140606 | 0.0721037 | 0.0511694 | 0.606297 | 0.0816465 | | 1.12E-13 | | 0.231909 | | 0.122957 | | 0.002928097 | | 0.07315123 | | 9 | | 0.042457407 | |
| CD45 on CD4+ | ENSG00000117586 | 1 | TNFSF4 | 173152873 | rs4916314 | 1 | 173172158 | A | C | 0.194831 | -0.102694 | 0.0499847 | 0.0399255 | -0.18668 | 0.0313977 | | 2.75E-09 | | 0.550107 | | 0.283291 | | 0.05215578 | | 0.3717081 | | 5 | | 0.945157369 | |
|  | ENSG00000113263 | 5 | ITK | 156569944 | rs13355489 | 5 | 156664822 | C | T | 0.138171 | 0.133571 | 0.057389 | 0.0199407 | 0.0728742 | 0.0133178 | | 4.45E-08 | | 1.8329 | | 0.855785 | | 0.03221172 | | 0.8788798 | | 4 | | 0.945157369 | |
|  | ENSG00000204632 | 6 | HLA-G | 29794744 | rs3130643 | 6 | 30766634 | T | A | 0.854871 | -0.18337 | 0.0604248 | 0.00240791 | 0.489873 | 0.066645 | | 1.97E-13 | | -0.374322 | | 0.133447 | | 0.005031257 | | 0.1374094 | | 20 | | 0.613813354 | |
|  | ENSG00000196126 | 6 | HLA-DRB1 | 32546546 | rs113568276 | 6 | 32513127 | A | G | 0.257455 | -0.120501 | 0.0475651 | 0.0112964 | -0.235707 | 0.0189896 | | 2.24E-35 | | 0.511232 | | 0.205958 | | 0.01305676 | | 0.9991782 | | 20 | | 0.79646236 | |
|  | ENSG00000237541 | 6 | HLA-DQA2 | 32709119 | rs9272358 | 6 | 32604538 | A | G | 0.370775 | -0.0823102 | 0.0417888 | 0.0488754 | 1.04878 | 0.0420171 | | 1.63E-137 | | -0.0784819 | | 0.039969 | | 0.04958029 | | 0.2012622 | | 20 | | 0.945157369 | |
| CD4 on naive CD4+ | ENSG00000111424 | 12 | VDR | 48235320 | rs12721375 | 12 | 48282900 | A | G | 0.144135 | -0.226032 | 0.054052 | 2.89E-05 | -0.312205 | 0.0384655 | | 4.80E-16 | | 0.723986 | | 0.194757 | | 0.000201306 | | 0.1024755 | | 6 | | 0.04682809 | |
|  | ENSG00000204632 | 6 | HLA-G | 29794744 | rs3130643 | 6 | 30766634 | T | A | 0.854871 | -0.18337 | 0.0604248 | 0.00240791 | 0.489873 | 0.066645 | | 1.97E-13 | | -0.374322 | | 0.133447 | | 0.005031257 | | 0.1374094 | | 20 | | 0.606265625 | |
|  | ENSG00000170606 | 5 | HSPA4 | 132387654 | rs72801474 | 5 | 132444128 | A | G | 0.0685885 | -0.252321 | 0.0886916 | 0.00444222 | -0.355222 | 0.0411964 | | 6.54E-18 | | 0.710319 | | 0.262918 | | 0.006899135 | | 0.5593649 | | 7 | | 0.606265625 | |
|  | ENSG00000135828 | 1 | RNASEL | 182542769 | rs12041623 | 1 | 182561633 | C | T | 0.370775 | -0.116285 | 0.0403877 | 0.00398648 | 0.0953317 | 0.0146109 | | 6.81E-11 | | -1.21979 | | 0.46307 | | 0.008434899 | | 0.1226285 | | 3 | | 0.606265625 | |
|  | ENSG00000196843 | 2 | ARID5A | 97202480 | rs4907242 | 2 | 97273113 | A | C | 0.271372 | -0.137252 | 0.0503702 | 0.00643265 | -0.0953056 | 0.0156065 | | 1.02E-09 | | 1.44013 | | 0.578739 | | 0.01283244 | | 0.05232127 | | 11 | | 0.606265625 | |
|  | ENSG00000196126 | 6 | HLA-DRB1 | 32546546 | rs113568276 | 6 | 32513127 | A | G | 0.257455 | -0.120501 | 0.0475651 | 0.0112964 | -0.235707 | 0.0189896 | | 2.24E-35 | | 0.511232 | | 0.205958 | | 0.01305676 | | 0.9991782 | | 20 | | 0.75983499 | |
|  | ENSG00000187742 | 9 | SECISBP2 | 91933421 | rs78472267 | 9 | 91982902 | C | T | 0.0914513 | 0.144755 | 0.0584359 | 0.0132437 | 0.396568 | 0.0512029 | | 9.56E-15 | | 0.365019 | | 0.154707 | | 0.01830371 | | 0.2550732 | | 18 | | 0.75983499 | |
|  | ENSG00000239713 | 22 | APOBEC3G | 39436924 | rs28583464 | 22 | 39486593 | C | T | 0.0815109 | 0.160571 | 0.0664663 | 0.0156993 | -0.396673 | 0.0393885 | | 7.44E-24 | | -0.404794 | | 0.172313 | | 0.01881482 | | 0.5116796 | | 15 | | 0.75983499 | |
|  | ENSG00000116062 | 2 | MSH6 | 47922669 | rs6742037 | 2 | 48103505 | C | A | 0.373757 | 0.0996883 | 0.0412754 | 0.0157264 | -0.269311 | 0.0270485 | | 2.36E-23 | | -0.370161 | | 0.157708 | | 0.01891858 | | 0.735255 | | 20 | | 0.75983499 | |
|  | ENSG00000083444 | 1 | PLOD1 | 11994262 | rs873458 | 1 | 12046089 | A | G | 0.526839 | 0.0955652 | 0.0405749 | 0.0185089 | -0.377096 | 0.0227202 | | 7.28E-62 | | -0.253424 | | 0.108676 | | 0.0197051 | | 0.01059962 | | 20 | | 0.75983499 | |
|  | ENSG00000159110 | 21 | IFNAR2 | 34602206 | rs1131964 | 21 | 34610487 | C | T | 0.543738 | 0.099566 | 0.038872 | 0.0104258 | -0.0940295 | 0.0171698 | | 4.34E-08 | | -1.05888 | | 0.456384 | | 0.02033254 | | 0.3155707 | | 3 | | 0.75983499 | |
|  | ENSG00000204713 | 6 | TRIM27 | 28870779 | rs1061625 | 6 | 28891458 | C | T | 0.322068 | 0.105051 | 0.0454822 | 0.0209035 | 0.312641 | 0.0199027 | | 1.32E-55 | | 0.336012 | | 0.147042 | | 0.02230423 | | 0.08344913 | | 20 | | 0.75983499 | |
|  | ENSG00000113263 | 5 | ITK | 156569944 | rs13355489 | 5 | 156664822 | C | T | 0.138171 | 0.133571 | 0.057389 | 0.0199407 | 0.0728742 | 0.0133178 | | 4.45E-08 | | 1.8329 | | 0.855785 | | 0.03221172 | | 0.8788798 | | 4 | | 0.75983499 | |
|  | ENSG00000105193 | 19 | RPS16 | 39923847 | rs640000 | 19 | 39926601 | T | C | 0.39165 | 0.0882841 | 0.0399824 | 0.0272396 | 0.0937805 | 0.0112763 | | 9.05E-17 | | 0.941391 | | 0.441111 | | 0.03283163 | | 0.6065952 | | 15 | | 0.75983499 | |
|  | ENSG00000167323 | 11 | STIM1 | 3875757 | rs10160415 | 11 | 3909766 | C | G | 0.328032 | 0.0912236 | 0.0402456 | 0.0234099 | 0.117371 | 0.020542 | | 1.11E-08 | | 0.777224 | | 0.368888 | | 0.03512312 | | 0.9870692 | | 4 | | 0.75983499 | |
|  | ENSG00000143799 | 1 | PARP1 | 226548392 | rs6661762 | 1 | 226543691 | A | C | 0.16998 | 0.121518 | 0.0535562 | 0.0232691 | 0.0945854 | 0.0170385 | | 2.84E-08 | | 1.28474 | | 0.611692 | | 0.03570133 | | NA | | NA | | 0.75983499 | |
|  | ENSG00000149177 | 11 | PTPRJ | 48002113 | rs747782 | 11 | 47940925 | C | T | 0.192843 | 0.104175 | 0.0476583 | 0.0288257 | 0.122888 | 0.0167627 | | 2.28E-13 | | 0.847723 | | 0.404691 | | 0.03619404 | | 0.1457813 | | 20 | | 0.75983499 | |
|  | ENSG00000129315 | 12 | CCNT1 | 49082247 | rs10875859 | 12 | 49074762 | A | C | 0.26839 | 0.0917974 | 0.042097 | 0.0292119 | -0.134609 | 0.0189558 | | 1.24E-12 | | -0.681956 | | 0.327148 | | 0.03711042 | | 0.03285984 | | 12 | | 0.75983499 | |
|  | ENSG00000107796 | 10 | ACTA2 | 90694831 | rs6586165 | 10 | 90755056 | A | T | 0.468191 | -0.0823118 | 0.0391709 | 0.0356098 | -0.484126 | 0.0308371 | | 1.52E-55 | | 0.170021 | | 0.0816321 | | 0.03727158 | | 0.8585164 | | 20 | | 0.75983499 | |
|  | ENSG00000154229 | 17 | PRKCA | 64298754 | rs72843895 | 17 | 64331156 | G | T | 0.424453 | 0.0887946 | 0.0408209 | 0.0296135 | 0.166917 | 0.0253913 | | 4.91E-11 | | 0.531969 | | 0.257599 | | 0.03891308 | | 0.8232161 | | 13 | | 0.75983499 | |
|  | ENSG00000255717 | 11 | SNHG1 | 62619460 | rs11231252 | 11 | 62624771 | A | G | 0.0437376 | -0.165753 | 0.077421 | 0.0322797 | 0.15327 | 0.0233606 | | 5.34E-11 | | -1.08144 | | 0.53134 | | 0.04181932 | | NA | | NA | | 0.75983499 | |
|  | ENSG00000054598 | 6 | FOXC1 | 1610681 | rs12206258 | 6 | 1709703 | C | T | 0.327038 | 0.0849803 | 0.0416129 | 0.0411358 | -0.312052 | 0.0301992 | | 4.99E-25 | | -0.272327 | | 0.135932 | | 0.0451331 | | 0.3827458 | | 20 | | 0.75983499 | |
|  | ENSG00000160789 | 1 | LMNA | 156052364 | rs582690 | 1 | 156088256 | G | C | 0.0646123 | -0.247226 | 0.119842 | 0.0391201 | 0.400791 | 0.0498887 | | 9.46E-16 | | -0.616845 | | 0.308715 | | 0.04570491 | | 0.7326794 | | 6 | | 0.75983499 | |
|  | ENSG00000126746 | 12 | ZNF384 | 6775643 | rs7964510 | 12 | 6787622 | T | C | 0.0964215 | 0.177644 | 0.0886138 | 0.0449956 | 0.353992 | 0.0359723 | | 7.52E-23 | | 0.501831 | | 0.255469 | | 0.04948916 | | 0.2457447 | | 16 | | 0.75983499 | |
|  | ENSG00000237541 | 6 | HLA-DQA2 | 32709119 | rs9272358 | 6 | 32604538 | A | G | 0.370775 | -0.0823102 | 0.0417888 | 0.0488754 | 1.04878 | 0.0420171 | | 1.63E-137 | | -0.0784819 | | 0.039969 | | 0.04958029 | | 0.2012622 | | 20 | | 0.75983499 | |
|  | ENSG00000177000 | 1 | MTHFR | 11845780 | rs147124396 | 1 | 11880749 | A | G | 0.0974155 | 0.123104 | 0.0621221 | 0.0475193 | 0.737566 | 0.0508756 | | 1.26E-47 | | 0.166906 | | 0.085009 | | 0.04960102 | | 0.4216706 | | 20 | | 0.75983499 | |
| CD39 on granulocyte | ENSG00000206344 | 6 | HCG27 | 31165537 | rs9366770 | 6 | 31170228 | C | G | 0.481113 | -0.12488 | 0.0389236 | 0.00133503 | 0.206167 | 0.0178765 | | 9.01E-31 | | -0.605723 | | 0.195966 | | 0.001995108 | | 0.6736373 | | 20 | | 0.113721156 | |
|  | ENSG00000171631 | 11 | P2RY6 | 72975550 | rs7942649 | 11 | 73000403 | C | T | 0.166998 | 0.13457 | 0.0544055 | 0.013381 | 0.275617 | 0.0478339 | | 8.31E-09 | | 0.48825 | | 0.214814 | | 0.02303267 | | 0.04259384 | | 3 | | 0.656431095 | |
|  | ENSG00000089041 | 12 | P2RX7 | 121570622 | rs71454691 | 12 | 121786739 | T | C | 0.0119284 | -0.832354 | 0.415227 | 0.045008 | -0.670054 | 0.103142 | | 8.23E-11 | | 1.24222 | | 0.648523 | | 0.05543388 | | NA | | NA | | 0.71592931 | |
| CD45 on Gr MDSC | ENSG00000204632 | 6 | HLA-G | 29794744 | rs3130643 | 6 | 30766634 | T | A | 0.854871 | -0.18337 | 0.0604248 | 0.00240791 | 0.489873 | 0.066645 | | 1.97E-13 | | -0.374322 | | 0.133447 | | 0.005031257 | | 0.1374094 | | 20 | | 0.10062514 | |
|  | ENSG00000196126 | 6 | HLA-DRB1 | 32546546 | rs113568276 | 6 | 32513127 | A | G | 0.257455 | -0.120501 | 0.0475651 | 0.0112964 | -0.235707 | 0.0189896 | | 2.24E-35 | | 0.511232 | | 0.205958 | | 0.01305676 | | 0.9991782 | | 20 | | 0.1305676 | |
| SSC-A on monocyte | ENSG00000177000 | 1 | MTHFR | 11845780 | rs147124396 | 1 | 11880749 | A | G | 0.0974155 | 0.123104 | 0.0621221 | 0.0475193 | 0.737566 | 0.0508756 | | 1.26E-47 | | 0.166906 | | 0.085009 | | 0.04960102 | | 0.4216706 | | 20 | | 0.59448561 | |
|  | ENSG00000160789 | 1 | LMNA | 156052364 | rs582690 | 1 | 156088256 | G | C | 0.0646123 | -0.247226 | 0.119842 | 0.0391201 | 0.400791 | 0.0498887 | | 9.46E-16 | | -0.616845 | | 0.308715 | | 0.04570491 | | 0.7326794 | | 6 | | 0.59448561 | |
|  | ENSG00000143799 | 1 | PARP1 | 226548392 | rs6661762 | 1 | 226543691 | A | C | 0.16998 | 0.121518 | 0.0535562 | 0.0232691 | 0.0945854 | 0.0170385 | | 2.84E-08 | | 1.28474 | | 0.611692 | | 0.03570133 | | NA | | NA | | 0.59448561 | |
|  | ENSG00000204217 | 2 | BMPR2 | 203241659 | rs35689601 | 2 | 203128816 | G | T | 0.258449 | 0.108033 | 0.0419036 | 0.00993368 | 0.197218 | 0.0239662 | | 1.89E-16 | | 0.547785 | | 0.222657 | | 0.01388535 | | 0.2913144 | | 20 | | 0.326305725 | |
|  | ENSG00000134086 | 3 | VHL | 10182692 | rs412210 | 3 | 10199444 | G | C | 0.699801 | 0.136371 | 0.0460093 | 0.00303683 | 0.115304 | 0.0208619 | | 3.26E-08 | | 1.18271 | | 0.452783 | | 0.008999121 | | 0.5314812 | | 12 | | 0.317219015 | |
|  | ENSG00000170606 | 5 | HSPA4 | 132387654 | rs72801474 | 5 | 132444128 | A | G | 0.0685885 | -0.252321 | 0.0886916 | 0.00444222 | -0.355222 | 0.0411964 | | 6.54E-18 | | 0.710319 | | 0.262918 | | 0.006899135 | | 0.5593649 | | 7 | | 0.317219015 | |
|  | ENSG00000204632 | 6 | HLA-G | 29794744 | rs3130643 | 6 | 30766634 | T | A | 0.854871 | -0.18337 | 0.0604248 | 0.00240791 | 0.489873 | 0.066645 | | 1.97E-13 | | -0.374322 | | 0.133447 | | 0.005031257 | | 0.1374094 | | 20 | | 0.317219015 | |
|  | ENSG00000196126 | 6 | HLA-DRB1 | 32546546 | rs113568276 | 6 | 32513127 | A | G | 0.257455 | -0.120501 | 0.0475651 | 0.0112964 | -0.235707 | 0.0189896 | | 2.24E-35 | | 0.511232 | | 0.205958 | | 0.01305676 | | 0.9991782 | | 20 | | 0.326305725 | |
|  | ENSG00000111424 | 12 | VDR | 48235320 | rs12721375 | 12 | 48282900 | A | G | 0.144135 | -0.226032 | 0.054052 | 2.89E-05 | -0.312205 | 0.0384655 | | 4.80E-16 | | 0.723986 | | 0.194757 | | 0.000201306 | | 0.1024755 | | 6 | | 0.028384118 | |
|  | ENSG00000258839 | 16 | MC1R | 89978527 | rs62052185 | 16 | 89969254 | T | C | 0.0805169 | 0.163348 | 0.0743837 | 0.0280905 | 0.662915 | 0.0568198 | | 1.88E-31 | | 0.246409 | | 0.114177 | | 0.03091837 | | 0.1003539 | | 20 | | 0.59448561 | |
| HLA DR on CD33br HLA DR+ CD14dim | ENSG00000203747 | 1 | FCGR3A | 161511549 | rs74127076 | 1 | 161600744 | T | C | 0.335984 | 0.0667099 | 0.0419791 | 0.112033 | 0.314007 | 0.0373409 | | 4.13E-17 | | 0.212447 | | 0.136055 | | 0.1184094 | | 0.5529845 | | 18 | | 0.3708105 | |
| HLA DR on CD33- HLA DR+ | ENSG00000135828 | 1 | RNASEL | 182542769 | rs12041623 | 1 | 182561633 | C | T | 0.370775 | -0.116285 | 0.0403877 | 0.00398648 | 0.0953317 | 0.0146109 | | 6.81E-11 | | -1.21979 | | 0.46307 | | 0.008434899 | | 0.1226285 | | 3 | | 0.650186492 | |
|  | ENSG00000143799 | 1 | PARP1 | 226548392 | rs6661762 | 1 | 226543691 | A | C | 0.16998 | 0.121518 | 0.0535562 | 0.0232691 | 0.0945854 | 0.0170385 | | 2.84E-08 | | 1.28474 | | 0.611692 | | 0.03570133 | | NA | | NA | | 0.75644237 | |
|  | ENSG00000116030 | 2 | SUMO1 | 203070903 | rs3754931 | 2 | 203103401 | A | G | 0.463221 | -0.0844266 | 0.0406163 | 0.0376504 | -0.0920919 | 0.0151562 | | 1.23E-09 | | 0.916765 | | 0.466135 | | 0.04921344 | | 0.4517225 | | 3 | | 0.75644237 | |
|  | ENSG00000168918 | 2 | INPP5D | 233924677 | rs6715767 | 2 | 234074817 | A | G | 0.432406 | -0.0786346 | 0.0390899 | 0.0442588 | -0.171861 | 0.0212248 | | 5.63E-16 | | 0.457548 | | 0.234365 | | 0.050904 | | 0.4857735 | | 20 | | 0.75644237 | |
|  | ENSG00000134086 | 3 | VHL | 10182692 | rs412210 | 3 | 10199444 | G | C | 0.699801 | 0.136371 | 0.0460093 | 0.00303683 | 0.115304 | 0.0208619 | | 3.26E-08 | | 1.18271 | | 0.452783 | | 0.008999121 | | 0.5314812 | | 12 | | 0.650186492 | |
|  | ENSG00000170606 | 5 | HSPA4 | 132387654 | rs72801474 | 5 | 132444128 | A | G | 0.0685885 | -0.252321 | 0.0886916 | 0.00444222 | -0.355222 | 0.0411964 | | 6.54E-18 | | 0.710319 | | 0.262918 | | 0.006899135 | | 0.5593649 | | 7 | | 0.650186492 | |
|  | ENSG00000113263 | 5 | ITK | 156569944 | rs13355489 | 5 | 156664822 | C | T | 0.138171 | 0.133571 | 0.057389 | 0.0199407 | 0.0728742 | 0.0133178 | | 4.45E-08 | | 1.8329 | | 0.855785 | | 0.03221172 | | 0.8788798 | | 4 | | 0.75644237 | |
|  | ENSG00000204632 | 6 | HLA-G | 29794744 | rs3130643 | 6 | 30766634 | T | A | 0.854871 | -0.18337 | 0.0604248 | 0.00240791 | 0.489873 | 0.066645 | | 1.97E-13 | | -0.374322 | | 0.133447 | | 0.005031257 | | 0.1374094 | | 20 | | 0.650186492 | |
|  | ENSG00000237541 | 6 | HLA-DQA2 | 32709119 | rs9272358 | 6 | 32604538 | A | G | 0.370775 | -0.0823102 | 0.0417888 | 0.0488754 | 1.04878 | 0.0420171 | | 1.63E-137 | | -0.0784819 | | 0.039969 | | 0.04958029 | | 0.2012622 | | 20 | | 0.75644237 | |
|  | ENSG00000106976 | 9 | DNM1 | 130965658 | rs10760548 | 9 | 130965235 | A | G | 0.988072 | -0.296469 | 0.143975 | 0.0394775 | -0.875681 | 0.0944891 | | 1.91E-20 | | 0.338558 | | 0.168425 | | 0.04441552 | | 0.6131758 | | 3 | | 0.75644237 | |
|  | ENSG00000065618 | 10 | COL17A1 | 105791044 | rs2181833 | 10 | 105793369 | A | G | 0.807157 | -0.112166 | 0.0461637 | 0.0151095 | -0.33031 | 0.0305137 | | 2.62E-27 | | 0.339578 | | 0.143236 | | 0.01775167 | | 0.3332134 | | 20 | | 0.75644237 | |
|  | ENSG00000167323 | 11 | STIM1 | 3875757 | rs10160415 | 11 | 3909766 | C | G | 0.328032 | 0.0912236 | 0.0402456 | 0.0234099 | 0.117371 | 0.020542 | | 1.11E-08 | | 0.777224 | | 0.368888 | | 0.03512312 | | 0.9870692 | | 4 | | 0.75644237 | |
|  | ENSG00000159110 | 21 | IFNAR2 | 34602206 | rs1131964 | 21 | 34610487 | C | T | 0.543738 | 0.099566 | 0.038872 | 0.0104258 | -0.0940295 | 0.0171698 | | 4.34E-08 | | -1.05888 | | 0.456384 | | 0.02033254 | | 0.3155707 | | 3 | | 0.75644237 | |
| GWAS: genome-wide association study.  eQTL: expression quantitative trait loci.  SMR: summary-data-based Mendelian randomization.  HEIDI: heterogeneity in dependent instruments. Only genome-wide significant eQTLs (P<5E-8) are taken into the analysis. Only results with a significance level (P-value) less than 0.05 are presented, We focus on SNP-gene combinations with PSMR < genome-wide significance Benjamin Hochberg correction threshold of PFDR＜0.05, and survived after the heterogeneity test (PHEIDI＞0.05). β in GWAS association, regression coefficient of cancer on SNP, log(OR).  SE, standard error.  β in eQTL association, regression coefficient of gene expression on SNP.  β in SMR association, regression coefficient of cancer on gene expression. | | | | | | | | | | | | | | | | | | | | | | | | | | | | | | |

| **Supplementary material Table 7. SMR analysis results from mQTL of immune cells causally associated with multiple myeloma.** | | | | | | | | | | | | | | | | | | | | | | |
| --- | --- | --- | --- | --- | --- | --- | --- | --- | --- | --- | --- | --- | --- | --- | --- | --- | --- | --- | --- | --- | --- | --- |
| **Type of immune cell** | **probeID** | **Probe Chr** | **Gene** | **Probe base pair** | **topSNP** | **SNP Chr** | **SNP base pair** | **Effect allele** | **Other allele** | **Effect allele frequence** | **GWAS association** | | | **mQTL association** | | | **SMR association** | | | **HEIDI Test** | | **BH correction** |
|  |  |  |  |  |  |  |  |  |  |  | **β** | **SE** | **P** | **β** | **SE** | **P** | **β** | **SE** | **P** | **P** | **nsnp** | **P-FDR** |
| CD25hi %CD4+ | cg23707289 | 18 | BCL2 | 60988099 | rs1801018 | 18 | 60985879 | C | T | 0.475149 | 0.0849442 | 0.0392215 | 0.0303298 | -0.582736 | 0.0296123 | 3.28E-86 | -0.145768 | 0.0677122 | 0.0313377 | 0.2510324 | 20 | 0.8682553 |
| DP (CD4+CD8+) %T cell | cg08150742 | 12 | CCNT1 | 49086916 | rs10783278 | 12 | 49079470 | C | T | 0.424453 | -0.162087 | 0.0395612 | 4.18E-05 | -0.484252 | 0.030818 | 1.23E-55 | 0.334716 | 0.0844269 | 7.35E-06 | 0.2853697 | 20 | 0.006999965 |
|  | cg27036740 | 12 | CCNT1 | 49086943 | rs3741629 | 12 | 49054491 | G | C | 0.422465 | -0.158689 | 0.0395203 | 5.94E-05 | -0.329296 | 0.0310513 | 2.83E-26 | 0.481904 | 0.128329 | 1.73195E-05 | 0.5265004 | 20 | 0.008244082 |
|  | cg04814784 | 3 | VHL | 10182561 | rs279545 | 3 | 9972493 | G | A | 0.190855 | -0.153389 | 0.0548819 | 0.00519159 | 1.08727 | 0.0314928 | 3.45E-261 | -0.141077 | 0.0506419 | 5.33993E-05 | 0.7875467 | 20 | 0.016945375 |
|  | cg10045137 | 2 | POMC | 25383940 | rs6545951 | 2 | 25343125 | T | C | 0.0487078 | 0.254232 | 0.078579 | 0.00121484 | -1.71319 | 0.0960368 | 3.53E-71 | -0.148397 | 0.0466153 | 8.45539E-05 | 0.13172 | 20 | 0.020123828 |
|  | cg02757179 | 2 | POMC | 25384809 | rs17039879 | 2 | 25371477 | A | G | 0.0397614 | 0.401755 | 0.137684 | 0.00352338 | -1.4695 | 0.0977966 | 4.95E-51 | -0.273396 | 0.0954447 | 9.98469E-05 | 0.1054922 | 20 | 0.019010842 |
|  | cg05030953 | 6 | HLA-C | 31241000 | rs9264486 | 6 | 31232482 | A | T | 0.122266 | 0.174935 | 0.0522231 | 0.000808816 | 1.04119 | 0.0379504 | 1.03E-165 | 0.168014 | 0.0505296 | 0.000883962 | 0.4960896 | 20 | 0.140255272 |
|  | cg08309069 | 6 | HLA-C | 31240651 | rs9264486 | 6 | 31232482 | A | T | 0.122266 | 0.174935 | 0.0522231 | 0.000808816 | 0.82349 | 0.040246 | 4.76E-93 | 0.212431 | 0.064261 | 0.000947204 | 0.6151317 | 20 | 0.128819771 |
|  | cg10594414 | 2 | HDAC4 | 240298910 | rs2411844 | 2 | 240300954 | C | A | 0.239563 | 0.136338 | 0.0444528 | 0.00216192 | 0.513387 | 0.0394285 | 9.33E-39 | 0.265566 | 0.088957 | 0.002832748 | 0.3362572 | 20 | 0.337097012 |
|  | cg25067162 | 17 | BRCA1 | 41277974 | rs3785546 | 17 | 41216933 | C | T | 0.360835 | -0.122378 | 0.0402184 | 0.00234347 | 0.4659 | 0.034057 | 1.34E-42 | -0.26267 | 0.0884338 | 0.002975605 | 0.4102552 | 20 | 0.338557724 |
|  | cg02716646 | 2 | POMC | 25384293 | rs6751851 | 2 | 25381148 | A | G | 0.0526839 | 0.255123 | 0.0783732 | 0.001133 | -0.720325 | 0.0994187 | 4.31E-13 | -0.354178 | 0.119279 | 0.002984686 | 0.09511481 | 16 | 0.305631846 |
|  | cg23533285 | 6 | HLA-B | 31322348 | rs2523579 | 6 | 31328517 | T | C | 0.270378 | 0.121022 | 0.0401707 | 0.00258929 | 0.523644 | 0.0324833 | 1.83E-58 | 0.231115 | 0.0780419 | 0.003062233 | 0.7468417 | 20 | 0.285066054 |
|  | cg23327859 | 12 | CCNT1 | 49111433 | rs61942054 | 12 | 49078745 | A | G | 0.0337972 | 0.345901 | 0.111412 | 0.00190471 | -0.753418 | 0.0959052 | 3.97E-15 | -0.459109 | 0.159005 | 0.003884533 | 0.869785 | 4 | 0.331480149 |
|  | cg06846259 | 2 | POMC | 25384654 | rs6713396 | 2 | 25384705 | C | T | 0.0387674 | 0.400513 | 0.137893 | 0.00367824 | -1.4602 | 0.0977435 | 1.83E-50 | -0.274286 | 0.0962026 | 0.00435638 | 0.1577411 | 20 | 0.343148702 |
|  | cg26706521 | 6 | HLA-B | 31325085 | rs9266242 | 6 | 31325620 | T | C | 0.270378 | 0.121178 | 0.0423751 | 0.00424121 | 0.782343 | 0.0307905 | 2.03E-142 | 0.154891 | 0.0545063 | 0.004487227 | 0.131786 | 20 | 0.328208603 |
|  | cg23870181 | 6 | HLA-DQA2 | 32713044 | rs75565243 | 6 | 31463379 | A | G | 0.0298211 | 0.261619 | 0.089954 | 0.00363321 | -1.47721 | 0.112803 | 3.49E-39 | -0.177103 | 0.0623782 | 0.004522852 | 0.1991702 | 4 | 0.30876003 |
|  | cg04981410 | 6 | HLA-B | 31325427 | rs2523587 | 6 | 31327400 | T | C | 0.269384 | 0.121373 | 0.0423436 | 0.00415193 | -0.617162 | 0.0298875 | 9.87E-95 | -0.196663 | 0.069268 | 0.004523236 | 0.06962941 | 20 | 0.289487104 |
|  | cg10466124 | 6 | HLA-DRB5 | 32498285 | rs9271406 | 6 | 32587588 | A | G | 0.49006 | 0.111451 | 0.0389445 | 0.0042125 | 0.658387 | 0.0300641 | 2.63E-106 | 0.169279 | 0.0596543 | 0.004544502 | 0.904659 | 20 | 0.273739415 |
|  | cg23221363 | 6 | HLA-DQA2 | 32710933 | rs75565243 | 6 | 31463379 | A | G | 0.0298211 | 0.261619 | 0.089954 | 0.00363321 | -1.4455 | 0.112999 | 1.81E-37 | -0.180989 | 0.0638185 | 0.004568372 | 0.165753 | 4 | 0.259889607 |
|  | cg17554194 | 6 | HLA-B | 31324972 | rs9266242 | 6 | 31325620 | T | C | 0.270378 | 0.121178 | 0.0423751 | 0.00424121 | -0.584951 | 0.0298663 | 2.05E-85 | -0.207159 | 0.0732102 | 0.004659914 | 0.2821688 | 20 | 0.251144839 |
|  | cg18511546 | 6 | HLA-C | 31238388 | rs2523578 | 6 | 31328542 | G | A | 0.201789 | 0.12652 | 0.0449186 | 0.00485277 | 0.884449 | 0.0307999 | 2.41E-181 | 0.14305 | 0.0510308 | 0.005059858 | 0.1777831 | 20 | 0.25906473 |
|  | cg10924779 | 12 | CCNT1 | 49111395 | rs61942054 | 12 | 49078745 | A | G | 0.0337972 | 0.345901 | 0.111412 | 0.00190471 | -0.586091 | 0.0957541 | 9.31E-10 | -0.590183 | 0.21315 | 0.005625178 | 0.8601804 | 3 | 0.274294394 |
|  | cg11728928 | 17 | CCL3 | 34415818 | rs8064426 | 17 | 34819750 | A | G | 0.178926 | 0.128914 | 0.0461039 | 0.00517154 | 0.969384 | 0.0497348 | 1.31E-84 | 0.132985 | 0.0480469 | 0.005643156 | 0.1890169 | 20 | 0.262663261 |
|  | cg17974398 | 6 | HLA-C | 31239324 | rs9266245 | 6 | 31325702 | A | G | 0.201789 | 0.126296 | 0.0449578 | 0.00496649 | -0.516975 | 0.0338716 | 1.35E-52 | -0.244298 | 0.0884239 | 0.005730708 | 0.1115948 | 20 | 0.255141087 |
|  | cg20559215 | 6 | MAPK14 | 36076933 | rs3804452 | 6 | 36076934 | A | G | 0.119284 | 0.152266 | 0.0554191 | 0.00600441 | -1.80927 | 0.0375894 | 0 | -0.0841588 | 0.0306805 | 0.006086751 | 0.1807861 | 20 | 0.259701376 |
|  | cg22758471 | 6 | BACH2 | 90639197 | rs45519933 | 6 | 90639401 | T | C | 0.112326 | 0.142425 | 0.0505449 | 0.00483571 | -0.540792 | 0.0496088 | 1.14E-27 | -0.263364 | 0.0965365 | 0.006369544 | 0.6983941 | 11 | 0.260896522 |
|  | cg23977453 | 3 | VHL | 10182923 | rs279545 | 3 | 9972493 | G | A | 0.190855 | -0.153389 | 0.0548819 | 0.00519159 | 0.434393 | 0.0379475 | 2.43E-30 | -0.353111 | 0.130053 | 0.006624839 | 0.772625 | 20 | 0.260916736 |
|  | cg19393006 | 9 | TSC1 | 135820767 | rs11243940 | 9 | 135821372 | G | A | 0.206759 | -0.150245 | 0.0552056 | 0.00649756 | -0.880058 | 0.0353159 | 4.56E-137 | 0.170722 | 0.0631025 | 0.00682085 | 0.9031904 | 20 | 0.258687052 |
|  | cg02293354 | 6 | HLA-DQA2 | 32711008 | rs9271406 | 6 | 32587588 | A | G | 0.49006 | 0.111451 | 0.0389445 | 0.0042125 | 0.245329 | 0.0322401 | 2.75E-14 | 0.454292 | 0.169599 | 0.007392578 | 0.8912678 | 20 | 0.270357138 |
|  | cg13778567 | 6 | HLA-DQA1 | 32609783 | rs28366319 | 6 | 32561495 | A | G | 0.300199 | -0.123827 | 0.0467008 | 0.00801346 | 1.09606 | 0.0248368 | 0 | -0.112975 | 0.0426847 | 0.008127582 | 0.9999129 | 20 | 0.286987723 |
|  | cg24290947 | 6 | HLA-DMA | 32921805 | rs1050391 | 6 | 32917857 | A | G | 0.0675944 | 0.174445 | 0.0656413 | 0.007871 | -0.993134 | 0.049433 | 8.93E-90 | -0.175651 | 0.0666709 | 0.008423655 | 0.0829875 | 20 | 0.287527424 |
|  | cg01647308 | 2 | HDAC4 | 240302797 | rs2048764 | 2 | 240301377 | T | C | 0.350895 | 0.110128 | 0.0401942 | 0.00614596 | 0.330643 | 0.0346754 | 1.49E-21 | 0.333072 | 0.126483 | 0.008454883 | 0.432714 | 16 | 0.279283877 |
|  | cg14061503 | 9 | TSC1 | 135820653 | rs7874234 | 9 | 135813001 | T | C | 0.207753 | -0.153076 | 0.0531156 | 0.0039523 | -0.238199 | 0.0370003 | 1.21E-10 | 0.642639 | 0.244312 | 0.008528463 | 0.7054209 | 15 | 0.272910816 |
|  | cg19476788 | 18 | NFATC1 | 77172117 | rs304932 | 18 | 77172078 | G | A | 0.392644 | -0.103284 | 0.0391062 | 0.00826342 | 0.769585 | 0.029093 | 3.41E-154 | -0.134207 | 0.0510673 | 0.008587612 | 0.708931 | 20 | 0.266476203 |
|  | cg10123514 | 6 | HLA-DMB | 32904061 | rs145364886 | 6 | 32946979 | T | C | 0.0308151 | 0.7121 | 0.269376 | 0.00820484 | -1.58608 | 0.0798706 | 9.37E-88 | -0.448969 | 0.171336 | 0.008782648 | 0.1410212 | 20 | 0.264512693 |
|  | cg26876664 | 2 | HDAC4 | 240302637 | rs6712730 | 2 | 240302188 | C | G | 0.353877 | 0.110285 | 0.0401775 | 0.00605215 | 0.299707 | 0.0347158 | 5.97E-18 | 0.367976 | 0.140669 | 0.008899243 | 0.566672 | 15 | 0.260366424 |
|  | cg26674160 | 21 | S100B | 48025100 | rs13047696 | 21 | 48027177 | G | A | 0.314115 | 0.10591 | 0.0405795 | 0.00905607 | 0.668764 | 0.0321558 | 4.55E-96 | 0.158367 | 0.0611543 | 0.009607996 | 0.06975897 | 20 | 0.273294108 |
|  | cg08177681 | 2 | HDAC4 | 240302585 | rs2048764 | 2 | 240301377 | T | C | 0.350895 | 0.110128 | 0.0401942 | 0.00614596 | 0.271466 | 0.0346414 | 4.63E-15 | 0.405679 | 0.156853 | 0.009699281 | 0.7356256 | 15 | 0.268434155 |
| CD8dim %T cell | cg18737534 | 17 | RPA1 | 1800702 | rs3744768 | 17 | 1800886 | A | G | 0.217694 | 0.0982855 | 0.0452401 | 0.029816 | 0.216112 | 0.0373994 | 7.54E-09 | 0.45479 | 0.223643 | 0.006419964 | 0.6285651 | 7 | 0.04493972 |
| CD8dim %leukocyte | cg18737534 | 17 | RPA1 | 1800702 | rs3744768 | 17 | 1800886 | A | G | 0.217694 | 0.0982855 | 0.0452401 | 0.029816 | 0.216112 | 0.0373994 | 7.54E-09 | 0.45479 | 0.223643 | 0.006419964 | 0.6285651 | 7 | 0.04959387 |
|  | cg10045137 | 2 | POMC | 25383940 | rs6545951 | 2 | 25343125 | T | C | 0.0487078 | 0.254232 | 0.078579 | 0.00121484 | -1.71319 | 0.0960368 | 3.53E-71 | -0.148397 | 0.0466153 | 8.45539E-05 | 0.13172 | 20 | 0.000573602 |
|  | cg02757179 | 2 | POMC | 25384809 | rs17039879 | 2 | 25371477 | A | G | 0.0397614 | 0.401755 | 0.137684 | 0.00352338 | -1.4695 | 0.0977966 | 4.95E-51 | -0.273396 | 0.0954447 | 9.98469E-05 | 0.1054922 | 20 | 0.000349464 |
| HLA DR+ T cell%T cell | cg05030953 | 6 | HLA-C | 31241000 | rs9264486 | 6 | 31232482 | A | T | 0.122266 | 0.174935 | 0.0522231 | 0.000808816 | 1.04119 | 0.0379504 | 1.03E-165 | 0.168014 | 0.0505296 | 0.000883962 | 0.4960896 | 20 | 0.373601865 |
|  | cg08309069 | 6 | HLA-C | 31240651 | rs9264486 | 6 | 31232482 | A | T | 0.122266 | 0.174935 | 0.0522231 | 0.000808816 | 0.82349 | 0.040246 | 4.76E-93 | 0.212431 | 0.064261 | 0.000947204 | 0.6151317 | 20 | 0.373601865 |
|  | cg08493294 | 2 | DNMT3A | 25500416 | rs56089703 | 2 | 25500289 | A | T | 0.350895 | -0.139876 | 0.0424898 | 0.000994787 | 0.244252 | 0.0336303 | 3.79E-13 | -0.572671 | 0.190994 | 0.002714334 | 0.09203194 | 6 | 0.373601865 |
|  | cg02716646 | 2 | POMC | 25384293 | rs6751851 | 2 | 25381148 | A | G | 0.0526839 | 0.255123 | 0.0783732 | 0.001133 | -0.720325 | 0.0994187 | 4.31E-13 | -0.354178 | 0.119279 | 0.002984686 | 0.09511481 | 16 | 0.373601865 |
|  | cg23533285 | 6 | HLA-B | 31322348 | rs2523579 | 6 | 31328517 | T | C | 0.270378 | 0.121022 | 0.0401707 | 0.00258929 | 0.523644 | 0.0324833 | 1.83E-58 | 0.231115 | 0.0780419 | 0.003062233 | 0.7468417 | 20 | 0.373601865 |
|  | cg06846259 | 2 | POMC | 25384654 | rs6713396 | 2 | 25384705 | C | T | 0.0387674 | 0.400513 | 0.137893 | 0.00367824 | -1.4602 | 0.0977435 | 1.83E-50 | -0.274286 | 0.0962026 | 0.00435638 | 0.1577411 | 20 | 0.373601865 |
|  | cg26706521 | 6 | HLA-B | 31325085 | rs9266242 | 6 | 31325620 | T | C | 0.270378 | 0.121178 | 0.0423751 | 0.00424121 | 0.782343 | 0.0307905 | 2.03E-142 | 0.154891 | 0.0545063 | 0.004487227 | 0.131786 | 20 | 0.373601865 |
|  | cg04981410 | 6 | HLA-B | 31325427 | rs2523587 | 6 | 31327400 | T | C | 0.269384 | 0.121373 | 0.0423436 | 0.00415193 | -0.617162 | 0.0298875 | 9.87E-95 | -0.196663 | 0.069268 | 0.004523236 | 0.06962941 | 20 | 0.373601865 |
|  | cg17554194 | 6 | HLA-B | 31324972 | rs9266242 | 6 | 31325620 | T | C | 0.270378 | 0.121178 | 0.0423751 | 0.00424121 | -0.584951 | 0.0298663 | 2.05E-85 | -0.207159 | 0.0732102 | 0.004659914 | 0.2821688 | 20 | 0.373601865 |
|  | cg18511546 | 6 | HLA-C | 31238388 | rs2523578 | 6 | 31328542 | G | A | 0.201789 | 0.12652 | 0.0449186 | 0.00485277 | 0.884449 | 0.0307999 | 2.41E-181 | 0.14305 | 0.0510308 | 0.005059858 | 0.1777831 | 20 | 0.373601865 |
|  | cg11728928 | 17 | CCL3 | 34415818 | rs8064426 | 17 | 34819750 | A | G | 0.178926 | 0.128914 | 0.0461039 | 0.00517154 | 0.969384 | 0.0497348 | 1.31E-84 | 0.132985 | 0.0480469 | 0.005643156 | 0.1890169 | 20 | 0.373601865 |
|  | cg17974398 | 6 | HLA-C | 31239324 | rs9266245 | 6 | 31325702 | A | G | 0.201789 | 0.126296 | 0.0449578 | 0.00496649 | -0.516975 | 0.0338716 | 1.35E-52 | -0.244298 | 0.0884239 | 0.005730708 | 0.1115948 | 20 | 0.373601865 |
|  | cg20559215 | 6 | MAPK14 | 36076933 | rs3804452 | 6 | 36076934 | A | G | 0.119284 | 0.152266 | 0.0554191 | 0.00600441 | -1.80927 | 0.0375894 | 0 | -0.0841588 | 0.0306805 | 0.006086751 | 0.1807861 | 20 | 0.373601865 |
|  | cg08485187 | 2 | DNMT3A | 25500046 | rs57976122 | 2 | 25506107 | T | C | 0.349901 | -0.121864 | 0.0421512 | 0.00383893 | 0.299696 | 0.0352096 | 1.71E-17 | -0.406625 | 0.148538 | 0.006190586 | 0.06988053 | 9 | 0.373601865 |
|  | cg13778567 | 6 | HLA-DQA1 | 32609783 | rs28366319 | 6 | 32561495 | A | G | 0.300199 | -0.123827 | 0.0467008 | 0.00801346 | 1.09606 | 0.0248368 | 0 | -0.112975 | 0.0426847 | 0.008127582 | 0.9999129 | 20 | 0.467142451 |
|  | cg26674160 | 21 | S100B | 48025100 | rs13047696 | 21 | 48027177 | G | A | 0.314115 | 0.10591 | 0.0405795 | 0.00905607 | 0.668764 | 0.0321558 | 4.55E-96 | 0.158367 | 0.0611543 | 0.009607996 | 0.06975897 | 20 | 0.486555558 |
| CD20 on CD20- CD38- | cg10045137 | 2 | POMC | 25383940 | rs6545951 | 2 | 25343125 | T | C | 0.0487078 | 0.254232 | 0.078579 | 0.00121484 | -1.71319 | 0.0960368 | 3.53E-71 | -0.148397 | 0.0466153 | 8.45539E-05 | 0.13172 | 20 | 0.373601865 |
|  | cg05030953 | 6 | HLA-C | 31241000 | rs9264486 | 6 | 31232482 | A | T | 0.122266 | 0.174935 | 0.0522231 | 0.000808816 | 1.04119 | 0.0379504 | 1.03E-165 | 0.168014 | 0.0505296 | 0.000883962 | 0.4960896 | 20 | 0.373601865 |
|  | cg08309069 | 6 | HLA-C | 31240651 | rs9264486 | 6 | 31232482 | A | T | 0.122266 | 0.174935 | 0.0522231 | 0.000808816 | 0.82349 | 0.040246 | 4.76E-93 | 0.212431 | 0.064261 | 0.000947204 | 0.6151317 | 20 | 0.373601865 |
|  | cg08493294 | 2 | DNMT3A | 25500416 | rs56089703 | 2 | 25500289 | A | T | 0.350895 | -0.139876 | 0.0424898 | 0.000994787 | 0.244252 | 0.0336303 | 3.79E-13 | -0.572671 | 0.190994 | 0.002714334 | 0.09203194 | 6 | 0.373601865 |
|  | cg02716646 | 2 | POMC | 25384293 | rs6751851 | 2 | 25381148 | A | G | 0.0526839 | 0.255123 | 0.0783732 | 0.001133 | -0.720325 | 0.0994187 | 4.31E-13 | -0.354178 | 0.119279 | 0.002984686 | 0.09511481 | 16 | 0.373601865 |
|  | cg23533285 | 6 | HLA-B | 31322348 | rs2523579 | 6 | 31328517 | T | C | 0.270378 | 0.121022 | 0.0401707 | 0.00258929 | 0.523644 | 0.0324833 | 1.83E-58 | 0.231115 | 0.0780419 | 0.003062233 | 0.7468417 | 20 | 0.373601865 |
|  | cg02757179 | 2 | POMC | 25384809 | rs17039879 | 2 | 25371477 | A | G | 0.0397614 | 0.401755 | 0.137684 | 0.00352338 | -1.4695 | 0.0977966 | 4.95E-51 | -0.273396 | 0.0954447 | 9.98469E-05 | 0.1054922 | 20 | 0.373601865 |
|  | cg06846259 | 2 | POMC | 25384654 | rs6713396 | 2 | 25384705 | C | T | 0.0387674 | 0.400513 | 0.137893 | 0.00367824 | -1.4602 | 0.0977435 | 1.83E-50 | -0.274286 | 0.0962026 | 0.00435638 | 0.1577411 | 20 | 0.373601865 |
|  | cg26706521 | 6 | HLA-B | 31325085 | rs9266242 | 6 | 31325620 | T | C | 0.270378 | 0.121178 | 0.0423751 | 0.00424121 | 0.782343 | 0.0307905 | 2.03E-142 | 0.154891 | 0.0545063 | 0.004487227 | 0.131786 | 20 | 0.373601865 |
|  | cg04981410 | 6 | HLA-B | 31325427 | rs2523587 | 6 | 31327400 | T | C | 0.269384 | 0.121373 | 0.0423436 | 0.00415193 | -0.617162 | 0.0298875 | 9.87E-95 | -0.196663 | 0.069268 | 0.004523236 | 0.06962941 | 20 | 0.373601865 |
|  | cg17554194 | 6 | HLA-B | 31324972 | rs9266242 | 6 | 31325620 | T | C | 0.270378 | 0.121178 | 0.0423751 | 0.00424121 | -0.584951 | 0.0298663 | 2.05E-85 | -0.207159 | 0.0732102 | 0.004659914 | 0.2821688 | 20 | 0.373601865 |
|  | cg18511546 | 6 | HLA-C | 31238388 | rs2523578 | 6 | 31328542 | G | A | 0.201789 | 0.12652 | 0.0449186 | 0.00485277 | 0.884449 | 0.0307999 | 2.41E-181 | 0.14305 | 0.0510308 | 0.005059858 | 0.1777831 | 20 | 0.373601865 |
|  | cg11728928 | 17 | CCL3 | 34415818 | rs8064426 | 17 | 34819750 | A | G | 0.178926 | 0.128914 | 0.0461039 | 0.00517154 | 0.969384 | 0.0497348 | 1.31E-84 | 0.132985 | 0.0480469 | 0.005643156 | 0.1890169 | 20 | 0.373601865 |
|  | cg17974398 | 6 | HLA-C | 31239324 | rs9266245 | 6 | 31325702 | A | G | 0.201789 | 0.126296 | 0.0449578 | 0.00496649 | -0.516975 | 0.0338716 | 1.35E-52 | -0.244298 | 0.0884239 | 0.005730708 | 0.1115948 | 20 | 0.373601865 |
|  | cg20559215 | 6 | MAPK14 | 36076933 | rs3804452 | 6 | 36076934 | A | G | 0.119284 | 0.152266 | 0.0554191 | 0.00600441 | -1.80927 | 0.0375894 | 0 | -0.0841588 | 0.0306805 | 0.006086751 | 0.1807861 | 20 | 0.373601865 |
|  | cg08485187 | 2 | DNMT3A | 25500046 | rs57976122 | 2 | 25506107 | T | C | 0.349901 | -0.121864 | 0.0421512 | 0.00383893 | 0.299696 | 0.0352096 | 1.71E-17 | -0.406625 | 0.148538 | 0.006190586 | 0.06988053 | 9 | 0.373601865 |
|  | cg13778567 | 6 | HLA-DQA1 | 32609783 | rs28366319 | 6 | 32561495 | A | G | 0.300199 | -0.123827 | 0.0467008 | 0.00801346 | 1.09606 | 0.0248368 | 0 | -0.112975 | 0.0426847 | 0.008127582 | 0.9999129 | 20 | 0.467142451 |
|  | cg26674160 | 21 | S100B | 48025100 | rs13047696 | 21 | 48027177 | G | A | 0.314115 | 0.10591 | 0.0405795 | 0.00905607 | 0.668764 | 0.0321558 | 4.55E-96 | 0.158367 | 0.0611543 | 0.009607996 | 0.06975897 | 20 | 0.486555558 |
| CD25 on IgD- CD38br | cg10045137 | 2 | POMC | 25383940 | rs6545951 | 2 | 25343125 | T | C | 0.0487078 | 0.254232 | 0.078579 | 0.00121484 | -1.71319 | 0.0960368 | 3.53E-71 | -0.148397 | 0.0466153 | 8.45539E-05 | 0.13172 | 20 | 0.013105855 |
|  | cg02757179 | 2 | POMC | 25384809 | rs17039879 | 2 | 25371477 | A | G | 0.0397614 | 0.401755 | 0.137684 | 0.00352338 | -1.4695 | 0.0977966 | 4.95E-51 | -0.273396 | 0.0954447 | 9.98469E-05 | 0.1054922 | 20 | 0.013105855 |
|  | cg02714932 | 12 | VDR | 48287192 | rs4237855 | 12 | 48287203 | G | A | 0.363817 | -0.0930141 | 0.0402836 | 0.0209445 | -0.432601 | 0.0334895 | 3.59E-38 | 0.215011 | 0.0945955 | 0.02302884 | 0.5670383 | 20 | 0.9142173 |
|  | cg02716646 | 2 | POMC | 25384293 | rs6751851 | 2 | 25381148 | A | G | 0.0526839 | 0.255123 | 0.0783732 | 0.001133 | -0.720325 | 0.0994187 | 4.31E-13 | -0.354178 | 0.119279 | 0.002984686 | 0.09511481 | 16 | 0.46395447 |
|  | cg06846259 | 2 | POMC | 25384654 | rs6713396 | 2 | 25384705 | C | T | 0.0387674 | 0.400513 | 0.137893 | 0.00367824 | -1.4602 | 0.0977435 | 1.83E-50 | -0.274286 | 0.0962026 | 0.00435638 | 0.1577411 | 20 | 0.46395447 |
|  | cg00310201 | 2 | ZAP70 | 98351994 | rs6724539 | 2 | 98343258 | G | A | 0.506958 | 0.104847 | 0.0407153 | 0.0100205 | 0.724345 | 0.0300655 | 3.02E-128 | 0.144747 | 0.05653 | 0.01045096 | 0.6410191 | 20 | 0.890421792 |
|  | cg08464513 | 16 | MAPK3 | 30136024 | rs8061772 | 16 | 30119525 | C | A | 0.142147 | -0.127963 | 0.0557226 | 0.0216516 | -0.553878 | 0.0448914 | 5.64E-35 | 0.231031 | 0.102332 | 0.02396705 | 0.4804967 | 20 | 0.9142173 |
|  | cg23707289 | 18 | BCL2 | 60988099 | rs1801018 | 18 | 60985879 | C | T | 0.475149 | 0.0849442 | 0.0392215 | 0.0303298 | -0.582736 | 0.0296123 | 3.28E-86 | -0.145768 | 0.0677122 | 0.0313377 | 0.2510324 | 20 | 0.9142173 |
|  | cg27083787 | 2 | IL1A | 113543245 | rs7583261 | 2 | 113548454 | G | C | 0.287276 | 0.0934142 | 0.0418484 | 0.0256012 | 0.239631 | 0.03473 | 5.21E-12 | 0.389825 | 0.183548 | 0.03368459 | 0.02959982 | 9 | 0.9142173 |
|  | cg03228353 | 1 | FCGR3A | 161520094 | rs7539053 | 1 | 161659845 | T | C | 0.218688 | 0.0989426 | 0.0454841 | 0.029606 | -0.238081 | 0.0387771 | 8.27E-10 | -0.415584 | 0.202681 | 0.04032242 | 0.7862339 | 7 | 0.9142173 |
|  | cg05772104 | 2 | ZAP70 | 98340425 | rs5865 | 2 | 98373006 | C | T | 0.346918 | -0.0902723 | 0.0429829 | 0.0357117 | 0.282836 | 0.0337979 | 5.84E-17 | -0.319168 | 0.156684 | 0.04164793 | 0.1268268 | 20 | 0.9142173 |
|  | cg11304234 | 11 | IL18 | 112034801 | rs10891343 | 11 | 112080384 | T | C | 0.487078 | 0.0829885 | 0.0392725 | 0.0345884 | 0.211074 | 0.0321845 | 5.44E-11 | 0.393173 | 0.19548 | 0.04429219 | 0.5590909 | 17 | 0.9142173 |
|  | cg26534425 | 11 | IL18 | 112034925 | rs10891343 | 11 | 112080384 | T | C | 0.487078 | 0.0829885 | 0.0392725 | 0.0345884 | 0.195636 | 0.0325873 | 1.93E-09 | 0.424199 | 0.212815 | 0.04623181 | 0.7409206 | 10 | 0.9142173 |
|  | cg04230403 | 10 | FAS | 90750176 | rs6586167 | 10 | 90756146 | T | G | 0.532803 | 0.0817381 | 0.0391911 | 0.0370126 | -0.204623 | 0.0318083 | 1.25E-10 | -0.399457 | 0.201343 | 0.04725964 | 0.7591863 | 12 | 0.9142173 |
|  | cg14712983 | 1 | TNFRSF8 | 12184541 | rs646249 | 1 | 12180124 | G | A | 0.4334 | 0.0801536 | 0.0403891 | 0.0471965 | -0.589889 | 0.0313834 | 8.11E-79 | -0.135879 | 0.0688496 | 0.04843114 | 0.4094712 | 11 | 0.9142173 |
| CD45 on CD4+ | cg05030953 | 6 | HLA-C | 31241000 | rs9264486 | 6 | 31232482 | A | T | 0.122266 | 0.174935 | 0.0522231 | 0.000808816 | 1.04119 | 0.0379504 | 1.03E-165 | 0.168014 | 0.0505296 | 0.000883962 | 0.4960896 | 20 | 0.643151652 |
|  | cg08309069 | 6 | HLA-C | 31240651 | rs9264486 | 6 | 31232482 | A | T | 0.122266 | 0.174935 | 0.0522231 | 0.000808816 | 0.82349 | 0.040246 | 4.76E-93 | 0.212431 | 0.064261 | 0.000947204 | 0.6151317 | 20 | 0.643151652 |
|  | cg10594414 | 2 | HDAC4 | 240298910 | rs2411844 | 2 | 240300954 | C | A | 0.239563 | 0.136338 | 0.0444528 | 0.00216192 | 0.513387 | 0.0394285 | 9.33E-39 | 0.265566 | 0.088957 | 0.002832748 | 0.3362572 | 20 | 0.715184786 |
|  | cg23533285 | 6 | HLA-B | 31322348 | rs2523579 | 6 | 31328517 | T | C | 0.270378 | 0.121022 | 0.0401707 | 0.00258929 | 0.523644 | 0.0324833 | 1.83E-58 | 0.231115 | 0.0780419 | 0.003062233 | 0.7468417 | 20 | 0.715184786 |
|  | cg21302796 | 2 | DYSF | 71913841 | rs79623654 | 2 | 71923487 | A | G | 0.0149105 | -0.963906 | 0.302442 | 0.00143714 | 1.15627 | 0.156147 | 1.31E-13 | -0.833634 | 0.284764 | 0.003417527 | 0.1425319 | 16 | 0.715184786 |
|  | cg26706521 | 6 | HLA-B | 31325085 | rs9266242 | 6 | 31325620 | T | C | 0.270378 | 0.121178 | 0.0423751 | 0.00424121 | 0.782343 | 0.0307905 | 2.03E-142 | 0.154891 | 0.0545063 | 0.004487227 | 0.131786 | 20 | 0.715184786 |
|  | cg23870181 | 6 | HLA-DQA2 | 32713044 | rs75565243 | 6 | 31463379 | A | G | 0.0298211 | 0.261619 | 0.089954 | 0.00363321 | -1.47721 | 0.112803 | 3.49E-39 | -0.177103 | 0.0623782 | 0.004522852 | 0.1991702 | 4 | 0.715184786 |
|  | cg04981410 | 6 | HLA-B | 31325427 | rs2523587 | 6 | 31327400 | T | C | 0.269384 | 0.121373 | 0.0423436 | 0.00415193 | -0.617162 | 0.0298875 | 9.87E-95 | -0.196663 | 0.069268 | 0.004523236 | 0.06962941 | 20 | 0.715184786 |
|  | cg10466124 | 6 | HLA-DRB5 | 32498285 | rs9271406 | 6 | 32587588 | A | G | 0.49006 | 0.111451 | 0.0389445 | 0.0042125 | 0.658387 | 0.0300641 | 2.63E-106 | 0.169279 | 0.0596543 | 0.004544502 | 0.904659 | 20 | 0.715184786 |
|  | cg23221363 | 6 | HLA-DQA2 | 32710933 | rs75565243 | 6 | 31463379 | A | G | 0.0298211 | 0.261619 | 0.089954 | 0.00363321 | -1.4455 | 0.112999 | 1.81E-37 | -0.180989 | 0.0638185 | 0.004568372 | 0.165753 | 4 | 0.715184786 |
|  | cg17554194 | 6 | HLA-B | 31324972 | rs9266242 | 6 | 31325620 | T | C | 0.270378 | 0.121178 | 0.0423751 | 0.00424121 | -0.584951 | 0.0298663 | 2.05E-85 | -0.207159 | 0.0732102 | 0.004659914 | 0.2821688 | 20 | 0.715184786 |
|  | cg18511546 | 6 | HLA-C | 31238388 | rs2523578 | 6 | 31328542 | G | A | 0.201789 | 0.12652 | 0.0449186 | 0.00485277 | 0.884449 | 0.0307999 | 2.41E-181 | 0.14305 | 0.0510308 | 0.005059858 | 0.1777831 | 20 | 0.715184786 |
|  | cg11728928 | 17 | CCL3 | 34415818 | rs8064426 | 17 | 34819750 | A | G | 0.178926 | 0.128914 | 0.0461039 | 0.00517154 | 0.969384 | 0.0497348 | 1.31E-84 | 0.132985 | 0.0480469 | 0.005643156 | 0.1890169 | 20 | 0.715184786 |
|  | cg17974398 | 6 | HLA-C | 31239324 | rs9266245 | 6 | 31325702 | A | G | 0.201789 | 0.126296 | 0.0449578 | 0.00496649 | -0.516975 | 0.0338716 | 1.35E-52 | -0.244298 | 0.0884239 | 0.005730708 | 0.1115948 | 20 | 0.715184786 |
|  | cg20559215 | 6 | MAPK14 | 36076933 | rs3804452 | 6 | 36076934 | A | G | 0.119284 | 0.152266 | 0.0554191 | 0.00600441 | -1.80927 | 0.0375894 | 0 | -0.0841588 | 0.0306805 | 0.006086751 | 0.1807861 | 20 | 0.715184786 |
|  | cg02293354 | 6 | HLA-DQA2 | 32711008 | rs9271406 | 6 | 32587588 | A | G | 0.49006 | 0.111451 | 0.0389445 | 0.0042125 | 0.245329 | 0.0322401 | 2.75E-14 | 0.454292 | 0.169599 | 0.007392578 | 0.8912678 | 20 | 0.715184786 |
|  | cg13778567 | 6 | HLA-DQA1 | 32609783 | rs28366319 | 6 | 32561495 | A | G | 0.300199 | -0.123827 | 0.0467008 | 0.00801346 | 1.09606 | 0.0248368 | 0 | -0.112975 | 0.0426847 | 0.008127582 | 0.9999129 | 20 | 0.715184786 |
|  | cg01647308 | 2 | HDAC4 | 240302797 | rs2048764 | 2 | 240301377 | T | C | 0.350895 | 0.110128 | 0.0401942 | 0.00614596 | 0.330643 | 0.0346754 | 1.49E-21 | 0.333072 | 0.126483 | 0.008454883 | 0.432714 | 16 | 0.715184786 |
|  | cg26876664 | 2 | HDAC4 | 240302637 | rs6712730 | 2 | 240302188 | C | G | 0.353877 | 0.110285 | 0.0401775 | 0.00605215 | 0.299707 | 0.0347158 | 5.97E-18 | 0.367976 | 0.140669 | 0.008899243 | 0.566672 | 15 | 0.715184786 |
|  | cg26674160 | 21 | S100B | 48025100 | rs13047696 | 21 | 48027177 | G | A | 0.314115 | 0.10591 | 0.0405795 | 0.00905607 | 0.668764 | 0.0321558 | 4.55E-96 | 0.158367 | 0.0611543 | 0.009607996 | 0.06975897 | 20 | 0.715184786 |
|  | cg08177681 | 2 | HDAC4 | 240302585 | rs2048764 | 2 | 240301377 | T | C | 0.350895 | 0.110128 | 0.0401942 | 0.00614596 | 0.271466 | 0.0346414 | 4.63E-15 | 0.405679 | 0.156853 | 0.009699281 | 0.7356256 | 15 | 0.715184786 |
| CD4 on naive CD4+ | cg08150742 | 12 | CCNT1 | 49086916 | rs10783278 | 12 | 49079470 | C | T | 0.424453 | -0.162087 | 0.0395612 | 4.18E-05 | -0.484252 | 0.030818 | 1.23E-55 | 0.334716 | 0.0844269 | 7.35E-06 | 0.2853697 | 20 | 0.023376948 |
|  | cg10045137 | 2 | POMC | 25383940 | rs6545951 | 2 | 25343125 | T | C | 0.0487078 | 0.254232 | 0.078579 | 0.00121484 | -1.71319 | 0.0960368 | 3.53E-71 | -0.148397 | 0.0466153 | 8.45539E-05 | 0.13172 | 20 | 0.033034887 |
|  | cg27036740 | 12 | CCNT1 | 49086943 | rs3741629 | 12 | 49054491 | G | C | 0.422465 | -0.158689 | 0.0395203 | 5.94E-05 | -0.329296 | 0.0310513 | 2.83E-26 | 0.481904 | 0.128329 | 0.000173195 | 0.5265004 | 20 | 0.043376948 |
|  | cg05030953 | 6 | HLA-C | 31241000 | rs9264486 | 6 | 31232482 | A | T | 0.122266 | 0.174935 | 0.0522231 | 0.000808816 | 1.04119 | 0.0379504 | 1.03E-165 | 0.168014 | 0.0505296 | 0.000883962 | 0.4960896 | 20 | 0.240779308 |
|  | cg08309069 | 6 | HLA-C | 31240651 | rs9264486 | 6 | 31232482 | A | T | 0.122266 | 0.174935 | 0.0522231 | 0.000808816 | 0.82349 | 0.040246 | 4.76E-93 | 0.212431 | 0.064261 | 0.000947204 | 0.6151317 | 20 | 0.240779308 |
|  | cg25067162 | 17 | BRCA1 | 41277974 | rs3785546 | 17 | 41216933 | C | T | 0.360835 | -0.122378 | 0.0402184 | 0.00234347 | 0.4659 | 0.034057 | 1.34E-42 | -0.26267 | 0.0884338 | 0.002975605 | 0.4102552 | 20 | 0.303488745 |
|  | cg02716646 | 2 | POMC | 25384293 | rs6751851 | 2 | 25381148 | A | G | 0.0526839 | 0.255123 | 0.0783732 | 0.001133 | -0.720325 | 0.0994187 | 4.31E-13 | -0.354178 | 0.119279 | 0.002984686 | 0.09511481 | 16 | 0.303488745 |
|  | cg23533285 | 6 | HLA-B | 31322348 | rs2523579 | 6 | 31328517 | T | C | 0.270378 | 0.121022 | 0.0401707 | 0.00258929 | 0.523644 | 0.0324833 | 1.83E-58 | 0.231115 | 0.0780419 | 0.003062233 | 0.7468417 | 20 | 0.303488745 |
|  | cg23327859 | 12 | CCNT1 | 49111433 | rs61942054 | 12 | 49078745 | A | G | 0.0337972 | 0.345901 | 0.111412 | 0.00190471 | -0.753418 | 0.0959052 | 3.97E-15 | -0.459109 | 0.159005 | 0.003884533 | 0.869785 | 4 | 0.303488745 |
|  | cg02757179 | 2 | POMC | 25384809 | rs17039879 | 2 | 25371477 | A | G | 0.0397614 | 0.401755 | 0.137684 | 0.00352338 | -1.4695 | 0.0977966 | 4.95E-51 | -0.273396 | 0.0954447 | 9.98469E-05 | 0.1054922 | 20 | 0.303488745 |
|  | cg06846259 | 2 | POMC | 25384654 | rs6713396 | 2 | 25384705 | C | T | 0.0387674 | 0.400513 | 0.137893 | 0.00367824 | -1.4602 | 0.0977435 | 1.83E-50 | -0.274286 | 0.0962026 | 0.00435638 | 0.1577411 | 20 | 0.303488745 |
|  | cg26706521 | 6 | HLA-B | 31325085 | rs9266242 | 6 | 31325620 | T | C | 0.270378 | 0.121178 | 0.0423751 | 0.00424121 | 0.782343 | 0.0307905 | 2.03E-142 | 0.154891 | 0.0545063 | 0.004487227 | 0.131786 | 20 | 0.303488745 |
|  | cg23870181 | 6 | HLA-DQA2 | 32713044 | rs75565243 | 6 | 31463379 | A | G | 0.0298211 | 0.261619 | 0.089954 | 0.00363321 | -1.47721 | 0.112803 | 3.49E-39 | -0.177103 | 0.0623782 | 0.004522852 | 0.1991702 | 4 | 0.303488745 |
|  | cg04981410 | 6 | HLA-B | 31325427 | rs2523587 | 6 | 31327400 | T | C | 0.269384 | 0.121373 | 0.0423436 | 0.00415193 | -0.617162 | 0.0298875 | 9.87E-95 | -0.196663 | 0.069268 | 0.004523236 | 0.06962941 | 20 | 0.303488745 |
|  | cg10466124 | 6 | HLA-DRB5 | 32498285 | rs9271406 | 6 | 32587588 | A | G | 0.49006 | 0.111451 | 0.0389445 | 0.0042125 | 0.658387 | 0.0300641 | 2.63E-106 | 0.169279 | 0.0596543 | 0.004544502 | 0.904659 | 20 | 0.303488745 |
|  | cg23221363 | 6 | HLA-DQA2 | 32710933 | rs75565243 | 6 | 31463379 | A | G | 0.0298211 | 0.261619 | 0.089954 | 0.00363321 | -1.4455 | 0.112999 | 1.81E-37 | -0.180989 | 0.0638185 | 0.004568372 | 0.165753 | 4 | 0.303488745 |
|  | cg17554194 | 6 | HLA-B | 31324972 | rs9266242 | 6 | 31325620 | T | C | 0.270378 | 0.121178 | 0.0423751 | 0.00424121 | -0.584951 | 0.0298663 | 2.05E-85 | -0.207159 | 0.0732102 | 0.004659914 | 0.2821688 | 20 | 0.303488745 |
|  | cg21375506 | 17 | PRKCA | 64470354 | rs3859214 | 17 | 64460808 | T | G | 0.436382 | -0.116326 | 0.0388946 | 0.0027824 | 0.276729 | 0.0325091 | 1.70E-17 | -0.420361 | 0.148974 | 0.00477692 | 0.6111445 | 20 | 0.303488745 |
|  | cg18511546 | 6 | HLA-C | 31238388 | rs2523578 | 6 | 31328542 | G | A | 0.201789 | 0.12652 | 0.0449186 | 0.00485277 | 0.884449 | 0.0307999 | 2.41E-181 | 0.14305 | 0.0510308 | 0.005059858 | 0.1777831 | 20 | 0.303488745 |
|  | cg10924779 | 12 | CCNT1 | 49111395 | rs61942054 | 12 | 49078745 | A | G | 0.0337972 | 0.345901 | 0.111412 | 0.00190471 | -0.586091 | 0.0957541 | 9.31E-10 | -0.590183 | 0.21315 | 0.005625178 | 0.8601804 | 3 | 0.303488745 |
|  | cg11728928 | 17 | CCL3 | 34415818 | rs8064426 | 17 | 34819750 | A | G | 0.178926 | 0.128914 | 0.0461039 | 0.00517154 | 0.969384 | 0.0497348 | 1.31E-84 | 0.132985 | 0.0480469 | 0.005643156 | 0.1890169 | 20 | 0.303488745 |
|  | cg17974398 | 6 | HLA-C | 31239324 | rs9266245 | 6 | 31325702 | A | G | 0.201789 | 0.126296 | 0.0449578 | 0.00496649 | -0.516975 | 0.0338716 | 1.35E-52 | -0.244298 | 0.0884239 | 0.005730708 | 0.1115948 | 20 | 0.303488745 |
|  | cg20559215 | 6 | MAPK14 | 36076933 | rs3804452 | 6 | 36076934 | A | G | 0.119284 | 0.152266 | 0.0554191 | 0.00600441 | -1.80927 | 0.0375894 | 0 | -0.0841588 | 0.0306805 | 0.006086751 | 0.1807861 | 20 | 0.309450421 |
|  | cg22758471 | 6 | BACH2 | 90639197 | rs45519933 | 6 | 90639401 | T | C | 0.112326 | 0.142425 | 0.0505449 | 0.00483571 | -0.540792 | 0.0496088 | 1.14E-27 | -0.263364 | 0.0965365 | 0.006369544 | 0.6983941 | 11 | 0.311372709 |
|  | cg02293354 | 6 | HLA-DQA2 | 32711008 | rs9271406 | 6 | 32587588 | A | G | 0.49006 | 0.111451 | 0.0389445 | 0.0042125 | 0.245329 | 0.0322401 | 2.75E-14 | 0.454292 | 0.169599 | 0.007392578 | 0.8912678 | 20 | 0.347998764 |
|  | cg13778567 | 6 | HLA-DQA1 | 32609783 | rs28366319 | 6 | 32561495 | A | G | 0.300199 | -0.123827 | 0.0467008 | 0.00801346 | 1.09606 | 0.0248368 | 0 | -0.112975 | 0.0426847 | 0.008127582 | 0.9999129 | 20 | 0.368934169 |
|  | cg26674160 | 21 | S100B | 48025100 | rs13047696 | 21 | 48027177 | G | A | 0.314115 | 0.10591 | 0.0405795 | 0.00905607 | 0.668764 | 0.0321558 | 4.55E-96 | 0.158367 | 0.0611543 | 0.009607996 | 0.06975897 | 20 | 0.395245049 |
| CD39 on granulocyte | cg18511546 | 6 | HLA-C | 31238388 | rs2523578 | 6 | 31328542 | G | A | 0.201789 | 0.12652 | 0.0449186 | 0.00485277 | 0.884449 | 0.0307999 | 2.41E-181 | 0.14305 | 0.0510308 | 0.005059858 | 0.1777831 | 20 | 0.445562547 |
|  | cg17974398 | 6 | HLA-C | 31239324 | rs9266245 | 6 | 31325702 | A | G | 0.201789 | 0.126296 | 0.0449578 | 0.00496649 | -0.516975 | 0.0338716 | 1.35E-52 | -0.244298 | 0.0884239 | 0.005730708 | 0.1115948 | 20 | 0.445562547 |
|  | cg08309069 | 6 | HLA-C | 31240651 | rs9264486 | 6 | 31232482 | A | T | 0.122266 | 0.174935 | 0.0522231 | 0.000808816 | 0.82349 | 0.040246 | 4.76E-93 | 0.212431 | 0.064261 | 0.000947204 | 0.6151317 | 20 | 0.147290253 |
|  | cg05030953 | 6 | HLA-C | 31241000 | rs9264486 | 6 | 31232482 | A | T | 0.122266 | 0.174935 | 0.0522231 | 0.000808816 | 1.04119 | 0.0379504 | 1.03E-165 | 0.168014 | 0.0505296 | 0.000883962 | 0.4960896 | 20 | 0.147290253 |
| CD45 on Gr MDSC | cg03228353 | 1 | FCGR3A | 161520094 | rs7539053 | 1 | 161659845 | T | C | 0.218688 | 0.0989426 | 0.0454841 | 0.029606 | -0.238081 | 0.0387771 | 8.27E-10 | -0.415584 | 0.202681 | 0.04032242 | 0.7862339 | 7 | 0.915654428 |
|  | cg27083787 | 2 | IL1A | 113543245 | rs7583261 | 2 | 113548454 | G | C | 0.287276 | 0.0934142 | 0.0418484 | 0.0256012 | 0.239631 | 0.03473 | 5.21E-12 | 0.389825 | 0.183548 | 0.03368459 | 0.02959982 | 9 | 0.915654428 |
|  | cg06204447 | 6 | HLA-DRB1 | 32546665 | rs9271464 | 6 | 32588544 | T | A | 0.284294 | -0.115759 | 0.0453606 | 0.0107115 | -1.04665 | 0.0271458 | 0 | 0.1106 | 0.0434337 | 0.01088401 | 0.9070743 | 20 | 0.915654428 |
|  | cg10632894 | 6 | HLA-DRB1 | 32552453 | rs9271413 | 6 | 32587768 | G | A | 0.12326 | 0.124654 | 0.0563867 | 0.0270564 | -1.45045 | 0.0318553 | 0 | -0.0859416 | 0.0389211 | 0.02723747 | 0.2498808 | 20 | 0.915654428 |
|  | cg20559215 | 6 | MAPK14 | 36076933 | rs3804452 | 6 | 36076934 | A | G | 0.119284 | 0.152266 | 0.0554191 | 0.00600441 | -1.80927 | 0.0375894 | 0 | -0.0841588 | 0.0306805 | 0.006086751 | 0.1807861 | 20 | 0.915654428 |
|  | cg02407308 | 9 | NOTCH1 | 139400599 | rs7870145 | 9 | 139401565 | G | A | 0.117296 | -0.177033 | 0.0842908 | 0.0357059 | 0.331026 | 0.0504581 | 5.37E-11 | -0.534801 | 0.267366 | 0.04547214 | 0.04586566 | 11 | 0.915654428 |
|  | cg14225168 | 9 | NOTCH1 | 139403827 | rs3124599 | 9 | 139403770 | A | G | 0.0864811 | 0.140564 | 0.0602737 | 0.0196956 | 0.623999 | 0.0772181 | 6.42E-16 | 0.225263 | 0.100535 | 0.02504841 | 0.1928241 | 5 | 0.915654428 |
|  | cg14065526 | 9 | NOTCH1 | 139406352 | rs3124599 | 9 | 139403770 | A | G | 0.0864811 | 0.140564 | 0.0602737 | 0.0196956 | -1.11347 | 0.0778659 | 2.19E-46 | -0.12624 | 0.0548466 | 0.02135282 | NA | NA | 0.915654428 |
|  | cg23084016 | 10 | ALOX5 | 45916904 | rs6593486 | 10 | 45916913 | C | G | 0.276342 | 0.097945 | 0.0468856 | 0.0367062 | 0.8614 | 0.0327359 | 1.34E-152 | 0.113704 | 0.0546008 | 0.03729968 | 0.06377285 | 20 | 0.915654428 |
|  | cg24302529 | 10 | ALOX5 | 45923083 | rs3780901 | 10 | 45917376 | C | T | 0.312127 | 0.104912 | 0.045288 | 0.0205282 | -0.584206 | 0.0338353 | 8.46E-67 | -0.17958 | 0.0782152 | 0.02167707 | 0.05369354 | 20 | 0.915654428 |
|  | cg10909790 | 10 | ALOX5 | 45925455 | rs7080713 | 10 | 45917117 | T | C | 0.308151 | 0.110771 | 0.0454651 | 0.0148341 | 0.203395 | 0.035296 | 8.29E-09 | 0.54461 | 0.242689 | 0.02482819 | 0.0626391 | 5 | 0.915654428 |
|  | cg11304234 | 11 | IL18 | 112034801 | rs10891343 | 11 | 112080384 | T | C | 0.487078 | 0.0829885 | 0.0392725 | 0.0345884 | 0.211074 | 0.0321845 | 5.44E-11 | 0.393173 | 0.19548 | 0.04429219 | 0.5590909 | 17 | 0.915654428 |
|  | cg26534425 | 11 | IL18 | 112034925 | rs10891343 | 11 | 112080384 | T | C | 0.487078 | 0.0829885 | 0.0392725 | 0.0345884 | 0.195636 | 0.0325873 | 1.93E-09 | 0.424199 | 0.212815 | 0.04623181 | 0.7409206 | 10 | 0.915654428 |
|  | cg22018815 | 17 | ERBB2 | 37879654 | rs907087 | 17 | 37828787 | G | A | 0.311133 | 0.108291 | 0.0423811 | 0.0106133 | 0.300817 | 0.0354973 | 2.36E-17 | 0.35999 | 0.147152 | 0.01442962 | 0.2018412 | 20 | 0.915654428 |
|  | cg09149648 | 20 | MMP9 | 44642370 | rs6094239 | 20 | 44653549 | G | A | 0.0467197 | -0.214555 | 0.0785388 | 0.00629825 | 0.472021 | 0.0824201 | 1.02E-08 | -0.454545 | 0.184349 | 0.01367527 | 0.3980971 | 7 | 0.915654428 |
| SSC-A on monocyte | cg04814784 | 3 | VHL | 10182561 | rs279545 | 3 | 9972493 | G | A | 0.190855 | -0.153389 | 0.0548819 | 0.00519159 | 1.08727 | 0.0314928 | 3.45E-261 | -0.141077 | 0.0506419 | 5.33993E-05 | 0.7875467 | 20 | 0.028515173 |
|  | cg10045137 | 2 | POMC | 25383940 | rs6545951 | 2 | 25343125 | T | C | 0.0487078 | 0.254232 | 0.078579 | 0.00121484 | -1.71319 | 0.0960368 | 3.53E-71 | -0.148397 | 0.0466153 | 8.45539E-05 | 0.13172 | 20 | 0.028515173 |
|  | cg05030953 | 6 | HLA-C | 31241000 | rs9264486 | 6 | 31232482 | A | T | 0.122266 | 0.174935 | 0.0522231 | 0.000808816 | 1.04119 | 0.0379504 | 1.03E-165 | 0.168014 | 0.0505296 | 0.000883962 | 0.4960896 | 20 | 0.472230432 |
|  | cg08309069 | 6 | HLA-C | 31240651 | rs9264486 | 6 | 31232482 | A | T | 0.122266 | 0.174935 | 0.0522231 | 0.000808816 | 0.82349 | 0.040246 | 4.76E-93 | 0.212431 | 0.064261 | 0.000947204 | 0.6151317 | 20 | 0.472230432 |
|  | cg15343510 | 6 | AIF1 | 31582285 | rs9267576 | 6 | 31812038 | T | G | 0.107356 | 0.163714 | 0.0545694 | 0.0026988 | -1.36004 | 0.0362058 | 8.278467e-309 | -0.120374 | 0.0402511 | 0.002784449 | 0.6284097 | 20 | 0.472230432 |
|  | cg25067162 | 17 | BRCA1 | 41277974 | rs3785546 | 17 | 41216933 | C | T | 0.360835 | -0.122378 | 0.0402184 | 0.00234347 | 0.4659 | 0.034057 | 1.34E-42 | -0.26267 | 0.0884338 | 0.002975605 | 0.4102552 | 20 | 0.472230432 |
|  | cg02716646 | 2 | POMC | 25384293 | rs6751851 | 2 | 25381148 | A | G | 0.0526839 | 0.255123 | 0.0783732 | 0.001133 | -0.720325 | 0.0994187 | 4.31E-13 | -0.354178 | 0.119279 | 0.002984686 | 0.09511481 | 16 | 0.472230432 |
|  | cg23533285 | 6 | HLA-B | 31322348 | rs2523579 | 6 | 31328517 | T | C | 0.270378 | 0.121022 | 0.0401707 | 0.00258929 | 0.523644 | 0.0324833 | 1.83E-58 | 0.231115 | 0.0780419 | 0.003062233 | 0.7468417 | 20 | 0.472230432 |
|  | cg14518907 | 6 | EHMT2 | 31860292 | rs589428 | 6 | 31848220 | T | G | 0.27833 | 0.119134 | 0.0402866 | 0.00310477 | -0.438281 | 0.0325677 | 2.78E-41 | -0.271821 | 0.0941126 | 0.00387388 | 0.4188734 | 20 | 0.472230432 |
|  | cg02757179 | 2 | POMC | 25384809 | rs17039879 | 2 | 25371477 | A | G | 0.0397614 | 0.401755 | 0.137684 | 0.00352338 | -1.4695 | 0.0977966 | 4.95E-51 | -0.273396 | 0.0954447 | 9.98469E-05 | 0.1054922 | 20 | 0.472230432 |
|  | cg12394289 | 6 | EHMT2 | 31856706 | rs9378275 | 6 | 32803999 | T | A | 0.027833 | 0.223231 | 0.0750901 | 0.0029506 | 1.31167 | 0.125127 | 1.04E-25 | 0.170188 | 0.0595053 | 0.004235684 | 0.4311403 | 3 | 0.472230432 |
|  | cg06846259 | 2 | POMC | 25384654 | rs6713396 | 2 | 25384705 | C | T | 0.0387674 | 0.400513 | 0.137893 | 0.00367824 | -1.4602 | 0.0977435 | 1.83E-50 | -0.274286 | 0.0962026 | 0.00435638 | 0.1577411 | 20 | 0.472230432 |
|  | cg26706521 | 6 | HLA-B | 31325085 | rs9266242 | 6 | 31325620 | T | C | 0.270378 | 0.121178 | 0.0423751 | 0.00424121 | 0.782343 | 0.0307905 | 2.03E-142 | 0.154891 | 0.0545063 | 0.004487227 | 0.131786 | 20 | 0.472230432 |
|  | cg04981410 | 6 | HLA-B | 31325427 | rs2523587 | 6 | 31327400 | T | C | 0.269384 | 0.121373 | 0.0423436 | 0.00415193 | -0.617162 | 0.0298875 | 9.87E-95 | -0.196663 | 0.069268 | 0.004523236 | 0.06962941 | 20 | 0.472230432 |
|  | cg10466124 | 6 | HLA-DRB5 | 32498285 | rs9271406 | 6 | 32587588 | A | G | 0.49006 | 0.111451 | 0.0389445 | 0.0042125 | 0.658387 | 0.0300641 | 2.63E-106 | 0.169279 | 0.0596543 | 0.004544502 | 0.904659 | 20 | 0.472230432 |
|  | cg17554194 | 6 | HLA-B | 31324972 | rs9266242 | 6 | 31325620 | T | C | 0.270378 | 0.121178 | 0.0423751 | 0.00424121 | -0.584951 | 0.0298663 | 2.05E-85 | -0.207159 | 0.0732102 | 0.004659914 | 0.2821688 | 20 | 0.472230432 |
|  | cg26887085 | 6 | CNR1 | 88861706 | rs9450898 | 6 | 88864063 | T | C | 0.156064 | -0.174706 | 0.0590778 | 0.0031042 | 0.381582 | 0.0408437 | 9.41E-21 | -0.457847 | 0.162394 | 0.004812115 | 0.7908665 | 11 | 0.472230432 |
|  | cg18511546 | 6 | HLA-C | 31238388 | rs2523578 | 6 | 31328542 | G | A | 0.201789 | 0.12652 | 0.0449186 | 0.00485277 | 0.884449 | 0.0307999 | 2.41E-181 | 0.14305 | 0.0510308 | 0.005059858 | 0.1777831 | 20 | 0.472230432 |
|  | cg11728928 | 17 | CCL3 | 34415818 | rs8064426 | 17 | 34819750 | A | G | 0.178926 | 0.128914 | 0.0461039 | 0.00517154 | 0.969384 | 0.0497348 | 1.31E-84 | 0.132985 | 0.0480469 | 0.005643156 | 0.1890169 | 20 | 0.472230432 |
|  | cg17974398 | 6 | HLA-C | 31239324 | rs9266245 | 6 | 31325702 | A | G | 0.201789 | 0.126296 | 0.0449578 | 0.00496649 | -0.516975 | 0.0338716 | 1.35E-52 | -0.244298 | 0.0884239 | 0.005730708 | 0.1115948 | 20 | 0.472230432 |
|  | cg16361343 | 6 | EHMT2 | 31863472 | rs589428 | 6 | 31848220 | T | G | 0.27833 | 0.119134 | 0.0402866 | 0.00310477 | -0.250245 | 0.0331325 | 4.26E-14 | -0.476069 | 0.172888 | 0.005893912 | 0.1704621 | 20 | 0.472230432 |
|  | cg20559215 | 6 | MAPK14 | 36076933 | rs3804452 | 6 | 36076934 | A | G | 0.119284 | 0.152266 | 0.0554191 | 0.00600441 | -1.80927 | 0.0375894 | 0 | -0.0841588 | 0.0306805 | 0.006086751 | 0.1807861 | 20 | 0.472230432 |
|  | cg23977453 | 3 | VHL | 10182923 | rs279545 | 3 | 9972493 | G | A | 0.190855 | -0.153389 | 0.0548819 | 0.00519159 | 0.434393 | 0.0379475 | 2.43E-30 | -0.353111 | 0.130053 | 0.006624839 | 0.772625 | 20 | 0.488477796 |
|  | cg19393006 | 9 | TSC1 | 135820767 | rs11243940 | 9 | 135821372 | G | A | 0.206759 | -0.150245 | 0.0552056 | 0.00649756 | -0.880058 | 0.0353159 | 4.56E-137 | 0.170722 | 0.0631025 | 0.00682085 | 0.9031904 | 20 | 0.488477796 |
|  | cg13778567 | 6 | HLA-DQA1 | 32609783 | rs28366319 | 6 | 32561495 | A | G | 0.300199 | -0.123827 | 0.0467008 | 0.00801346 | 1.09606 | 0.0248368 | 0 | -0.112975 | 0.0426847 | 0.008127582 | 0.9999129 | 20 | 0.560502136 |
|  | cg14061503 | 9 | TSC1 | 135820653 | rs7874234 | 9 | 135813001 | T | C | 0.207753 | -0.153076 | 0.0531156 | 0.0039523 | -0.238199 | 0.0370003 | 1.21E-10 | 0.642639 | 0.244312 | 0.008528463 | 0.7054209 | 15 | 0.566183091 |
|  | cg26674160 | 21 | S100B | 48025100 | rs13047696 | 21 | 48027177 | G | A | 0.314115 | 0.10591 | 0.0405795 | 0.00905607 | 0.668764 | 0.0321558 | 4.55E-96 | 0.158367 | 0.0611543 | 0.009607996 | 0.06975897 | 20 | 0.569789492 |
| HLA DR on CD33br HLA DR+ CD14dim | cg19624354 | 11 | NCAM1 | 113113890 | rs635596 | 11 | 113113515 | G | A | 0.163022 | -0.126642 | 0.0490584 | 0.00983875 | 1.2164 | 0.0371111 | 1.24E-235 | -0.104112 | 0.0404557 | 0.010068 | 0.1671415 | 20 | 0.016157296 |
|  | cg08743050 | 11 | NCAM1 | 113113936 | rs635596 | 11 | 113113515 | G | A | 0.163022 | -0.126642 | 0.0490584 | 0.00983875 | 1.17232 | 0.0380408 | 1.53E-208 | -0.108027 | 0.0419938 | 0.01009831 | 0.1606447 | 20 | 0.016157296 |
|  | cg03228353 | 1 | FCGR3A | 161520094 | rs7539053 | 1 | 161659845 | T | C | 0.218688 | 0.0989426 | 0.0454841 | 0.029606 | -0.238081 | 0.0387771 | 8.27E-10 | -0.415584 | 0.202681 | 0.04032242 | 0.7862339 | 7 | 0.043010581 |
| HLA DR on CD33- HLA DR+ | cg05030953 | 6 | HLA-C | 31241000 | rs9264486 | 6 | 31232482 | A | T | 0.122266 | 0.174935 | 0.0522231 | 0.000808816 | 1.04119 | 0.0379504 | 1.03E-165 | 0.168014 | 0.0505296 | 0.000883962 | 0.4960896 | 20 | 0.349991952 |
|  | cg08309069 | 6 | HLA-C | 31240651 | rs9264486 | 6 | 31232482 | A | T | 0.122266 | 0.174935 | 0.0522231 | 0.000808816 | 0.82349 | 0.040246 | 4.76E-93 | 0.212431 | 0.064261 | 0.000947204 | 0.6151317 | 20 | 0.349991952 |
|  | cg10045137 | 2 | POMC | 25383940 | rs6545951 | 2 | 25343125 | T | C | 0.0487078 | 0.254232 | 0.078579 | 0.00121484 | -1.71319 | 0.0960368 | 3.53E-71 | -0.148397 | 0.0466153 | 8.45539E-05 | 0.13172 | 20 | 0.373090509 |
|  | cg25067162 | 17 | BRCA1 | 41277974 | rs3785546 | 17 | 41216933 | C | T | 0.360835 | -0.122378 | 0.0402184 | 0.00234347 | 0.4659 | 0.034057 | 1.34E-42 | -0.26267 | 0.0884338 | 0.002975605 | 0.4102552 | 20 | 0.373090509 |
|  | cg02716646 | 2 | POMC | 25384293 | rs6751851 | 2 | 25381148 | A | G | 0.0526839 | 0.255123 | 0.0783732 | 0.001133 | -0.720325 | 0.0994187 | 4.31E-13 | -0.354178 | 0.119279 | 0.002984686 | 0.09511481 | 16 | 0.373090509 |
|  | cg23533285 | 6 | HLA-B | 31322348 | rs2523579 | 6 | 31328517 | T | C | 0.270378 | 0.121022 | 0.0401707 | 0.00258929 | 0.523644 | 0.0324833 | 1.83E-58 | 0.231115 | 0.0780419 | 0.003062233 | 0.7468417 | 20 | 0.373090509 |
|  | cg02757179 | 2 | POMC | 25384809 | rs17039879 | 2 | 25371477 | A | G | 0.0397614 | 0.401755 | 0.137684 | 0.00352338 | -1.4695 | 0.0977966 | 4.95E-51 | -0.273396 | 0.0954447 | 9.98469E-05 | 0.1054922 | 20 | 0.373090509 |
|  | cg06846259 | 2 | POMC | 25384654 | rs6713396 | 2 | 25384705 | C | T | 0.0387674 | 0.400513 | 0.137893 | 0.00367824 | -1.4602 | 0.0977435 | 1.83E-50 | -0.274286 | 0.0962026 | 0.00435638 | 0.1577411 | 20 | 0.373090509 |
|  | cg26706521 | 6 | HLA-B | 31325085 | rs9266242 | 6 | 31325620 | T | C | 0.270378 | 0.121178 | 0.0423751 | 0.00424121 | 0.782343 | 0.0307905 | 2.03E-142 | 0.154891 | 0.0545063 | 0.004487227 | 0.131786 | 20 | 0.373090509 |
|  | cg23870181 | 6 | HLA-DQA2 | 32713044 | rs75565243 | 6 | 31463379 | A | G | 0.0298211 | 0.261619 | 0.089954 | 0.00363321 | -1.47721 | 0.112803 | 3.49E-39 | -0.177103 | 0.0623782 | 0.004522852 | 0.1991702 | 4 | 0.373090509 |
|  | cg04981410 | 6 | HLA-B | 31325427 | rs2523587 | 6 | 31327400 | T | C | 0.269384 | 0.121373 | 0.0423436 | 0.00415193 | -0.617162 | 0.0298875 | 9.87E-95 | -0.196663 | 0.069268 | 0.004523236 | 0.06962941 | 20 | 0.373090509 |
|  | cg10466124 | 6 | HLA-DRB5 | 32498285 | rs9271406 | 6 | 32587588 | A | G | 0.49006 | 0.111451 | 0.0389445 | 0.0042125 | 0.658387 | 0.0300641 | 2.63E-106 | 0.169279 | 0.0596543 | 0.004544502 | 0.904659 | 20 | 0.373090509 |
|  | cg23221363 | 6 | HLA-DQA2 | 32710933 | rs75565243 | 6 | 31463379 | A | G | 0.0298211 | 0.261619 | 0.089954 | 0.00363321 | -1.4455 | 0.112999 | 1.81E-37 | -0.180989 | 0.0638185 | 0.004568372 | 0.165753 | 4 | 0.373090509 |
|  | cg17554194 | 6 | HLA-B | 31324972 | rs9266242 | 6 | 31325620 | T | C | 0.270378 | 0.121178 | 0.0423751 | 0.00424121 | -0.584951 | 0.0298663 | 2.05E-85 | -0.207159 | 0.0732102 | 0.004659914 | 0.2821688 | 20 | 0.373090509 |
|  | cg18511546 | 6 | HLA-C | 31238388 | rs2523578 | 6 | 31328542 | G | A | 0.201789 | 0.12652 | 0.0449186 | 0.00485277 | 0.884449 | 0.0307999 | 2.41E-181 | 0.14305 | 0.0510308 | 0.005059858 | 0.1777831 | 20 | 0.373090509 |
|  | cg15665090 | 6 | HSPA1L | 31778371 | rs2227956 | 6 | 31778272 | G | A | 0.157058 | 0.152871 | 0.0502289 | 0.00233846 | 0.267455 | 0.0400837 | 2.52E-11 | 0.571577 | 0.206417 | 0.005622289 | 0.8207486 | 12 | 0.373090509 |
|  | cg11728928 | 17 | CCL3 | 34415818 | rs8064426 | 17 | 34819750 | A | G | 0.178926 | 0.128914 | 0.0461039 | 0.00517154 | 0.969384 | 0.0497348 | 1.31E-84 | 0.132985 | 0.0480469 | 0.005643156 | 0.1890169 | 20 | 0.373090509 |
|  | cg17974398 | 6 | HLA-C | 31239324 | rs9266245 | 6 | 31325702 | A | G | 0.201789 | 0.126296 | 0.0449578 | 0.00496649 | -0.516975 | 0.0338716 | 1.35E-52 | -0.244298 | 0.0884239 | 0.005730708 | 0.1115948 | 20 | 0.373090509 |
|  | cg20559215 | 6 | MAPK14 | 36076933 | rs3804452 | 6 | 36076934 | A | G | 0.119284 | 0.152266 | 0.0554191 | 0.00600441 | -1.80927 | 0.0375894 | 0 | -0.0841588 | 0.0306805 | 0.006086751 | 0.1807861 | 20 | 0.373090509 |
|  | cg02293354 | 6 | HLA-DQA2 | 32711008 | rs9271406 | 6 | 32587588 | A | G | 0.49006 | 0.111451 | 0.0389445 | 0.0042125 | 0.245329 | 0.0322401 | 2.75E-14 | 0.454292 | 0.169599 | 0.007392578 | 0.8912678 | 20 | 0.373090509 |
|  | cg12172531 | 6 | NOTCH4 | 32184296 | rs2857598 | 6 | 31563228 | A | C | 0.10835 | 0.163932 | 0.0548263 | 0.00278952 | 0.264895 | 0.0451945 | 4.59E-09 | 0.618857 | 0.232349 | 0.007733865 | 0.341737 | 20 | 0.373090509 |
|  | cg13778567 | 6 | HLA-DQA1 | 32609783 | rs28366319 | 6 | 32561495 | A | G | 0.300199 | -0.123827 | 0.0467008 | 0.00801346 | 1.09606 | 0.0248368 | 0 | -0.112975 | 0.0426847 | 0.008127582 | 0.9999129 | 20 | 0.373090509 |
|  | cg10123514 | 6 | HLA-DMB | 32904061 | rs145364886 | 6 | 32946979 | T | C | 0.0308151 | 0.7121 | 0.269376 | 0.00820484 | -1.58608 | 0.0798706 | 9.37E-88 | -0.448969 | 0.171336 | 0.008782648 | 0.1410212 | 20 | 0.373090509 |
|  | cg00988577 | 6 | HLA-DOA | 32978548 | rs2523585 | 6 | 31327504 | T | C | 0.275348 | 0.121393 | 0.0423434 | 0.00414562 | -0.207954 | 0.0323853 | 1.35E-10 | -0.583749 | 0.222991 | 0.008849606 | 0.1571288 | 16 | 0.373090509 |
|  | cg03100814 | 6 | BTNL2 | 32367672 | rs9271718 | 6 | 32593465 | T | A | 0.365805 | -0.130019 | 0.0453907 | 0.00417753 | 0.211332 | 0.0332881 | 2.17E-10 | -0.615236 | 0.235634 | 0.009028403 | 0.998154 | 20 | 0.373090509 |
|  | cg26674160 | 21 | S100B | 48025100 | rs13047696 | 21 | 48027177 | G | A | 0.314115 | 0.10591 | 0.0405795 | 0.00905607 | 0.668764 | 0.0321558 | 4.55E-96 | 0.158367 | 0.0611543 | 0.009607996 | 0.06975897 | 20 | 0.373090509 |
| GWAS: genome-wide association study.  mQTL: methylation quantitative trait loci.  SMR: summary-data-based Mendelian randomization.  HEIDI: heterogeneity in dependent instruments. Only genome-wide significant mQTLs (P<5E-8) are taken into the analysis. Only results with a significance level (P-value) less than 0.01 are presented, We focus on SNP-gene combinations with PSMR < genome-wide significance Benjamin Hochberg correction threshold of PFDR＜0.05, and survived after the heterogeneity test (PHEIDI＞0.05). β in GWAS association, regression coefficient of cancer on SNP, log(OR).  SE, standard error.  β in eQTL association, regression coefficient of gene expression on SNP.  β in SMR association, regression coefficient of cancer on gene expression. | | | | | | | | | | | | | | | | | | | | | | |
